# 4′‐SCF_3_‐Labeling Constitutes a Sensitive ^19^F NMR Probe for Characterization of Interactions in the Minor Groove of DNA

**DOI:** 10.1002/anie.202201848

**Published:** 2022-10-19

**Authors:** Qiang Li, Marko Trajkovski, Chaochao Fan, Jialiang Chen, Yifei Zhou, Kuan Lu, Hongjun Li, Xuncheng Su, Zhen Xi, Janez Plavec, Chuanzheng Zhou

**Affiliations:** ^1^ State Key Laboratory of Elemento-Organic Chemistry and Department of Chemical Biology College of Chemistry Nankai University Tianjin 300071 China; ^2^ Slovenian NMR Centre National Institute of Chemistry Hajdrihova 19 SI-1000 Ljubljana Slovenia

**Keywords:** ^19^F NMR, DNA Structure, Fluorinated Nucleotides, Nucleic Acids

## Abstract

Fluorinated nucleotides are invaluable for ^19^F NMR studies of nucleic acid structure and function. Here, we synthesized 4′‐SCF_3_‐thymidine (T${}^{4{}^{'}\text{-}\mathrm{SCF}{}_{3}}$ ) and incorporated it into DNA by means of solid‐phase DNA synthesis. NMR studies showed that the 4′‐SCF_3_ group exhibited a flexible orientation in the minor groove of DNA duplexes and was well accommodated by various higher order DNA structures. The three magnetically equivalent fluorine atoms in 4′‐SCF_3_‐DNA constitute an isolated spin system, offering high ^19^F NMR sensitivity and excellent resolution of the positioning of T${}^{4{}^{'}\text{-}\mathrm{SCF}{}_{3}}$ within various secondary and tertiary DNA structures. The high structural adaptability and high sensitivity of T${}^{4{}^{'}\text{-}\mathrm{SCF}{}_{3}}$ make it a valuable ^19^F NMR probe for quantitatively distinguishing diverse DNA structures with single‐nucleotide resolution and for monitoring the dynamics of interactions in the minor groove of double‐stranded DNA.

## Graphical abstract

Thymidine modified with a 4′‐SCF_3_ group exhibits a rather flexible orientation in the minor groove of DNA duplexes and is accommodated well by different higher order DNA structures. The high structural adaptability and high ^19^F NMR sensitivity of 4′‐SCF_3_‐thymidine make it an excellent probe for quantitatively distinguishing various DNA structures and for monitoring the dynamics of DNA‐protein interactions in the minor groove by means of ^19^F NMR spectroscopy.

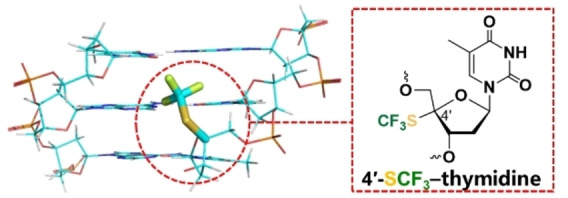

## Introduction

Because ^19^F exhibits unique biochemical and biophysical properties, including excellent biocompatibility, high natural sensitivity, and a wide chemical shift dispersion, introduction of ^19^F into nucleic acids offers an ideal approach for studying their structure and function by means of ^19^F NMR spectroscopy.[^1^, ^2^, ^3^] Fluorine‐containing functional groups, such as 2′‐F,[^4^, ^5^] 2′‐SCF_3_,[^6^, ^7^] and 2′‐OCF_3_,^[8]^ are generally introduced at C2′ of the ribose moiety.[^4^, ^5^, ^6^, ^7^, ^8^, ^9^, ^10^, ^11^] Compared with RNA structure, DNA structure is much more profoundly perturbed by 2′‐F or 2′‐CF_3_ modifications, which makes these modifications less attractive for ^19^F NMR‐based analysis of DNA structure‐function relationships.[^4^, ^7^] In contrast, fluorine‐containing nucleobases such as 5‐F‐uracil, 5‐CF_3_‐uracil, and 2‐F‐adenine, are adaptable ^19^F NMR probes for both RNA and DNA studies.[^12^, ^13^, ^14^, ^15^, ^16^] Recently, fluorine‐containing functional groups have been introduced at C8 of purine and at the 5′ terminus of DNA strands.[^17^, ^18^, ^19^, ^20^, ^21^, ^22^] These modifications show promise for the study of noncanonical DNA structures in living cells by means of ^19^F NMR spectroscopy.

Alternatively, introducing fluorine to C4′ of ribose or deoxyribose is a striking strategy for structural and functional studies, for several reasons. First, compared with modification at C2′, modification at C4′ seems to have a weaker stereoelectronic effect on the ribose conformation. Second, because C4′ is located on the edge of the sugar‐phosphate backbone, modification at C4′ has minimal effect on the tertiary structure of nucleic acids. Although numerous C4′‐modified nucleotides have been synthesized and have found a wide variety of applications,[^23^, ^24^, ^25^, ^26^, ^27^, ^28^, ^29^, ^30^] there have been only a few reports of the synthesis of C4′‐fluorinated nucleotides as probes for ^19^F NMR studies of nucleic acid structure and function.^[31]^

Recently, we synthesized 4′‐F‐RNA and 4′‐F‐DNA.[^32^, ^33^] 4′‐F‐RNA is an ideal probe for studying RNA structure and dynamics as well as RNA‐protein interactions. In contrast, 4′‐F‐DNA has limited potential because ^19^F NMR chemical shifts are only weakly sensitive to local changes in DNA structure.^[33]^ In addition, the endo‐anomeric effect along the F−C4′−O4′ fragment and the F−C4′−C3′−O3′ gauche effect on conformational preferences cannot be excluded. We hypothesized that 4′‐SCF_3_‐DNA would be a better probe for providing insight into DNA structure and distinguishing its polymorphic forms. The three magnetically equivalent fluorine atoms in 4′‐SCF_3_‐DNA constitute an isolated spin system, which could be expected to result in high ^19^F NMR sensitivity. In addition, introduction of an S atom bridging C4′ and the CF_3_ group would greatly increase the conformational freedom of the CF_3_ group, making 4′‐SCF_3_‐modified nucleotides attractive probes for characterization of interactions at the minor groove of DNA. Herein, we report the synthesis of a series of oligonucleotides (ODNs) modified with 4′‐SCF_3_‐thymidine (T${}^{4{}^{'}\text{-}\mathrm{SCF}{}_{3}}$ ) and their use for characterization of DNA structure and dynamics by means of ^19^F NMR spectroscopy.

## Results and Discussion

### Synthesis of 4′‐SCF_3_‐thymidine Phosphoramidite

**Scheme 1:**
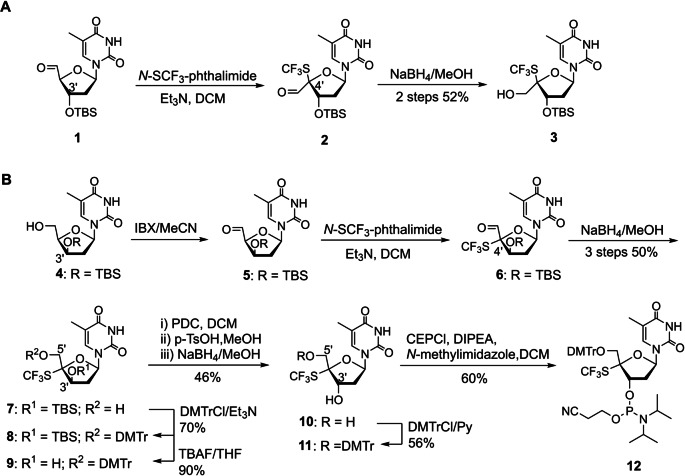
Synthesis of T${}^{4{}^{'}\text{-}\mathrm{SCF}{}_{3}}$ phosphoramidite. A) Synthesis of 2′‐deoxy‐4′‐SCF_3_‐α‐l‐xylofuranosyl‐thymine analogue **3**. B) Synthesis of 4′‐SCF_3_‐thymidine phosphoramidite **12**. Abbreviations: CEPCl, *N*,*N*‐diisopropylamino‐2‐cyanoethoxychlorophosphine; DCM, dichloromethane; DIPEA, *N*,*N*‐diisopropylethylamine; DMTr, 4,4′‐dimethoxytrityl; IBX, 2‐iodoxybenzoic acid; PDC, pyridinium dichromate; Py, pyridine; p‐TsOH, p‐toluenesulfonic acid; TBAF, n‐Bu_4_NF; TBS, *t*‐butyldimethylsilyl; THF, tetrahydrofuran.

We synthesized target molecule T${}^{4{}^{'}\text{-}\mathrm{SCF}{}_{3}}$ by introducing a SCF_3_ group at C4′ of thymidine by means of electrophilic trifluoromethanesulfenylation.[^34^, ^35^] We began by treating 5′‐aldehyde‐thymidine **1**, which has a 3′‐OTBS group on the α face of the sugar ring, with *N*‐SCF_3_‐phthalimide as a sulfenylating reagent in the presence of Et_3_N. Unfortunately, 2′‐deoxy‐4′‐SCF_3_‐α‐l‐xylofuranosyl‐thymine analogue **2** was obtained as the sole product (Scheme 1A). Reduction of **2** with NaBH_4_ afforded **3**, and its C4′ configuration was unambiguously confirmed by NMR (see Supporting Information). We assumed that the steric bulk of the 3′‐OTBS group prevented the sulfenylating reagent from attacking the α face of **1**.

To circumvent this problem, we treated compound **4** with 2‐iodoxybenzoic acid to prepare **5**, in which the 3′‐OTBS group is on the β face of the sugar ring (Scheme 1B). Reaction of **5** with *N*‐SCF_3_‐phthalimide stereoselectively gave **6**, which was reduced with NaBH_4_ to afford 2′‐deoxy‐4′‐SCF_3_‐β‐d‐xylofuranosyl‐thymine analogue **7** as the predominant product. Oxidation and subsequent reduction were used to invert the configuration at C3′.^[36]^ Specifically, the 5′‐OH of **7** was protected with 4,4′‐dimethoxytritylchloride, and the 3′‐OH was deprotected by treatment with neutralized tetrabutylammonium fluoride to afford **9**. The 3′‐OH of **9** was then oxidized to the corresponding ketone with pyridinium dichromate, the 5′‐OH was deprotected under acidic conditions, and the ketone at C3′ was reduced with NaBH_4_ to afford 4′‐SCF_3_‐thymidine analogue **10** as the predominant product. If the protecting group on the 5′‐OH was not removed, the reduction stereoselectivity was poor, and the yield of **10** was low. Finally, the 5′‐OH of **10** was reprotected with 4,4′‐dimethoxytritylchloride, and the obtained product (**11**) was transformed to phosphoramidite **12** by means of a conventional method.

### Synthesis and pH Stability of T${}^{4{}^{'}\text{-}\mathrm{SCF}{}_{3}}$ ‐Modified ODNs

The incorporation of T${}^{4{}^{'}\text{-}\mathrm{SCF}{}_{3}}$ phosphoramidite **12** into ODNs was performed on a DNA solid‐phase synthesizer. Standard reagents and synthesis cycles were utilized, except that the coupling time for **12** was extended from 25 s to 10 min, and *N,N*‐dimethylformamidine‐protected dG phosphoramidite was employed. Incorporation of T${}^{4{}^{'}\text{-}\mathrm{SCF}{}_{3}}$ was as efficient as incorporation of native thymidine in the series of ODNs described herein. After being synthesized, the ODNs were deprotected by treatment with 28 % (w/w) aqueous ammonia at room temperature, and the deprotected ODNs were purified by polyacrylamide gel electrophoresis. Yields of isolated ODNs **13**–**18** prepared on a 1 μmol scale were sufficient for preparation of NMR samples with submillimolar (0.1–0.7 mM) concentrations. The structural integrity of ODNs **13**–**18** was confirmed by ultraperformance liquid chromatography‐mass spectrometry (Table 1).

**Table 1:** Sequences and integrity of T${}^{4{}^{'}\text{-}\mathrm{SCF}{}_{3}}$ ‐modified ODNs and their natural counterparts.

ODN	Sequence (5′‐3′)^[a]^	[M−H]^−[b]^	
		Calc.	Found
ODN **13**	d(CTT CAT TTT TTC TTC)	4449.0	4450.1
ODN **14**	d(CTT CAT T X T TTC TTC)	4548.9	4549.9
ODN **15**	d(CTT CA X T X T X TC TTC)	4748.9	4749.6
ODN **16**	d(CT X CA X T X T X TC X TC)	4948.9	4950.3
ODN **17**	d(CCA T X A TAG C)	3086.0	3088.0
ODN **18**	d(CCA TTA TAG C)	2986.0	2985.9

[a] X =T${}^{4{}^{'}\text{-}\mathrm{SCF}{}_{3}}$ . [b] Molecular mass was determined by means of electrospray ionization quadrupole time‐of‐flight mass spectrometry in negative‐ion mode.

The overall yield of ODN **16**, which contains five T${}^{4{}^{'}\text{-}\mathrm{SCF}{}_{3}}$ modifications, was 10 %, which was lower than the yields of the other ODNs (≈15 %). In addition, the harsh deprotection conditions (e.g., with a 1 : 1 [v/v] mixture of 40 % [w/w] aqueous methylamine and 28 % [w/w] aqueous ammonia) decreased the yield of ODN **16** to <10 %. Therefore, we investigated the chemical stability of the T${}^{4{}^{'}\text{-}\mathrm{SCF}{}_{3}}$ ‐modified ODNs under various pH conditions. Specifically, we incubated 5′‐fluorescein‐labeled ODN **17** separately in acidic (pH 5.2), neutral (pH 7.0), and alkaline (pH 10.0) buffers. Oligonucleotide cleavage at the modification site in ODN **17** was observed in the alkaline buffer (4 % degradation after 23 h of incubation at room temperature, Figure S1) but not in the acidic or neutral buffer. Therefore, although the T${}^{4{}^{'}\text{-}\mathrm{SCF}{}_{3}}$ ‐modified ODNs decomposed to some extent during the postsynthesis treatment with ammonia, they were stable in neutral buffer, which allowed us to characterize their structures and demonstrate some potential applications.

### Sugar Conformations in Single‐ and Double‐Stranded DNA Modified with T${}^{4{}^{'}\text{-}\mathrm{SCF}{}_{3}}$

To assess how the 4′‐SCF_3_ substituent modulated the pseudorotational equilibrium of the 2′‐deoxyribose moiety, we studied the sugar conformation of 4′‐SCF_3_‐thymidine **10** on the basis of the well‐resolved ^3^ *J*_H‐H_ coupling constants extracted from the ^1^H NMR spectrum (Table S1). Calculations using two empirical formulas (Table S1) suggested that sugar puckering in **10** was highly biased (>90 %) toward *North‐type* conformations.[^23^, ^37^] Moreover, the sugar in the T${}^{4{}^{'}\text{-}\mathrm{SCF}{}_{3}}$ residue in trinucleotide **19** (5′‐TT${}^{4{}^{'}\text{-}\mathrm{SCF}{}_{3}}$ A) adopted approximately 90 % *North‐type* conformation. In contrast, the sugar in the middle thymidine residue of native trinucleotide **20** (5′‐TTA) existed predominantly in the *South‐type* conformation (Table S1, Figures S2–S4). These results indicate that the 4′‐SCF_3_ group exerted similar stereoelectronic effects on the conformation of the 2′‐deoxyribose moiety of T${}^{4{}^{'}\text{-}\mathrm{SCF}{}_{3}}$ in its monomeric form and when it was incorporated in a short oligonucleotide. In tending to drive the *North*‐*South* pseudorotational equilibrium toward the *North* confirmation, 4′‐SCF_3_ is analogous to 4′‐F and 4′‐OMe[^23^, ^33^] but opposite to 4′‐Me^[29]^ and 4′‐CF_3_.^[30]^

**Figure 1:**
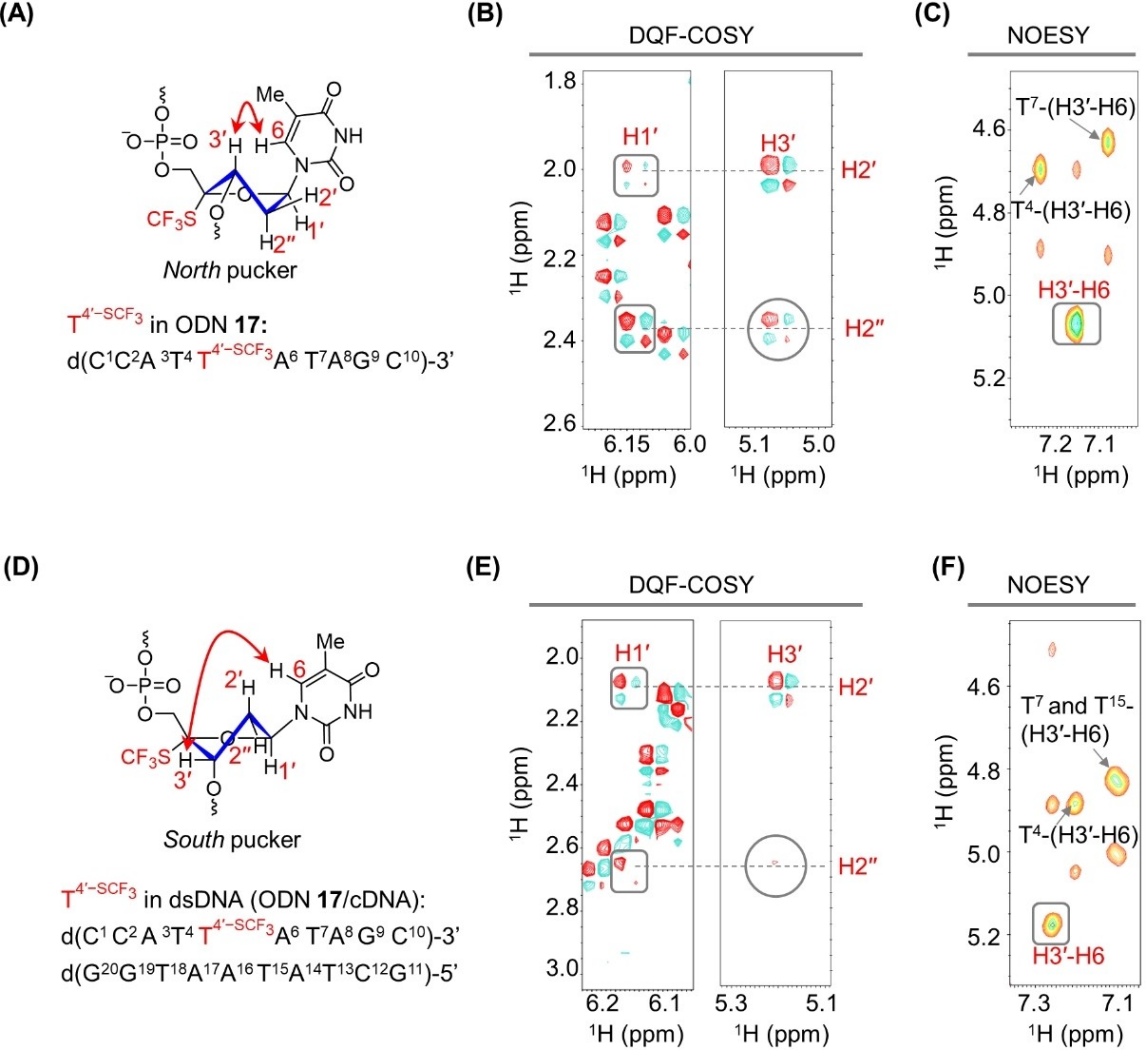
Conformational analysis of the 2′‐deoxyribose moiety of T${}^{4{}^{'}\text{-}\mathrm{SCF}{}_{3}}$ within single‐ and double‐stranded DNA. A) Stereochemical relationships within the *North‐type* 2′‐deoxyribose conformation of T${}^{4{}^{'}\text{-}\mathrm{SCF}{}_{3}}$ in single‐stranded ODN **17**. B) Detail of the DQF‐COSY spectrum of T${}^{4{}^{'}\text{-}\mathrm{SCF}{}_{3}}$ ‐modified ODN **17**. The rectangles and the circle indicate the key H1′‐H2′, H1′‐H2′′, and H2′′‐H3′ cross‐peaks. C) Detail of the NOESY spectrum (τ_m_=400 ms) of ODN **17** with highlighted H3′‐H6 cross‐peak. Sample conditions for panels B and C: 0.7 mM ODN **17**, 10 mM Na‐phosphate, pH 7.0, 298 K, 100 % D_2_O, 600 MHz. D) Depiction of the *South‐type* 2∋‐deoxyribose conformation of T${}^{4{}^{'}\text{-}\mathrm{SCF}{}_{3}}$ in the ODN **17**/cDNA duplex. E) Detail of the DQF‐COSY spectrum of the T${}^{4{}^{'}\text{-}\mathrm{SCF}{}_{3}}$ ‐modified ODN **17**/cDNA duplex with key cross‐peaks indicated by two rectangles and a circle. F) Detail of the NOESY spectrum (τ_m_=400 ms) of the ODN **17**/cDNA duplex with highlighted H3′‐H6 cross‐peak. Sample conditions for panels E and F: 0.4 mM ODN **17**/cDNA per strand, 10 mM Na‐phosphate, pH 7.0, 278 K, 100 % D_2_O, 600 MHz.

In the context of a longer single‐stranded ODN (i.e., **17**), several observations suggested that the sugar in T${}^{4{}^{'}\text{-}\mathrm{SCF}{}_{3}}$ predominantly adopted a *North‐type* conformation (Figure 1A). First, in the DQF‐COSY spectrum of **17**, the cross‐peak between H1′ and H2′ was less intense than that between H1′ and H2′′(Figure 1B), indicating that *J*_H1′‐H2′_ was smaller than *J*_H1′‐H2′′_, a result that is consistent with the predominance of the *North‐type* conformation. This was corroborated by the *J*_H1′‐H2′_ and *J*_H1′‐H2′′_ values of 5.3 and 8.1 Hz, respectively, observed in the ^1^H NMR spectrum of ODN **17** (Figure S5). Second, a clear cross‐peak between H2“ and H3′ and a relatively large *J*_H2′′‐H3′_ value were consistent with the predominance of the *North‐type* pseudorotamer.[^38^, ^39^] Third, in the NOESY spectrum of **17** (τ_m_=400 ms), the intensity of the H3′‐H6 correlation for the T${}^{4{}^{'}\text{-}\mathrm{SCF}{}_{3}}$ residue was approximately 3 times as high as that of native thymidine residues (cf. Figures 1C and S5–S7).^[40]^

The correlations between H1′, H2′′, and H3′ for T${}^{4{}^{'}\text{-}\mathrm{SCF}{}_{3}}$ in the DQF‐COSY spectrum of double‐stranded DNA (dsDNA) formed from ODN **17** and its complementary DNA (cDNA) differed dramatically from the correlations for single‐stranded ODN **17**. A strong H1′‐H2′ correlation along with weak H1′‐H2′′ and H2′′‐H3′ correlations (Figures 1E and S8) indicated that the sugar in T${}^{4{}^{'}\text{-}\mathrm{SCF}{}_{3}}$ adopted a *South‐type* conformation in the ODN **17**/cDNA duplex (Figure 1D, E). This observation was corroborated by the intensity of the H3′‐H6 cross‐peak in the NOESY spectrum of the duplex (τ_m_=400 ms), which was similar to that of the native thymidine residue in the corresponding dsDNA (cf. Figures 1F and S7).

Interestingly, in the DQF‐COSY spectrum of a ODN **17**/complementary RNA (cRNA) hybrid duplex, we observed large *J*_H1′‐H2′_ and *J*_H1′‐H2′′_ values, along with a large *J*_H2′‐H3′_ and a small *J*_H2′′‐H3′_. These results indicate that the sugar in T${}^{4{}^{'}\text{-}\mathrm{SCF}{}_{3}}$ adopted an *East‐type* conformation or was involved in a rapid equilibrium between *North‐* and *South‐type* conformations in the ODN **17**/cRNA hybrid duplex (Figure S9).[^41^, ^42^] Taken together, the above results indicate that the 4′‐SCF_3_ modification only slightly constrained the deoxyribose conformation. T${}^{4{}^{'}\text{-}\mathrm{SCF}{}_{3}}$ had the freedom to adjust its conformation so that it was well accommodated by the global structures of the nucleic acids.

### Structural Adaptability of 4′‐SCF_3_ in the Minor Groove of DNA Duplexes

**Figure 2:**
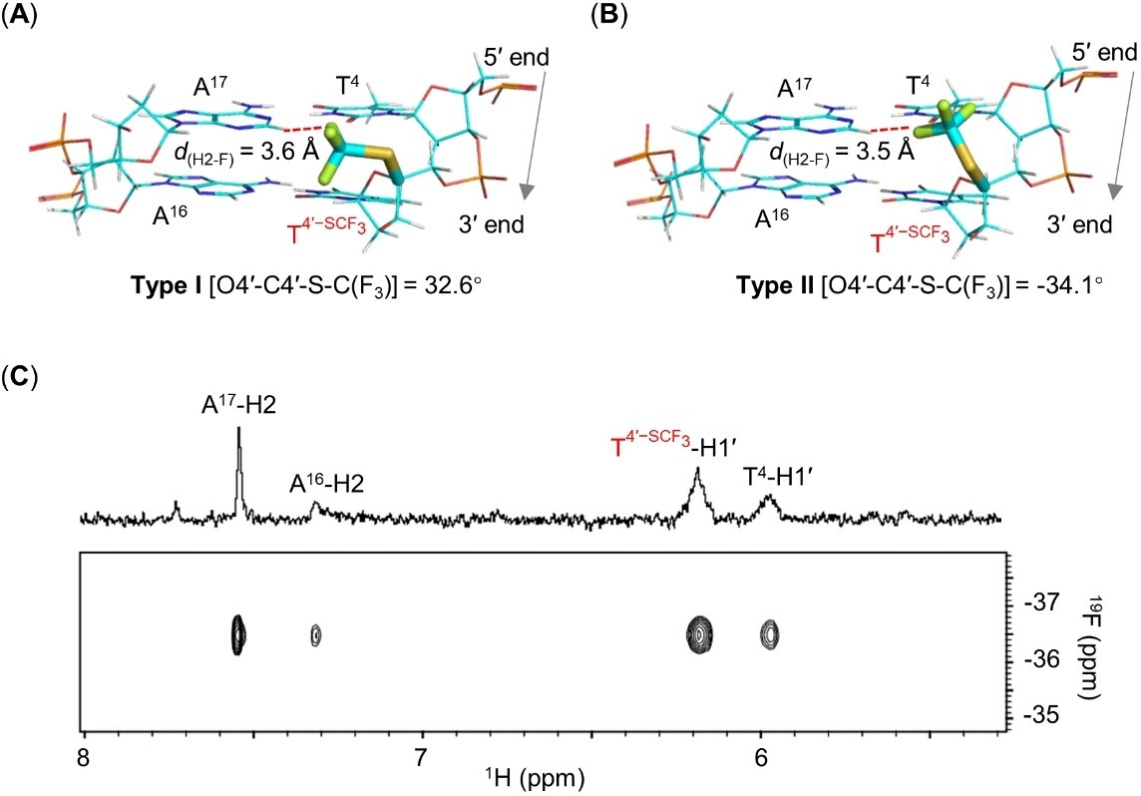
Type I and II orientations of 4′‐SCF_3_ in the minor groove of a T${}^{4{}^{'}\text{-}\mathrm{SCF}{}_{3}}$ ‐modified ODN **17**/cDNA duplex. A, B) Two base pairs in Type I and II clusters of structural ensembles of the ODN **17**/cDNA duplex showing the orientations of the 4′‐SCF_3_ groups (PDB ID: 7W0V). The shortest distance between the F atoms and A^17^‐H2 (*d*_(H2‐F_) is indicated. C) Detail of the ^19^F‐^1^H HOESY spectrum of ODN **17**/cDNA (τ_m_=400 ms) showing inter‐ and intrastrand correlations consistent with orientation(s) of the 4′‐SCF_3_ group in the minor groove. Sample conditions: 0.2 mM ODN per strand, 10 mM Na‐phosphate, pH 7.0, 278 K, 100 % D_2_O, 400 MHz.

We analyzed the structural features of the ODN **17**/cDNA duplex in depth by collecting well‐resolved NOESY, DQF‐COSY, and TOCSY two‐dimensional NMR spectra, which enabled us to assign all the resonances (Figure S10, Tables S2–S5). On the basis of the distance restraints obtained from the NOESY spectra and simulated annealing calculations, we calculated three‐dimensional structures of the ODN **17**/cDNA duplex. Ten lowest‐energy structures exhibited high convergence, with pairwise root‐mean‐square deviations of <0.4 Å (PDB ID: 7W0V; Figure S11 and Table S2). Overall, the structures had features of a standard B‐type duplex (Figures S12 and S13). The structural ensemble could be divided into two clusters, designated Type I and Type II, according to two distinct orientations of the 4′‐SCF_3_ group (Figures 2 and S11). These two types of ODN **17**/cDNA duplex structures were distinguished not by their energies but by the orientation of the 4′‐SCF_3_ group. The [O4′−C4′−S−C(F_3_)] torsion angle was 32.6° in the Type I structures, whereas it was −34.1° in the Type II structures. However, despite this difference, the 4′‐SCF_3_ group in both types pointed toward the minor groove of the 5′ flanking base pair (T^4^‐A^17^, Figure 2A, B). The close proximity of the fluorine atoms to A^17^‐H2 in both types of structures was confirmed by the high‐intensity cross‐peak in the two‐dimensional ^19^F‐^1^H HOESY spectrum (Figures 2C and S14).

Importantly, comparison of the calculated structures of the modified ODN **17**/cDNA duplex and the independently calculated structures of the unmodified ODN **18**/cDNA duplex (PDB ID: 7QA9) showed that they were very similar. Specifically, the root‐mean‐square deviations between the unmodified duplex and the Type I and Type II duplexes were <0.5 Å, and all the structures exhibited similar helical rises and twists, as well as torsion angles and groove widths (Figures S11–S13). Notably, all the deoxyribonucleotide residues in the ODN **17**/cDNA and ODN **18**/cDNA duplexes, including the T${}^{4{}^{'}\text{-}\mathrm{SCF}{}_{3}}$ residue in the former, exhibited *South‐type* sugar conformations.

Melting studies based on UV absorbance revealed that incorporation of T${}^{4{}^{'}\text{-}\mathrm{SCF}{}_{3}}$ decreased the melting temperatures (*T*_m_) of ODN/cDNA duplexes by approximately 2.0 °C/modification (Table 2 and Figure S15). In DNA/cRNA hybrids, which exhibit wider minor grooves than DNA/DNA duplexes, T${}^{4{}^{'}\text{-}\mathrm{SCF}{}_{3}}$ modification was less thermodynamically destabilizing (approximately −1.0 °C/modification). Detailed analysis of thermodynamic parameters showed that T${}^{4{}^{'}\text{-}\mathrm{SCF}{}_{3}}$ modification in dsDNA had an entropic stabilization effect. However, this effect was compensated for by an enthalpic destabilization effect, resulting in an overall loss of free energy of stability. In contrast, the T${}^{4{}^{'}\text{-}\mathrm{SCF}{}_{3}}$ modification enthalpically stabilized the DNA/cRNA hybrid. Interactions between 4′‐SCF_3_ and nucleobases in the minor groove seemed to stabilize stacking interactions within the hybrid, whereas in DNA duplexes, which have a narrower minor groove, this effect was superseded by a destabilization effect due to steric hindrance.

**Table 2:** Melting temperatures and thermodynamic parameters of T${}^{4{}^{'}\text{-}\mathrm{SCF}{}_{3}}$ ‐modified duplexes.^[a]^

Duplex	*T*_m_ [°C]	Δ*T*_m_	Δ*T*_m_/mod.	Δ*G* ^o^ [kcal mol^−1^	ΔΔ*G* ^o^ [kcal mol^−1^]	Δ*H* ^o^ [kcal mol^−1^]	Δ*S* ^o^ [cal mol^−1^ K^−1^]	*T*Δ*S* ^o^ [kcal mol^−1^]
ODN **13**/cDNA	44.8	–	–	−16.8	–	−136.4	−401.4	−119.6
ODN **14**/cDNA	42.7	−1.9	−1.9	−15.8	1.0	−130.7	−385.7	−114.9
ODN **15**/cDNA	37.5	−7.3	−2.4	−13.5	3.3	−129.6	−389.5	−116.1
ODN **16**/cDNA	36.2	−8.6	−1.7	−12.7	4.1	−121.6	−365.4	−108.9
ODN **13**/cRNA	43.4	–	–	−14.5	–	−108.3	−314.9	−93.8
ODN **14**/cRNA	42.8	−0.6	−0.6	−14.1	0.4	−108.7	−317.5	−94.6
ODN **15**/cRNA	40.3	−3.1	−1.0	−13.7	0.8	−115.6	−341.9	−101.9
ODN **16**/cRNA	38.9	−5.5	−1.1	−13.5	1.0	−118.4	−352.1	−104.9

[a] Buffer: 10 mM Na‐phosphate, 100 mM NaCl, 0.1 mM EDTA, pH 7.0. Values of Δ*H* ^o^ and *Δ*S^o^ were obtained by fitting the melting curve with linear sloping baselines using a nonlinear least‐squares software program.^[43]^

### Sensitivity of 4′‐SCF_3_ as a ^19^F NMR Probe for Monitoring Interactions in the Minor Groove of DNA Duplexes

**Figure 3:**
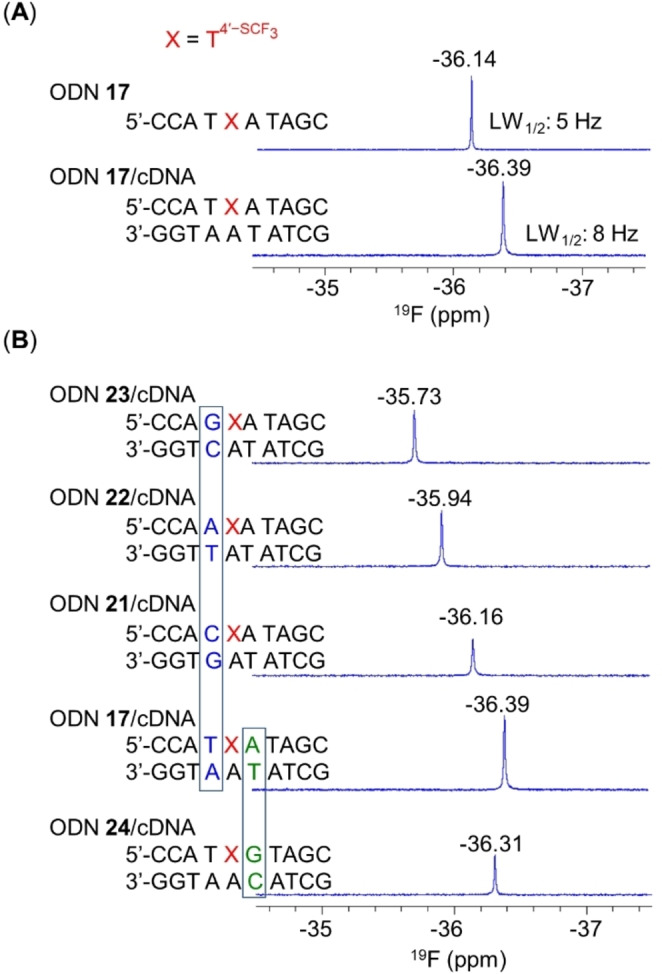
^19^F NMR spectra of T${}^{4{}^{'}\text{-}\mathrm{SCF}{}_{3}}$ ‐modified ODNs. A) Comparison of ^19^F NMR signal of T${}^{4{}^{'}\text{-}\mathrm{SCF}{}_{3}}$ ‐modified ODN **17** with that of an ODN **17**/cDNA duplex. B) ^19^F NMR spectra of various T${}^{4{}^{'}\text{-}\mathrm{SCF}{}_{3}}$ ‐modified DNA duplexes. Sample conditions: 0.1 mM ODN per strand, 10 mM Na‐phosphate, 10 % D_2_O (v/v), 278 K, 564.7 MHz.

In the ^19^F NMR spectrum of T${}^{4{}^{'}\text{-}\mathrm{SCF}{}_{3}}$ ‐modified ODN **17**, a sharp signal was observed at δ −36.14 ppm with a line‐width at half height (LW_1/2_) of 5 Hz (Figure 3A). Hybridization of ODN **17** with its cDNA resulted in an upfield shift of this signal to −36.39 ppm and an increase in the LW_1/2_ to 8 Hz. These changes, coupled with distinct relaxation properties, indicate that T${}^{4{}^{'}\text{-}\mathrm{SCF}{}_{3}}$ is a sensitive probe for distinguishing between various DNA secondary structures (Figure S16).

Moreover, because of the orientation of 4′‐SCF_3_ in the duplex minor groove, in particular the group's proximity to the 5′ flanking base pair, we assumed that the ^19^F NMR signal of 4′‐SCF_3_ might be sensitive to details of the 5′ upstream sequence. To examine this hypothesis, we prepared three DNA duplexes (ODN **21**/cDNA, ODN **22**/cDNA, and ODN **23**/cDNA; Figures 3B and S17) with sequences similar to that of ODN **17**/cDNA but different base pairs flanking the T${}^{4{}^{'}\text{-}\mathrm{SCF}{}_{3}}$ residue at the 5′ end. The ^19^F chemical shifts of the 4′‐SCF_3_ group in these three duplexes differed from that of ODN **17**/cDNA (δ −36.39) by 0.23–0.66 ppm. These results demonstrate the sensitivity of the T${}^{4{}^{'}\text{-}\mathrm{SCF}{}_{3}}$ ^19^F NMR chemical shift to changes in the 5′ flanking base pair. Notably, the structures reported herein show that duplex features were not altered by modification with T${}^{4{}^{'}\text{-}\mathrm{SCF}{}_{3}}$ and that the 4′‐SCF_3_ group oriented proximal to the 5′ flanking base pair in the minor groove. In contrast, changes in the base pair flanking the modification site at the 3′ end, such as in duplex ODN **24**/cDNA, led to only a minor chemical shift variation (Δδ=0.08 ppm) compared with that for ODN **17**/cDNA. Taken together, our results suggest that T${}^{4{}^{'}\text{-}\mathrm{SCF}{}_{3}}$ is a valuable new probe for detecting single‐nucleotide polymorphisms via ^19^F NMR spectroscopy.

### Application of T${}^{4{}^{'}\text{-}\mathrm{SCF}{}_{3}}$ for Monitoring DNA‐Protein Interactions in Duplex Minor Grooves

**Figure 4:**
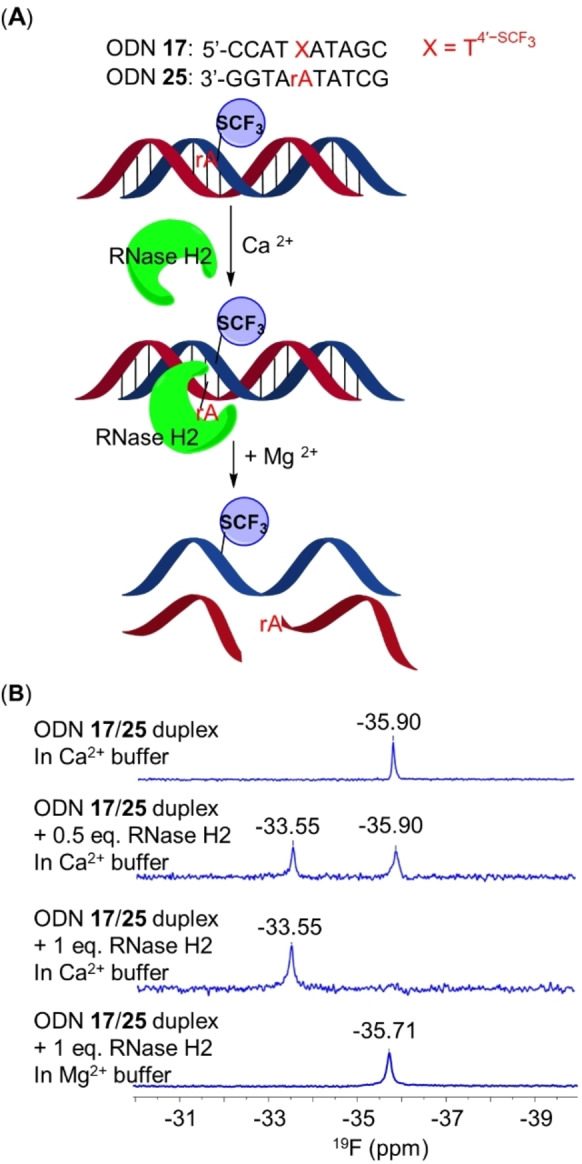
Sensitivity of ^19^F NMR chemical shifts of 4∋‐SCF_3_ to interactions between an ODN **17**/**25** duplex and RNase H2. A) Schematic representation of interactions between RNase H2 with the duplex. B) ^19^F NMR spectra of the duplex in Ca^2+^ buffer (top), in Ca^2+^ buffer containing 0.5 or 1.0 eq. of RNase H2 (middle two), and in Ca^2+^ buffer containing 1.0 eq. of RNase H2 and 50 mM MgCl_2_ (bottom). Sample conditions: 0.05 mM ODNs per strand, 10 % D_2_O (v/v), 376.5 MHz, 20 °C. Ca^2+^ buffer: 20 mM Tris‐HCl, 10 mM (NH_4_)_2_SO_4_, 10 mM KCl, 2 mM CaCl_2_, 0.1 % (w/w) Triton X‐100, pH 7.5.

Given that in dsDNA, the 4′‐SCF_3_ group located in the minor groove and did not obviously disturb the overall duplex, T${}^{4{}^{'}\text{-}\mathrm{SCF}{}_{3}}$ may also constitute a novel ^19^F NMR probe for monitoring DNA‐protein interactions, especially for minor groove‐binding proteins such as RNase H2. This ribonucleotide excision repair enzyme cleaves the 5′‐phosphate of a ribonucleotide embedded in dsDNA in the presence of Mg^2+^ ions. Notably, in the presence of Ca^2+^ ions, RNase H2 binds to dsDNA without catalyzing cleavage.^[32]^ We thus designed a DNA duplex (ODN **17**/**25**, Figure 4A) containing one T${}^{4{}^{'}\text{-}\mathrm{SCF}{}_{3}}$ /rA base pair to explore the use of T${}^{4{}^{'}\text{-}\mathrm{SCF}{}_{3}}$ for studying DNA‐protein interactions.

The ODN **17**/**25** duplex exhibited a ^19^F NMR signal at δ −35.90 ppm in 2 mM CaCl_2_ solution (Figure 4B). Adding 0.5 eq. of RNase H2 to the solution resulted in the appearance of a new signal at δ −33.55 ppm. Further additions of RNase H2 up to a total of 1.0 eq. enhanced the intensity of this new signal and led to the complete disappearance of the signal at δ −35.90 ppm. The new signal corresponds to a ODN **17**/**25** duplex‐RNase H2 complex. The binding of RNase H2 in the minor groove appeared to greatly change the microenvironment around the 4′‐SCF_3_ and thus led to a rather large chemical shift perturbation (Δδ=2.35 ppm). When Mg^2+^ ions (50 mM) were added to the ODN **17**/**25** duplex‐RNase H2 complex, the signal at δ −33.55 ppm disappeared, and a new signal at δ −35.71 ppm was observed. This new signal corresponds to single‐stranded ODN **17**, suggesting that the complementary strand, ODN **25**, had been fully cleaved (Figures S18 and S19). Kinetic studies revealed that cleavage of ODN **17**/**25** by RNase H2 was only slightly slower than cleavage of the native counterpart (ODN **18**/**25**, Figure S18). These results once again confirm that 4′‐SCF_3_ did not obviously disturb dsDNA or prevent proteins from binding to and processing dsDNA in the minor groove. Therefore, T${}^{4{}^{'}\text{-}\mathrm{SCF}{}_{3}}$ can serve as a novel ^19^F NMR probe for studying DNA‐protein interactions.

### Application of T${}^{4{}^{'}\text{-}\mathrm{SCF}{}_{3}}$ for Discriminating between Different G4 Topologies

Guanine‐rich DNA regions have been shown to adopt G‐quadruplex (G4) structures with various topologies. The polymorphism of G‐rich DNA is notoriously difficult to study by means of standard NMR spectroscopy techniques owing to severe peak overlap and limited resolution. ODN **26**, which is related to the *c‐myc* gene promoter, adopts a unimolecular parallel structure (cMYC‐G4).[^44^, ^45^] In contrast, ODN **28**, which originates from the human telomere region, forms an intramolecular antiparallel G4 topology (hTEL‐G4) in solutions containing Na^+^, but it adopts several different G4 topologies in the presence of K^+^.[^46^, ^47^, ^48^, ^49^, ^50^] To determine whether T${}^{4{}^{'}\text{-}\mathrm{SCF}{}_{3}}$ could be used to distinguish between various G4 topologies by means of ^19^F NMR spectroscopy, we introduced T${}^{4{}^{'}\text{-}\mathrm{SCF}{}_{3}}$ at the T10 position of cMYC‐G4 (ODN **27**) and at the T5 position of hTEL‐G4 (ODN **29**).

**Figure 5:**
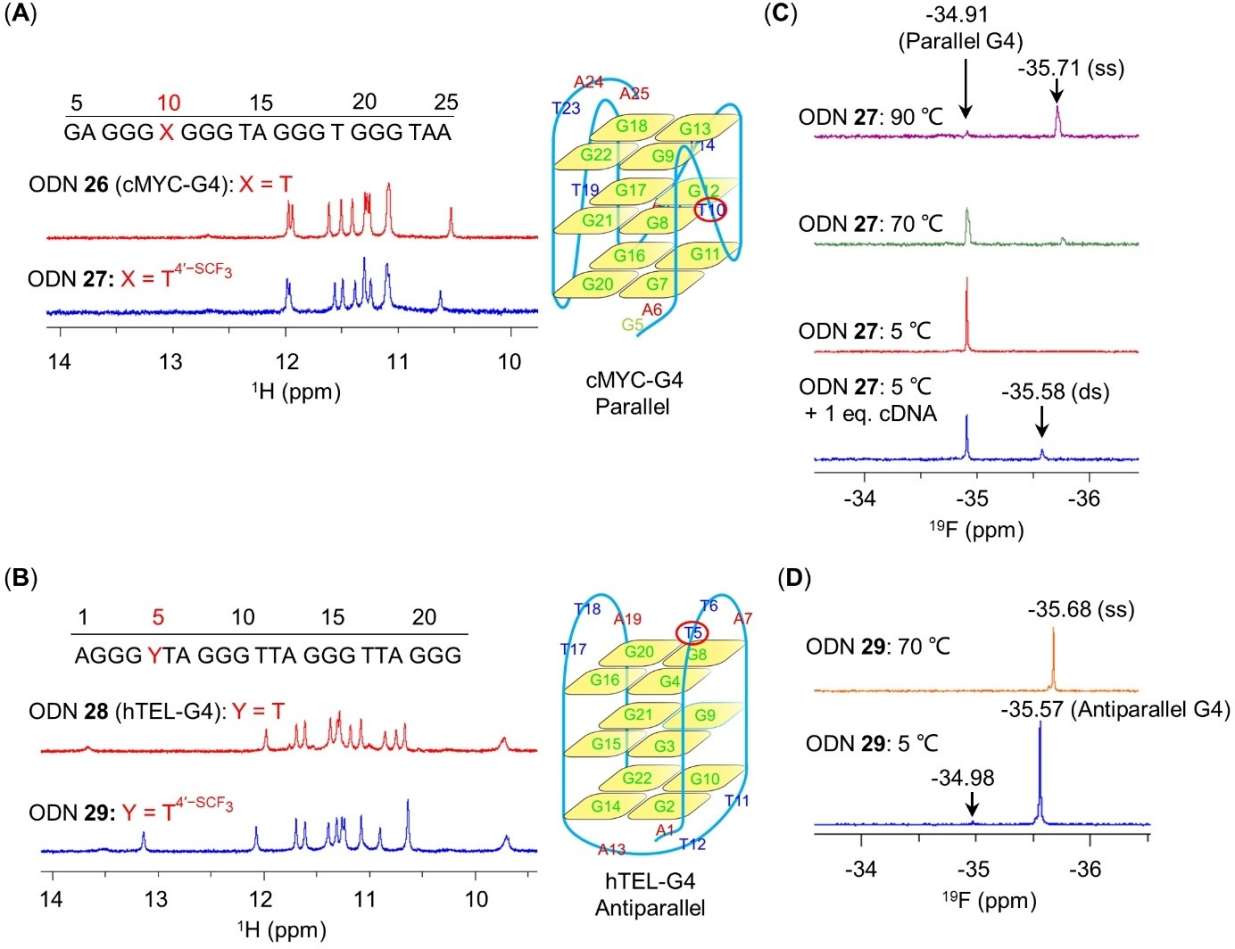
Use of T${}^{4{}^{'}\text{-}\mathrm{SCF}{}_{3}}$ modification for ^19^F NMR analysis of G4‐forming ODNs. A, B) Imino region of the ^1^H NMR spectra of parent and T${}^{4{}^{'}\text{-}\mathrm{SCF}{}_{3}}$ ‐modified ODNs that form cMYC‐G4 and hTEL‐G4 topologies. Corresponding schematic representations of the parallel and antiparallel G4 topologies are shown on the right.[^44^, ^46^] C) ^19^F NMR spectra of T${}^{4{}^{'}\text{-}\mathrm{SCF}{}_{3}}$ ‐modified ODN **27** (cMYC‐G4). Sample conditions: 0.2 mM ODN **27** in 25 mM K‐phosphate, pH 7.0, 70 mM KCl, 10 % D_2_O (v/v), 564.7 MHz. D) ^19^F NMR spectra of T${}^{4{}^{'}\text{-}\mathrm{SCF}{}_{3}}$ ‐modified ODN **29** (hTEL‐G4). Sample conditions: 0.2 mM ODN **29** in 20 mM Na‐phosphate, pH 7.0, 80 mM NaCl, 10 % D_2_O (v/v), 564.7 MHz.

^1^H NMR spectroscopy revealed that in a K^+^‐containing solution at 5 °C, the imino region of ODN **27** was almost identical to that of the unmodified ODN **26** (cMYC‐G4, Figure 5A). The twelve imino ^1^H NMR signals indicated that both ODNs formed a parallel G4 topology with three‐quarters. In contrast, in Na^+^‐containing solution at 5 °C, the T${}^{4{}^{'}\text{-}\mathrm{SCF}{}_{3}}$ ‐modified ODN **29** exhibited a slightly different ^1^H NMR spectrum than that of native ODN **28** (hTEL‐G4, Figure 5B). Moreover, an additional ^1^H NMR imino signal at δ 13.1 ppm was observed for ODN **29** and was assigned to T6 (Figure S20), suggesting that the presence of T${}^{4{}^{'}\text{-}\mathrm{SCF}{}_{3}}$ at the T5 position stabilized the antiparallel G4 topology (hTEL‐G4) by promoting the formation of an additional hydrogen bond. More‐detailed NMR‐based structural analysis revealed that ODN **28** and ODN **29** adopted very similar antiparallel G4 structures in Na^+^‐containing solution (Figure S21). In addition, the formation of an antiparallel G4 topology by T${}^{4{}^{'}\text{-}\mathrm{SCF}{}_{3}}$ ‐modified ODN **29** was confirmed by melting experiments using both circular dichroism and UV spectroscopies (Figure S22). The results showed that the T${}^{4{}^{'}\text{-}\mathrm{SCF}{}_{3}}$ modification in ODN **29** caused a 2–3 °C destabilization relative to ODN **28**. Collectively, these results suggest that introduction of a T${}^{4{}^{'}\text{-}\mathrm{SCF}{}_{3}}$ modification to the loop residues expected to connect G‐rich tracts in the G4 did not hamper G4 formation, and thus that this modification can be used to distinguish various G4 topologies by means of ^19^F NMR spectroscopy.

The parallel G4 topology formed by ODN **27** (in K^+^‐containing solution at 5 °C) and the antiparallel G4 topology formed by ODN **29** (in Na^+^‐containing solution at 5 °C) exhibited major ^19^F NMR signals at δ −34.91 and −35.57 ppm, respectively (Figure 5C, D). Increasing the temperature resulted in a gradual unfolding of both G4 s into single‐stranded forms (Figure S23). The ^19^F NMR chemical shifts of T${}^{4{}^{'}\text{-}\mathrm{SCF}{}_{3}}$ in single‐stranded ODN **27** and ODN **29** were very similar (δ −35.71 and −35.68 ppm, respectively) and were readily distinguishable from signals characteristic of G4 structures. Addition of 1 equiv of cDNA to the K^+^‐containing solution of ODN **27** at 5 °C drove approximately 20 % of the parallel G4 topology into duplex form, which exhibited a ^19^F NMR signal at δ −35.58 ppm (Figures 5C and S24). In K^+^‐containing solution at 5 °C, the ^19^F NMR spectrum of ODN **29** showed two well‐separated signals at δ −35.41 and −35.70 ppm (Figure S25), which correspond to two hybrid G4 topologies.^[19]^ Additionally, a weak signal at δ −34.98 ppm was observed for ODN **29** in both Na^+^‐ and K^+^‐containing solutions (Figures 5D and S25). The ^19^F NMR chemical shift of this signal almost perfectly matches the one observed for ODN **27**, indicating that the former can be attributed to a parallel G4 topology.^[51]^ Integration of the ^19^F NMR signals revealed that the parallel G4 topology represented only approximately 3 % of total species for ODN **29** (Figures 5D and S25). Of note, this minor species was not detectable by ^1^H NMR (Figure 5B). Taken together, these results indicate that introduction of T${}^{4{}^{'}\text{-}\mathrm{SCF}{}_{3}}$ into G4‐forming sequences is a promising tool for quantitatively distinguishing their diverse topologies in solution by means of ^19^F NMR. T${}^{4{}^{'}\text{-}\mathrm{SCF}{}_{3}}$ thus represents a highly potent ^19^F NMR probe for characterizing DNA structure and dynamics.

## Conclusion

In this work, we synthesized T${}^{4{}^{'}\text{-}\mathrm{SCF}{}_{3}}$ phosphoramidite and incorporated it into DNA strands by using standard solid‐phase DNA synthesis. The 4′‐SCF_3_ modification had limited stereoelectronic effects on the deoxyribose pucker, endowing the modified DNA with structural adaptability. For example, the sugar in T${}^{4{}^{'}\text{-}\mathrm{SCF}{}_{3}}$ preferentially adopted a *North‐type* pucker in single‐stranded DNA but adopted the same *South‐type* conformation as the native thymidine in dsDNA. In addition, 4′‐SCF_3_ exhibited a rather flexible orientation in the minor groove of DNA duplexes and was well accommodated by various higher order DNA structures.

The three magnetically equivalent fluorine atoms in 4′‐SCF_3_‐DNA constitute an isolated spin system and showed high ^19^F NMR sensitivity, with the chemical shift being clearly susceptible to the surrounding environment. The high structural adaptability and high ^19^F NMR sensitivity of T${}^{4{}^{'}\text{-}\mathrm{SCF}{}_{3}}$ make it a valuable probe for elucidating DNA structure and function. We demonstrated that the ^19^F NMR chemical shift of T${}^{4{}^{'}\text{-}\mathrm{SCF}{}_{3}}$ in dsDNA clearly depended on the flanking base pair at the 5′ end but not on the flanking base pair at the 3′ end; this phenomenon is potentially useful for detecting single‐nucleotide polymorphisms. The 4′‐SCF_3_ modification did not interfere with the binding of RNase H2 to the minor groove of dsDNA, allowing quantitative monitoring of DNA‐protein interactions by means of ^19^F NMR. In addition, this novel label represents an invaluable ^19^F NMR probe for distinguishing various G4 topologies.

Taken together, the unique structural and biophysical properties of 4′‐SCF_3_ make it a powerful probe for characterizing DNA structure and function. We are optimistic that 4′‐SCF_3_‐modified DNA will attract considerable attention and find a wide range of practical applications.

## Conflict of interest

The authors declare no conflict of interest.

1

## Supporting information

As a service to our authors and readers, this journal provides supporting information supplied by the authors. Such materials are peer reviewed and may be re‐organized for online delivery, but are not copy‐edited or typeset. Technical support issues arising from supporting information (other than missing files) should be addressed to the authors.

Supporting Information

## Data Availability

The coordinates for structures of 4′‐SCF3‐modified DNA duplex and the unmodified counterpart have been deposited in the Protein Data Bank (accession numbers: 7W0V and 7QA9)
