# Supplementary material for: 4′‐SCF3‐Labeling Constitutes a Sensitive 19F NMR Probe for Characterization of Interactions in the Minor Groove of DNA
Source: Angew Chem Int Ed Engl. 2022 Oct 19;61(47):e202201848. doi: 10.1002/anie.202201848 (PMC9828712; doi:10.1002/anie.202201848)
Supplement: Supplementary file 1 — Supporting Information [file ANIE-61-0-s001.pdf]

Angewandte Chemie
Eine Zeitschrift der Gesellschaft Deutscher Chemiker

Supporting Information

4′-SCF_3-Labeling Constitutes a Sensitive ^{19}F NMR Probe for Characterization of Interactions in the Minor Groove of DNA

Q. Li, M. Trajkovski, C. Fan, J. Chen, Y. Zhou, K. Lu, H. Li, X. Su, Z. Xi, J. Plavec, C. Zhou**

Supplementary Experiments……………………………………………………….S2
Supplementary Tables…………………………………………………………….S9
Supplementary Figures ……………………………………………………….....S14
Supplementary References ……………………………………………………..S61

Supplementary experiments

Material and methods

General

$AgSCF_3$ and 2-((trifluoromethyl)thio)-isoindoline-1,3-dione (N-SCF_3-phthalimide, CAS: 719-98-2) were synthesized following the published literature[1]. Besides, all substances were purchased from commercial suppliers (Inno-chem, J&K scientific, Energy-chemical, Merck/Sigma-Aldrich). All solvents were dried following standard procedure. Silica gel 60 (mesh size 0.023 - 0.04 mm) was used for column chromatography. The procedures for chemical synthesis of C4′-SCF_3-modified phosphoramidite **12** and their characterization data are available in the Supporting Information. ^{1}H, ^{13}C, ^{19}F and ^{31}P NMR spectra were recorded on a Bruker Advance 400 MHz spectrometer. Chemical shifts (^{1}H, ^{13}C) are reported referenced to the residual solvent signal. High resolution mass spectra were recorded on a Waters UPLC-Xevo-G2-XS-ESI-Q-TOF in positive ion mode.

Synthesis of 4′-SCF_3 thymidine building block.

2′-Deoxy-3′-*O*-(*tert*-Butyldimethylsilyl)-4′-trifluoromethylthio-α-L-xylofuranosyl-thymine (3).

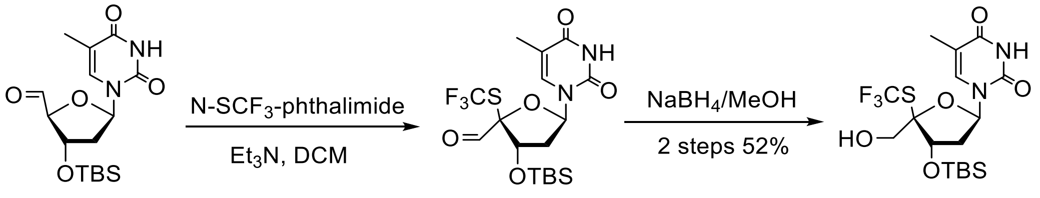

1 **2** **3**

A mixture of N-SCF_3-phthalimide (0.37 g, 1.5 mmol), Et_3N (0.42 mL, 3 mmol) in 5 mL dichloromethane (DCM) was stirred for 15 min at room temperature. **1** (0.18 g, 0.5 mmol) was added and the solution was stirred for another 5 h and then concentrated to dryness, partitioned between DCM and saturated aqueous $NaHCO_3$. Column chromatography (EtOAc/hexane = 1:3) gave 4′-trifluoromethylthiolated aldehyde **2** (156 mg, 0.3 mmol, 60%). ^{1}H NMR (400 MHz, $CDCl_3$) δ (ppm) 9.50 (s, 1H, CHO), 9.35 (s, 1H, NH), 7.37 (s, 1H, H-6, =CH), 6.84 (m, 1H, H-1′), 4.64-4.63 (m, 1H, H-3), 2.45-2.41 (m, 2H, H-2′, H-2″), 1.94 (s, 3H, CH_3), 0.86 (s, 9H, ^{t}Bu), 0.10 (s, 3H, -CH_3) , 0.09 (s, 3H, -CH_3); ^{13}C NMR (100.6 MHz, $CDCl_3$) δ (ppm) 192.8, 163.6, 150.7, 135.3, 128.6 (q, *J* = 310.85 Hz, 1C, SCF_3), 112.2, 97.9, 89.3, 79.0, 39.7, 25.5, 17.9, 12.5, -4.9, -5.3; ^{19}F NMR (376.5 MHz, $CDCl_3$) δ (ppm) -34.38 (s, 3F, SCF_3). HRMS(ESI): $C_{17}H_{26}F_3N_2O_5SSi$ $[M+H]^+$ calc. 455.1284; found 455.1223.

2 was dissolved in 50 mL MeOH and treated with $NaBH_4$ (18 mg, 0.45 mmol). After stirring for 10 min at room temperature, the mixture was evaporated and partitioned between DCM and brine. The organic layer was dried over sodium sulfate, filtered and concentrated on vacuum. The residue was purified on a silica gel column (EA:PE = 1:3) to give **3** (118 mg, 0.26 mmol 86%) as a white solid.

Compound **3**: ^{1}H NMR (400 MHz, $CDCl_3$) δ (ppm) 9.34 (s, 1H, NH), 7.37 (s, 1H, H-6, =CH), 6.58 (t, *J* = 7.2 Hz, 1H, H-1′), 4.54-4.49 (m, 1H, H-3′), 4.07-3.98 (m, 2H, H-5′, H-5″), 2.54-2.47 (m, 1H, H-2′), 2.44-2.36 (m, 1H, H-2″), 1.93 (s, 3H, CH_3), 0.92 (s, 9H, ^{t}Bu), 0.15 (s, 3H, -CH_3), 0.14 (s, 3H, -CH_3); ^{13}C NMR (100.6 MHz, $CDCl_3$) δ (ppm) 163.7, 150.4, 135.4, 129.2 (q, *J* =

309.2 Hz, 1C), 111.9, 101.2, 87.5, 77.5, 63.6, 39.7, 25.6, 18.0, 12.6, -4.9, -5.3; ^{19}F NMR (376.5 MHz, $CDCl_3$) δ (ppm) -34.57 (s, 3F, SC<u>F</u>3). HRMS(ESI): $C_{17}H_{28}F_3N_2O_5SSi$ $[M+H]^+$ calc. 457.1440; found 457.1450.

5′-Aldehyde-2′-deoxy-3′-*O*-(*tert*-Butyldimethylsilyl)-β-D-xylofuranosyl-thymine (5).

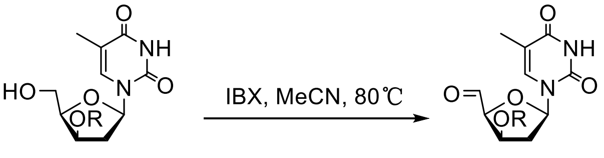

R = TBS R = TBS

4 **5**

To the solution of **4** (8.68 g, 24.30 mmol) in 200 mL anhydrous acetonitrile was added IBX (8.2 g, 29.16 mmol). After refluxing and stirring for 1 h at 80°C, the reaction mixture was filtered through a Celite pad. The resulting filtrate was evaporated and then dissolved in EtOAc (100 mL), washed with saturated aqueous $NaHCO_3$ (100 mL). The organic layer was dried over sodium sulfate, filtered and concentrated on vacumm. The residue was purified on a silica gel column (EtOAc/hexane = 2:3) to give **5** (7.80 g, 22 mmol, 90%) as a white solid.

2′-Deoxy-3′-*O*-(*tert*-Butyldimethylsilyl)-4′-trifluoromethylthio-β-D-xylofuranosyl-thymine (7).

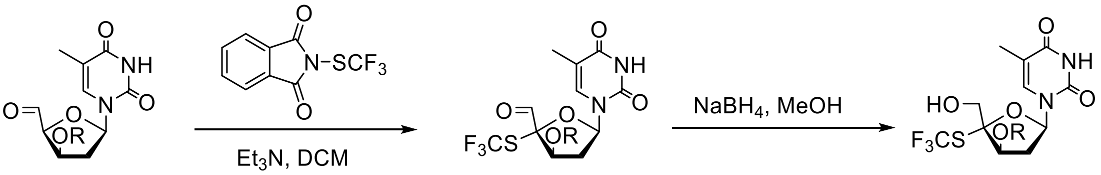

R = TBS R = TBS R = TBS

5 **6** **7**

A mixture of N-SCF_3-phthalimide (5.39 g, 21.80 mmol), Et_3N (6 mL, 43.56 mmol) in 200 mL dichloromethane (DCM) was stirred for 15 min at room temperature. **5** (2.57 g, 7.20 mmol) was added and the solution was stirred for another 6 h and then concentrated to dryness, partitioned between DCM and saturated aqueous $NaHCO_3$. Column chromatography (EtOAc/hexane = 1:3) gave 4′-trifluoromethylthiolated aldehyde **6** (2.1 g, 4.7 mmol, 65%). ^{19}F NMR (376.5 MHz, $CDCl_3$) δ (ppm) -35.99; HRMS(ESI): $C_{17}H_{26}F_3N_2O_5SSi$ $[M+H]^+$ calc. 455.1284; found 455.1267.

6 was dissolved in 50 mL MeOH and treated with $NaBH_4$ (0.26 g, 7.05 mmol). After stirring for 10 min at rt, the mixture was evaporated and partitioned between DCM and brine. The organic layer was dried over sodium sulfate, filtered and concentrated on vacuum. The residue was purified on a silica gel column (EA:PE = 1:3) to give **7** (1.83 g, 4.0 mmol, 85%) as a white solid. ^1H NMR (400 MHz, $CDCl_3$) δ (ppm) 9.55 (s, 1H, N<u>H</u>), 7.59 (s, 1H, H-6, =C<u>H</u>), 6.57-6.50 (m, 1H, H-1′), 4.55-4.51 (m, 1H, H-3′), 4.16 (d, *J* = 12.3 Hz, 1H, H-5′), 4.07 (d, *J* = 12.2 Hz, 1H, H-5″), 3.07-2.96 (m, 1H, H-2′), 2.58 (br, 1H, -OH), 2.00-1.93 (m, 1H, H-2″), 1.91 (s, 3H, CH_3), 0.91 (s, 9H, ^tBu), 0.16 (s, 3H, -C<u>H</u>3), 0.12 (s, 3H, -C<u>H</u>3); ^{13}C NMR (100.6 MHz, $CDCl_3$) δ (ppm) 163.7, 150.4, 135.5, 129.1 (q, *J* = 309.0 Hz, 1C), 111.8, 101.1, 84.6, 77.0, 63.5, 40.6, 25.5, 17.9, 12.5, -5.1, -5.3; ^{19}F NMR (376.5 MHz, $CDCl_3$) δ (ppm) -35.34; HRMS(ESI): $C_{17}H_{28}F_3N_2O_5SSi$

[M+H]+ calc. 457.1440; found 457.1450.

2′-Deoxy-5′-*O*-(4,4′-dimethoxytrityl)-3′-O-(*tert*-butyldimethylsilyl)-4′-trifluoromethylthio-β-D-xylofuranosyl-thymine (8).

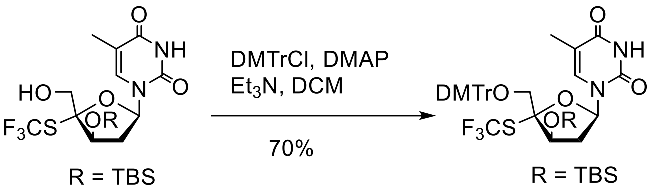

7 **8**

To a mixture of **7** (1.00 g, 2.2 mmol), Et_3N (1.2 mL, 8.8 mmol) and 4-(dimethylamino) pyridine (DMAP, 0.54g, 4.4 mmol) in 50 mL dichloromethane (DCM) was added 4,4′-dimethoxytrityl chloride (DMTrCl, 2.2 g, 6.6 mol) in three portions over a period of 6 hours. The solution was stirred for 24 h and then quenched by MeOH (1 mL). The solvent was evaporated and resulting residue was diluted with DCM, then washed with saturated aqueous NaCl. The organic layer was dried over sodium sulfate, filtered and concentrated on vacuum. The residue was purified on a silica gel column (MeOH/DCM = 0.5% (v/v), with addition of 0.5% (v/v) Et_3N) to give **8** (1.17 g, 1.54 mmol, 70%) as a white foam. ^1H NMR (400 MHz, $CDCl_3$) δ (ppm) 9.44 (s, 1H, N<u>H</u>), 7.56-7.07 (s, 10H, H-6, Ar-H), 6.85-6.81 (m, 4H, Ar-H), 6.54 (dd, *J* = 7.3, 4.2 Hz, 1H, H-1′), 4.37-4.31 (m, 1H, H-3′), 3.84 (d, *J* = 10.6 Hz, 1H, H-5′), 3.79 (s, 6H, OCH_3), 3.47 (d, *J* = 10.6 Hz, 1H, H-5″), 3.11-3.03 (m, 1H, H-2′), 2.10-2.03 (m, 1H, H-2″), 1.60 (s, 3H, CH_3), 0.75 (s, 9H, ^{t}Bu), 0.00 (s, 3H, -C<u>H</u>3), -0.03 (s, 3H, -C<u>H</u>3); ^{13}C NMR (100.6 MHz, $CDCl_3$) δ (ppm) 163.8, 158.7, 150.4, 149.7, 144.1, 136.1, 136.0, 135.5, 135.2, 130.4, 130.3, 129.3 (q, *J* = 325.0 Hz, 1C), 128.5, 127.9, 127.1, 113.1, 111.2, 100.4, 87.1, 85.0, 65.1, 55.2, 40.2, 25.5, 17.8, 12.2, -5.2, -5.4; ^{19}F NMR (376.5 MHz, $CDCl_3$) δ (ppm) -35.96; HRMS(ESI): $C_{38}H_{45}F_3N_2NaO_7SSi$ [M+H]+ calc. 781.2567; found 781.2589.

2′-Deoxy-5′-*O*-(4,4′-dimethoxytrityl)-4′-trifluoromethylthio-β-D-xylose-thymine (9).

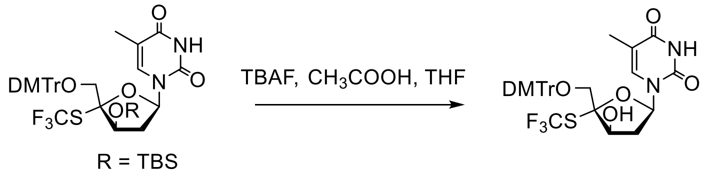

8 **9**

Compound **8** (300 mg, 0.4 mmol) was dissolved in 5 mL tetrahydrofuran (THF), to which a mixture of tetra-*n*-butylammonium fluoride (1N in THF, 0.42 mL, 0.42 mmol) and acetic acid (69 μL, 1.2 mmol) in 5 mL THF was added. The solution was stirred for 3 h at rt and dilute with 40 mL EtOAc, washed with saturated aqueous $NaHCO_3$, brine. The organic layer was dried over sodium sulfate, filtered and concentrated on vacuum. The residue was purified on a silica gel column (EtOAc/PE = 1:1) to give **9** (231 mg, 0.36 mmol, 90%) as a white foam. ^1H NMR (400 MHz, $CDCl_3$) δ (ppm) 7.50-7.20 (m, 10H, H-6, Ar-H), 6.88-6.84 (m, 4H, Ar-H), 6.54 (d, *J* = 6.4 Hz, 1H, H-1′), 4.38 (d, *J* = 5.6 Hz, 1H, H-3′), 3.95 (d, *J* = 9.8 Hz, 1H, H-5′), 3.79 (s, 6H, OCH_3), 3.53 (d, *J* = 9.8 Hz, 1H, H-5″), 3.07-2.97 (m, 1H, H-2′), 2.07-1.99 (m, 1H, H-2″), 1.75 (s, 3H,

CH_3); ^{13}C NMR (100.6 MHz, $CDCl_3$) δ (ppm) 164.2, 158.9, 150.7, 143.8, 136.6, 135.0, 134.1, 130.0, 129.9, 129.4 (q, J = 320.9 Hz), 128.1, 127.9, 127.3, 124.9, 113.5, 111.7, 99.9, 87.4, 86.3, 76.1, 64.1, 55.2, 38.2, 12.4; ^{19}F NMR (376.5 MHz, $CDCl_3$) δ (ppm) -40.51. HRMS(ESI): $C_{32}H_{31}F_3N_2NaO_7S$ $[M+Na]^+$ calc. 667.1702; found 667.1691.

4′-Trifluoromethylthio-thymidine (10).

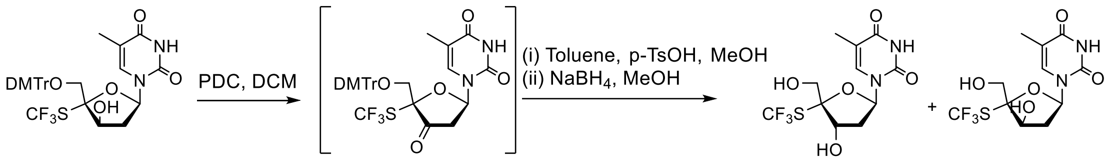

9 **10** **10'**

Pyridinium dichromate (PDC, 465 mg, 1.24 mmol) and 500 mg 3 Å molecular sieve was dispersed in 5 mL DCM, and compound **9** (400 mg, 0.62 mmol) was added. After stirring for 3 h at rt, the reaction mixture was filtered through a Celite pad and the filtrate was evaporated. The resulting residue was dissolved in 40 mL toluene, and *p*-toluenesulfonic acid (80 mg, w/v = 2%) and 1 mL MeOH was added. After stirring for 1.5 h at rt, another portion of $NaBH_4$ (45 mg, 1.2 mmol) was added and stirring continued for 10 min. Acetic acid was added to tune the pH. The residue was concentrated on vacuum and purified on a silica gel column (4% [v/v] MeOH in DCM) to give **10** (97 mg, 0.29 mmol, 46%) and **10′** (9 mg, 0.025 mmol, 4%) as a white foam.

Compound **10**: ^1H NMR (400 MHz, CD_3OD) δ (ppm) 7.56 (s, 1H, H-6), 6.38 (dd, J = 8.3, 3.5 Hz, 1H, H-1′), 4.94 (t, J = 8.5 Hz, 1H, H-3′), 4.16 (d, J = 12.5 Hz, 1H, H-5′), 3.85 (d, J = 12.5 Hz, 1H, H-5″), 2.54 (dt, J = 14.0, 8.5 Hz, 1H, H-2″), 2.39 (ddd, J = 14.0, 8.6, 3.6 Hz, 1H, H-2′), 1.83 (s, 3H, CH_3); ^{13}C NMR (100.6 MHz, CD_3OD) δ (ppm) 166.2, 150.0, 138.1, 132.3 (q, J = 306.8 Hz), 112.2, 103.5, 85.5, 71.7, 64.7, 38.4, 12.5; ^{19}F NMR (376.5 MHz, D_2O) δ (ppm) -35.21; HRMS(ESI): $C_{11}H_{14}F_3N_2O_5S$ $[M+H]^+$ calc. 343.0576; found 343.0620.

Compound **10′**: ^1H NMR (400 MHz, CD_3OD) δ (ppm) 7.79 (s, 1H, H-6), 6.43 (dd, J = 8.5, 3.8 Hz, 1H, H-1′), 4.26 (d, J = 5.6 Hz, 1H, H-3′), 4.05 (d, J = 11.9 Hz, 1H, H-5′), 3.92 (d, J = 11.9 Hz, 1H, H-5″), 2.97-2.87 (m, 1H, H-2′), 2.03-1.96 (m, 1H, H-2″), 1.79 (s, 3H, CH_3); ^{19}F NMR (376 MHz, D_2O) δ (ppm) -36.10. HRMS(ESI): $C_{11}H_{14}F_3N_2O_5S$ $[M+H]^+$ calc. 343.0576; found 343.0587.

5′-*O*-(4,4′-Dimethoxytrityl)-4′-trifluoromethylthio-thymidine (11).

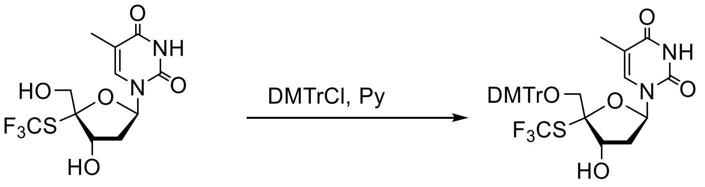

10 **11**

Compound **10** (89 mg, 0.26 mmol) was co-evaporated dry pyridine (Py) three times and finally dissolved in 10 mL dry pyridine, to which 4,4′-dimethoxytrityl chloride (DMTrCl, 114 mg, 0.34 mol) was added. The solution was stirred for 24 h and then quenched by MeOH (1 mL). The solvent was evaporated the residue was purified on a silica gel column (MeOH/DCM = 4% (v/v), with addition of 0.5% (v/v) Et_3N) to give **11** (94 mg, 0.15 mmol 56%) as a white foam. ^1H

NMR (400 MHz, $CDCl_3$) δ (ppm) 7.45-7.21 (m, 10H, H-6, Ar-H), 6.85 (s, 2H, Ar-H), 6.83 (s, 2H, Ar-H), 6.44-6.37 (m, 1H, H-1′), 4.95 (t, *J* = 7.9 Hz, 1H, H-3′), 3.94 (d, *J* = 10.5 Hz, 1H, H-5′), 3.77 (s, 6H, OCH_3), 3.72 (d, *J* = 12.5 Hz, 1H, H-5″), 2.72-2.60 (m, 1H, H-2″), 2.47-2.36 (m, 1H, H-2′), 1.48 (s, 3H, CH_3); ^{13}C NMR (100.6 MHz, $CDCl_3$) δ (ppm) 164.0, 158.9, 150.1, 140.1, 135.8, 130.1, 130.2 (q, *J* = 308.8 Hz), 128.2, 127.3, 113.4, 111.8, 100.6, 87.6, 84.8, 72.9, 65.7, 55.3, 37.9, 11.9; ^{19}F NMR (376.5 MHz, CD_3Cl) δ (ppm) -35.05. HRMS(ESI): $C_{32}H_{31}F_3N_2NaO_7S$ $[M+Na]^+$ calc. 667.1702; found 667.1691.

5′-*O*-(4,4′-Dimethoxytriry)-4′-trifluoromethylthio-thymidine, 3′-*O*-[2-cyanoethyl-*N,N*-diisopropyl-phosphoramidite] (12).

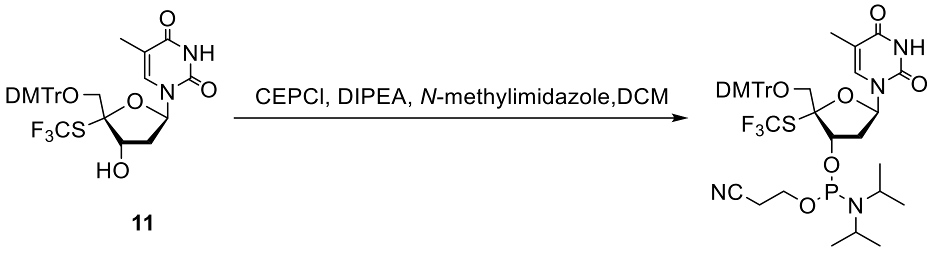

11

12

11 (90 mg, 0.14 mmol) was dissolved in 10 mL anhydrous DCM. The *N,N*-diisopropylethylamine (185 μL, 1.12 mmol), *N*-methylimidazole (NMI, 45 μL, 0.56 mmol) and 2-cyanoethyl-N,N-diisopropylchlorophosphoramidite (CEPCl, 93μL, 0.42 mmol) was added in steps. The solution was stirred for 1 h. The solvent was evaporated the residue was purified on a silica gel column (EtOAc/PE = 3:2 (v/v), with addition of 0.5% (v/v) Et_3N) to give **12** (71 mg, 0.084 mmol 60%) as a white foam. ^{31}P NMR (162 MHz, $CDCl_3$) δ (ppm) 150.86 (s, 1P), 150.78 (s, 0.85P); ^{19}F NMR (376.5 MHz, $CDCl_3$) δ (ppm) -35.08, -35.15. $C_{41}H_{48}F_3N_4NaO_8PS$ $[M+Na]^+$ calc. 867.2780; found 867.2822.

Oligonucleotide (ODN) synthesis and purification

All DNA oligomers were synthesized on an Applied Biosystems Incorporated 394 oligonucleotide synthesizer using standard DNA synthesis cycle. For incorporating $T^{4'\text{-}SCF_3}$, the coupling time was extended to 10 min. After synthesis, solid supports were treated with 1 mL 28% (w/w) aqueous ammonium at room temperature for 16-60 h. The supernatant as then separated from the solid supports, and evaporated to dryness. The obtained crude products purified by denaturing PAGE (20% [w/v] polyacrylamide with 7 M urea), extracted with 0.3 M NaOAc, and desalted with Sep-Pak C18 column to give ODNs in >99% purity. ODN concentration was determined on NanoDrop 2000 by measuring absorbance at 260 nm. The integrity of these oligomers was characterized by Waters UPLC-Xevo-G2-XS-ESI-Q-TOF in negative ion mode.

Stability of $T^{4'\text{-}SCF_3}$modified oligonucleotides

Generally, 5′-FAM-labeled ODN (0.5 μM) was dissolved in DEPC water. A final concentration of 20 mm buffer was added to establish a given pH (pH 5.2 Sodium Acetate; pH 7.0 sodium phosphate; pH 10.0 borax-sodium hydroxide). Total volume is 100 μL. The mixture was incubated 24 h at 37 °C. Aliquots were taken at appropriate intervals, quenched with 10 μL

formamide, and analyzed by 20% denaturing polyacrylamide gel. The gel was visualized using a GE Typhoon Gel Imaging Scanner with excitation at 488 nm and emission at 520 nm.

UV melting measurements

UV thermal scans were performed on UV/VIS Spectrometer Lambda 35 with an optical path of 1 cm. $A_{260\ nm}$ was recorded as a function of temperature over the range from 20 to 60 ºC with the heating rate at 0.5 ºC/min. Prior to the measurements, the solution containing 2.0 μM duplex in a buffer of 10 mM sodium phosphate (pH 7.0) containing 100 mM NaCl and 0.1 mM EDTA was heated 5 min in 95 ºC, then slowly cooled to room temperature. T_m was determined as the fraction of folded duplex at 50%. In brief, the upper and lower baselines of the melting curve were manually chosen and the median was plotted as the algebraic average of the two baselines. The intercept with the absorbance axis corresponds to the T_m. Thermodynamic parameters were determined according to published method [2-3]. Assuming bimolecular equilibrium, the binding constant (Ka) is calculated. Then plot ln(Ka) as a function of the reciprocal of temperature (K): $\ln(Ka) = (-\Delta H/R)\times(1/T)+\Delta S/R$, R, used is 1.987 $cal\bullet mol^{-1}\bullet K^{-1}$.

RNase H2 digestion assay

Activity. Target 5′-FAM-labeled ODN with a ribonucleotide and complentary DNA were incubated in a buffer containing 20 mM Tris-HCl, pH 7.5, 10 mM $(NH_4)2SO_4$, 10 mM KCl, 10 mM $MgCl_2$, 0.1% (w/w) Triton X-100 at 21ºC in the presence of various amount of *Thermotoga maritima* RNase H2. Prior to the addition of the enzyme, reaction tubes with reaction components were annealed and slowly cooled to 21ºC and kept at this temperature for 10 min. Total reaction volume was 20 μL. After 30 min, the reactions were terminated by mixing with stop solution [containing 0.05 M EDTA, 0.05% (w/v) xylene cyanole, and 0.05% (w/v) bromophenol blue in 90% (v/v) formamide]. The samples were subjected to PAGE and visualized by Typhoon.

Kinetics. The DNA duplex with a single ribonucleotide incorporated were incubated in the reaction buffer mentioned above at 21 ºC. After equilibration for 10 min, 0.05 μM RNase H2 were added. Total reaction volume was 100 μL. Aliquots of 10 μL were removed after incubation. (0, 15, 30, 60, 120, 180, 240, 300, and 360 min for modified duplex; 0, 5, 10, 20, 30, 45, 60, 90, and 120 min for unmodified duplex), and the reactions were terminated by mixing with stop solution mentioned above. The samples were subjected to PAGE and visualized by Typhoon. Experiments were repeated three times and data were analyzed by Prism.

NMR studies of oligonucleotides

NMR experiments were performed on Bruker AVANCE NEO or AVANCE III HD 600 MHz NMR spectrometers equipped with 5 mm ^1H-optimized quadruple resonance cryo probe in the temperature range from 5 to 90 ºC in 9/1 H_2O/D_2O or 100% D_2O. NMR samples were prepared by dissolving DNA oligonucleotides in 10 mM sodium phosphate buffer (pH 7.0) with the final concentration in the range of 0.2 to 0.5 mM. Excitation sculpting pulse was used for solvent suppression. Proton NMR spectra were referenced to Sodium trimethylsilyl propanesulfonate (DSS). 2D NOESY spectra were recorded at mixing times between 80, 150, 200 and 400 ms, 2D ROESY at mixing time 200 ms, 2D TOCSY at mixing time 60 ms, DQF-COSY spectra were acquired in 100 % D_2O. NMR spectra were processed and analyzed using TopSpin and Sparky

(UCSF) software [4].

1D ^{19}F NMR and 2D $^{19}F/^{1}H$ HOESY spectra were recorded at a frequency of 376.5 MHz on a Bruker Avance 400 MHz NMR spectrometer equipped with a BBO automatic tuning probe using a zgfhigqn.2 pulse sequence or at a frequency of 564.7 MHz on a Bruker Avance Neo 600 MHz NMR spectrometer equipped with a 5 mm 1H-optimized quadruple resonance cryo probe. Typical experimental parameters were chosen as follows: spectral width 20 ppm, acquisition time 0.72 s, pre-scan delay 6.5 μs, receiver gain (RG) 101, number of scans 128. Prior to Fourier transformation all time domain data was processed with an exponential window function using a line broadening factor of 0.3 Hz. ^{19}F-resonances were referenced relative to external CCl_3F or trifluoro toluene.

NOE-distance restrained molecular dynamics calculations

The distances between interproton in duplex were calculated from 150 ms NOESY spectra. The average distance of total four cytosine H5-H6 (2.5 Å) was used as a reference. Three categories (strong: 1.8–3.6Å, medium: 2.6–5.0Å, and weak: 3.5–6.5Å) were used for cross-peaks based on calculated distances [5]. Based on the NOE correlations of H8/H6 with H1′, H2′ and H2″, glycosidic torsion angles for all residues were restrained to *anti* conformation. The partial charges for the modified nucleoside $T^{4'\text{-}SCF_3}$were calculated using R.E.D sever program [6]. Initial structure of duplex was created using X3DNA software [7] and AMBER 20 program [8]. Amber 20 program was used for simulated annealing (SA) calculations that were performed with random velocity. Tolerance of 0.00005 Å was chosen for the SHAKE algorithm for hydrogen atoms. After that, 10 structures with the lowest energy were subjected to energy minimization with a maximum of 100000 steps of steepest descent. DNA 2.0 software was used to determine helical parameters.

CD Spetroscopy

CD experiments were performed on Applied Photophysics Chirascan CD spectrometer over the 210−320 nm wavelength range, with the use of 0.1 cm peth-length quartz cells. The samples were prepared at 10 μM concentration in 10 mM sodium phosphate bugger (pH 7.0)

Supplementary tables

Table S1. Deoxyribose conformation analysis based on *J* -coupling constants.

	Nucleoside **10**: $T^{4'\text{-}SCF_3}$	trinucleotide **19**: $T^1T^{4'\text{-}SCF_3}A^3$			trinucleotide **20**: $T^1T^2A^3$		
		T^1	$T^{4'\text{-}SCF_3}$	A^3	T^1	T^2	A^3
$J_{H1'\text{-}H2'}$	3.50	7.69	4.65	6.54	7.91	8.70	6.99
$J_{H1'\text{-}H2''}$	8.30	6.25	8.55	6.54	6.55	6.38	6.99
$\Sigma_{H1'}$ [a]	11.80	13.94	13.20	13.08	14.46	15.08	13.99
$\Sigma_{H2'}$ [b]	26.20	28.30	27.83	27.05	25.15	29.93	27.93
$\Sigma_{H2''}$ [c]	30.80	23.27	30.50	25.16	23.56	23.56	25.25
$J_{H3'\text{-}H2'}$	8.50	6.80	8.70	6.50	n.r.	6.90	6.70
$J_{H3'\text{-}H2''}$	8.50	3.50	7.80	4.60	3.10	n.r.	4.30
%South[d]	6.4%	75%	9%	58%	73%	73%	57%
%North[e]	90%	26%	87%	39%	25%	-	40%

[a] $\Sigma_{H1'} = J_{H1'\text{-}H2'} + J_{H1'\text{-}H2''}$; [b] $\Sigma_{H2'} = J_{H1'\text{-}H2'} + J_{H2'\text{-}H2''} + J_{H3'\text{-}H2'}$; [c] $\Sigma_{H2''} = J_{H1'\text{-}H2''} + J_{H2'\text{-}H2''} + J_{H3'\text{-}H2''}$; [d]calculated based on: %South = 100×[31.5 −$\Sigma_{H2''}$]/10.9; [e] calculated based on: %North = 100×[$J_{H1'\text{-}H2''}$+$J_{H3'\text{-}H2''}$ −6.9] / 10.9; n.r. not resolved.

Table S2. NMR restraints and structural statistics for DNA duplexes.

	ODN **17**/cDNA	ODN **18**/cDNA
NOE-derived distance restraints		
Total NOE	426	489
Intranucleotide NOEs	222	291
Sequential (i, i+1)	191	183
Long-range (i, > i+1)	13	15
Torsion angle restraints	20	20
Hydrogen-bond restraints	24	24
Structural statistics		
NOE violations >0.2Å	0	0
Pairwise all (H, F, C, O, N, P) atom RMSD (Å)		
Overall	0.395	0.256
Without T5	0.341	0.230
Pairwise heavy (C, O, N, P) atom RMSD (Å)		
Overall	0.244	0.069
Without T5	0.244	0.073

Table S3. ^{1}H NMR chemical shift for ssDNA ODN **17** (data from NOESY at 298K).

Residue	H1′	H2′	H2′	H3′	H4′	H5′	H5″	H6/H8	H2/Me/H5	^{31}P
C1	6.029	2.396	2.124	4.595	4.033	3.628	3.690	7.583	5.822	-
C2	5.980	2.235	1.762	4.663	4.080	3.882	3.882	7.482	5.835	−0.966
A3	6.220	2.693	2.692	4.884	4.311	4.107	3.980	8.290	8.014	−1.120
T4	5.845	2.241	2.002	4.698	4.089	4.087	3.986	7.241	1.569	−1.317
T5	6.090	2.368	2.007	5.068	-	4.072	4.238	7.154	1.699	−2.061
A6	6.204	2.683	2.686	4.904	4.297	3.940	3.991	8.220	7.980	−1.926
T7	5.739	1.967	1.560	4.631	3.995	3.988	3.928	7.077	1.574	−1.303
A8	5.957	2.596	2.493	4.857	4.249	3.946	3.864	8.055	7.908	−1.386
G9	5.856	2.480	2.555	4.867	4.258	4.101	4.101	7.801	-	−1.397
C10	6.129	2.267	2.140	4.445	4.032	4.078	3.988	7.621	5.731	−0.910

Table S4. ^{1}H NMR chemical shift for duplex ODN **17**/cDNA (data from NOESY at 278K).

Residue	H1′	H2′	H2′	H3′	H4′	H5′	H5″	H6/H8	H2/Me/H5
C1	5.963	2.130	2.531	4.674	4.112	3.763	3.763	7.777	5.928
C2	5.459	2.230	2.483	4.882	4.149	4.058	4.021	7.644	5.706
A3	6.323	2.816	2.966	5.074	4.477	4.096	4.192	8.429	7.743
T4	5.977	2.001	2.495	4.918	4.194	4.192	4.330	7.271	1.389
T5	6.188	2.096	2.673	5.204	-	4.257	4.546	7.320	1.679
A6	6.222	2.629	2.934	5.012	4.444	4.221	4.296	8.402	7.712
T7	5.497	1.997	2.332	4.842	4.115	4.117	4.255	7.161	1.458
A8	5.999	2.669	2.848	5.021	4.382	4.119	4.037	8.144	7.237
G9	5.776	2.449	2.637	4.938	4.341	4.196	4.196	7.664	-
C10	6.100	2.118	2.159	4.858	4.031	4.216	4.216	7.356	5.256
G11	5.996	2.692	2.780	4.844	4.264	3.735	3.735	7.984	-
C12	6.065	2.176	2.552	4.828	4.264	4.127	4.194	7.547	5.325
T13	5.721	2.293	2.593	4.920	4.196	4.119	4.196	7.477	1.676
A14	6.250	2.685	2.961	5.032	4.447	4.124	4.196	8.404	7.204
T15	5.590	1.939	2.347	4.865	4.134	4.130	4.305	7.159	1.435
A16	5.841	2.744	2.857	5.051	4.386	4.071	4.133	8.264	6.784
A17	6.175	2.548	2.908	5.024	4.460	4.210	4.210	8.248	7.552
T18	5.574	1.744	2.159	4.782	4.041	4.074	4.231	6.996	1.401
G19	5.609	2.615	2.683	4.935	4.312	4.002	4.047	7.779	-
G20	6.132	2.501	2.324	4.637	4.208	4.104	4.104	7.778	-

Table S5. 1H NMR chemical shift for duplex ODN **18**/cDNA (data from NOESY at 278K).

Residue	H1′	H2′	H2′	H3′	H4′	H5′	H5″	H6/H8	H2/Me/H5
C1	5.960	2.090	2.514	4.660	4.101	3.746	3.746	7.751	5.914
C2	5.375	2.199	2.440	4.867	4.127	4.040	4.005	7.622	5.697
A3	6.359	2.824	3.003	5.070	4.488	4.086	4.179	8.434	7.747
T4	5.929	2.032	2.580	4.832	4.232	4.185	4.362	7.245	1.433
T5	5.885	2.259	2.613	4.927	4.203	4.105	4.189	7.425	1.629
A6	6.189	2.627	2.936	5.012	4.431	4.200	4.200	8.312	7.013
T7	5.556	2.021	2.382	4.857	4.159	4.134	4.281	7.164	1.337
A8	6.009	2.675	2.856	5.032	4.395	4.152	4.063	8.155	7.281
G9	5.784	2.453	2.638	4.943	4.350	4.212	4.171	7.671	-
C10	6.101	2.148	2.119	4.455	4.032	4.219	4.244	7.369	5.286
G11	5.993	2.683	2.772	4.837	4.252	3.725	3.725	7.980	-
C12	6.059	2.152	2.532	4.821	4.258	4.092	4.192	7.535	5.318
T13	5.676	2.235	2.538	4.900	4.183	4.101	4.176	7.448	1.655
A14	6.247	2.681	2.950	5.031	4.446	4.145	4.179	8.397	7.223
T15	5.560	2.049	2.448	4.869	4.181	4.141	4.291	7.184	1.466
A16	5.953	2.678	2.927	5.047	4.404	4.118	4.168	8.207	6.477
A17	6.111	2.509	2.879	5.003	4.457	4.273	4.217	8.156	7.540
T18	5.646	1.740	2.173	4.810	4.090	4.088	4.266	6.992	1.295
G19	5.570	2.639	2.674	4.946	4.335	3.997	4.082	7.809	-
G20	6.151	2.515	2.331	4.645	4.223	4.098	4.098	7.798	-

Supplementary Figures

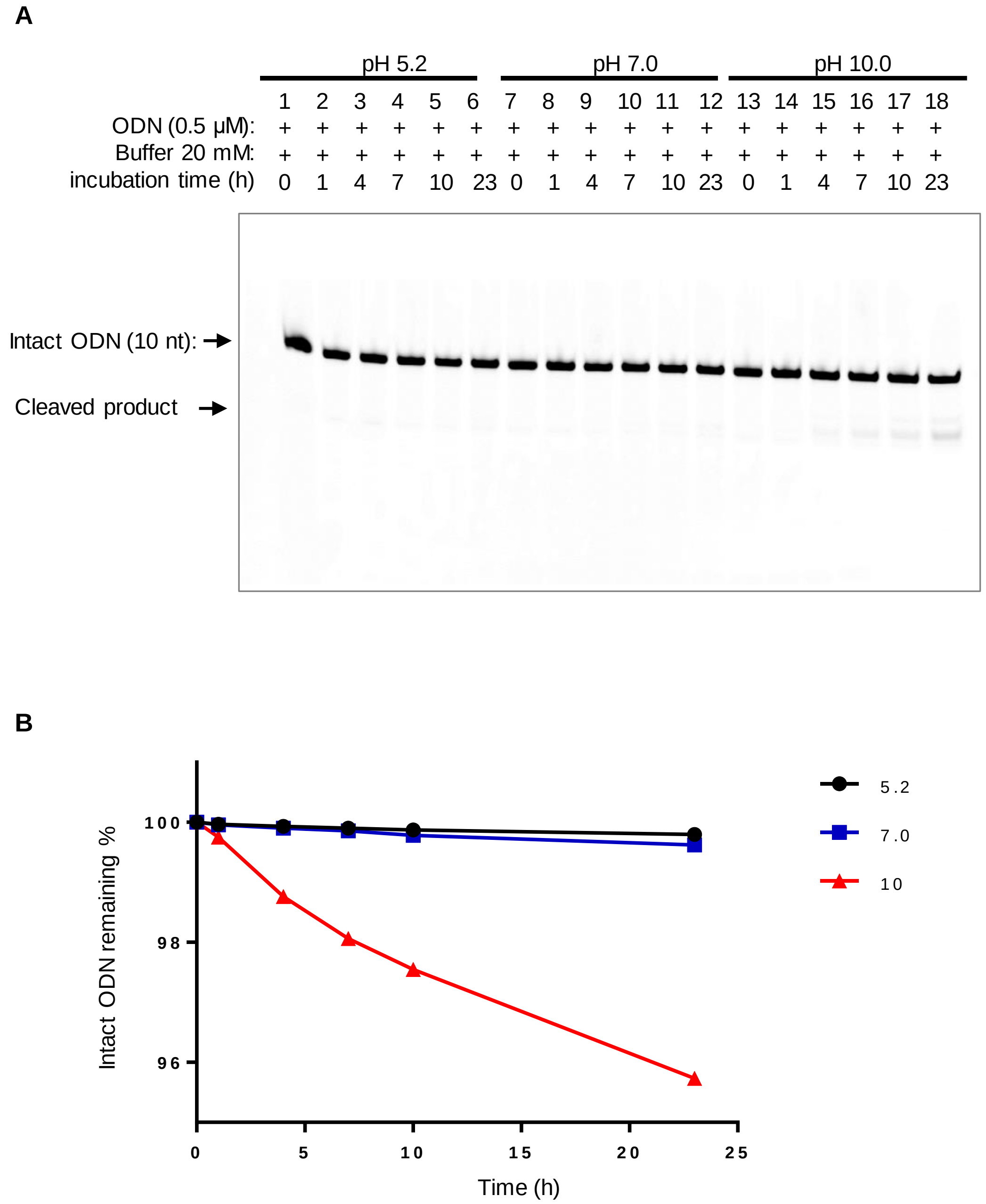

Figure S1. Stability of 5'-FAM labeled ODN **17:** (5'-FAM-CCATT$^{4'\text{-}SCF_3}$ATAGC) in different solutions. A) 20% denaturing PAGE analysis of the stability of 5'-FAM labeled ODN **17**. B) Time dependent percentage of intact ODN **17** remaining upon incubation in 20 mM sodium acetate pH 5.2 (black), 20 mM sodium phosphate pH 7.0 (blue) and 20 mM borax-sodium hydroxide pH 10.0 (red).

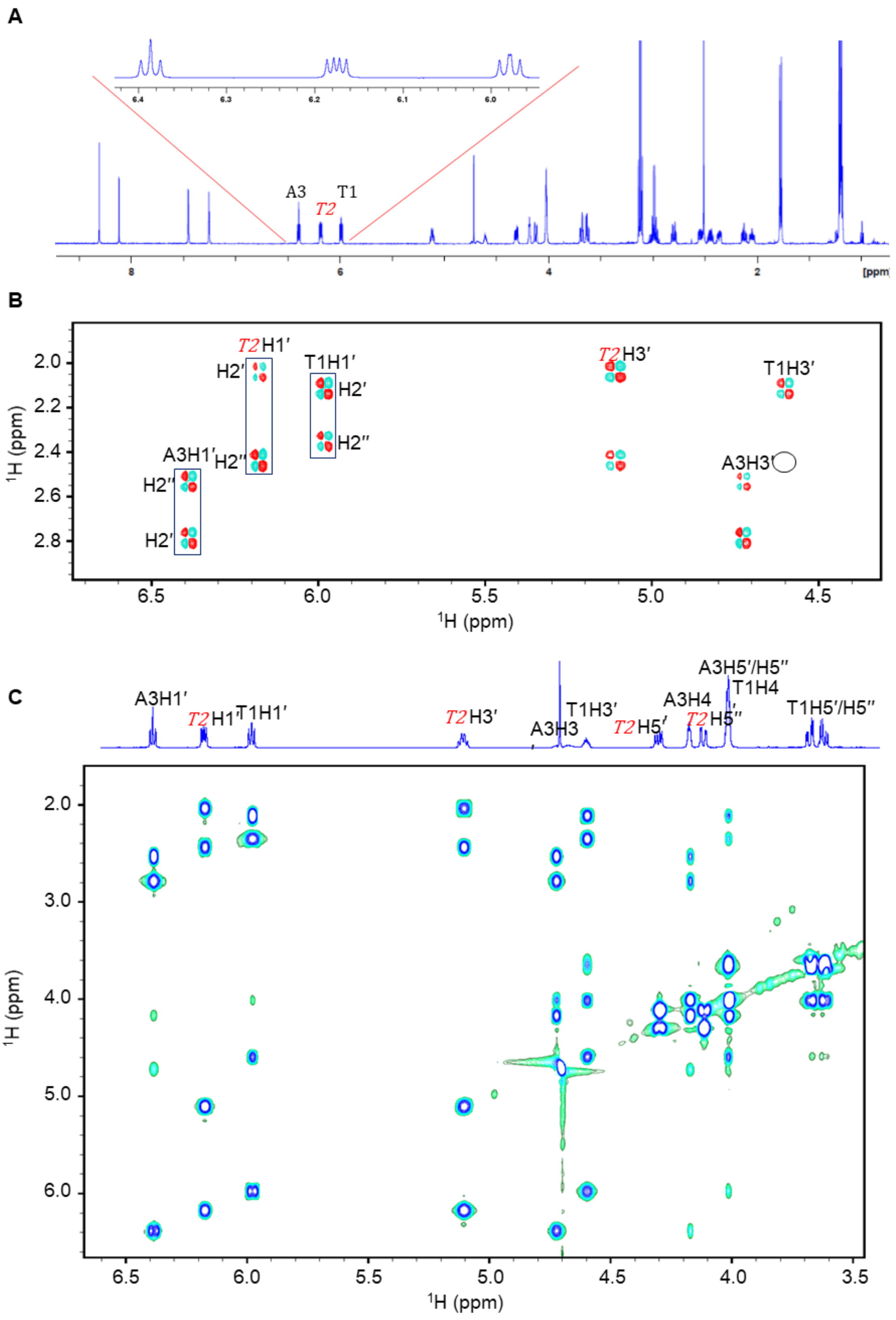

Figure S2. NMR spectra of ODN **19** (5'-TT$^{4'\text{-}SCF_3}$A), T$^{4'\text{-}SCF_3}$ simplified into *T2*. A) ^{1}H NMR spectrum. B) Anomeric-aromatic region of DQF-COSY NMR spectrum. C) Sugar-sugar region of TOCSY NMR spectrum (mixing time: 60 ms) with corresponding ^{1}H NMR spectrum shown above. Conditions: 0.65 mM ODN, 10 mM Na phosphate, pH 7.0; 100%D_2O; 298 K.

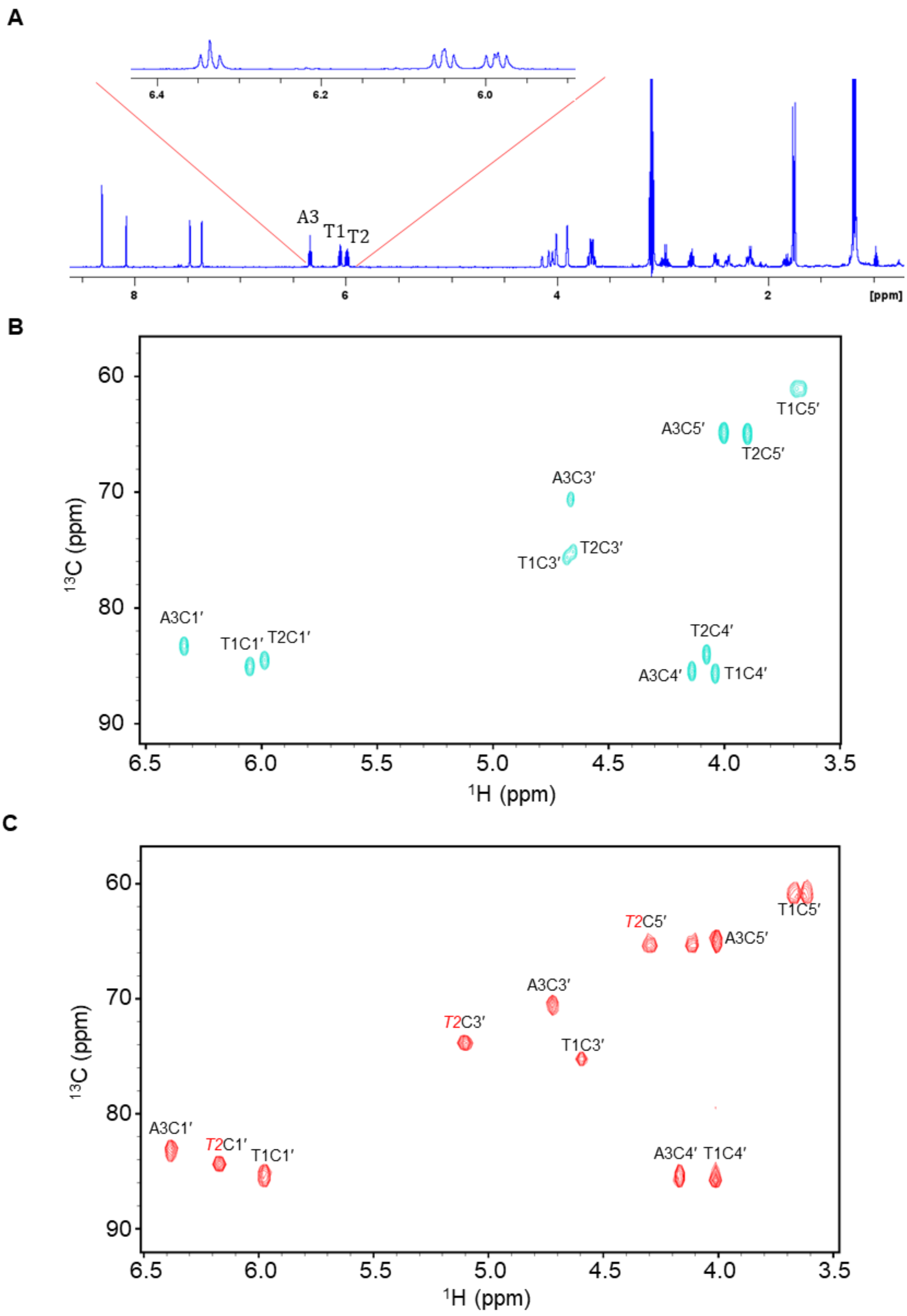

Figure S3. A) ^{1}H NMR spectrum. B) HSQC NMR spectrum of ODN **20** (5'-TTA). C) Anomeric regions of HSQC NMR spectrum of **19** (5'-TT$^{4'-SCF_3}$A) T$^{4'-SCF_3}$ simplified into *T2*. Conditions: 0.65 mM ODN, 10 mM Na phosphate, pH 7.0; 100% D_2O; 298 K.

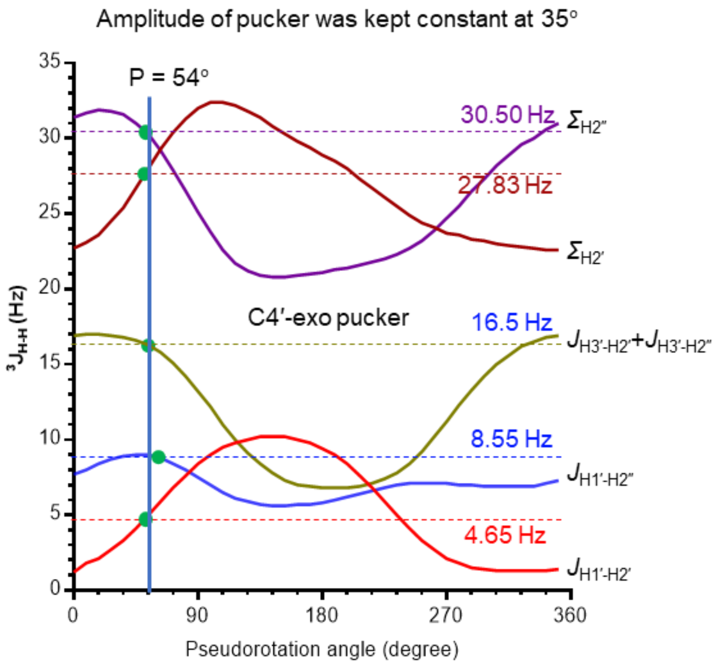

Figure S4. NMR analysis of sugar pucker for T$^{4'\text{-SCF}_3}$ in ODN **19** (5'-TT$^{4'\text{-SCF}_3}$A). Collection of almost all $^3J_{\text{H, H}}$ coupling constants for the middle T$^{4'\text{-SCF}_3}$ in ODN **19** allowed us to perform a more detailed conformational analysis by the graphical method developed by Altona et al.[9] We found that $^3J_{\text{H1'-H2'}}$, $^3J_{\text{H1'-H2''}}$, $\sum_{\text{H2'}}$ ($J_{\text{H1'-H2'}}$ + $J_{\text{H2'-H2''}}$ + $J_{\text{H3'-H2'}}$), $\sum_{\text{H2''}}$ ($J_{\text{H1'-H2''}}$ + $J_{\text{H2'-H2''}}$ + $J_{\text{H3'-H2''}}$), and sums of $^3J_{\text{H2'-H3'}}$ and $^3J_{\text{H2''-H3'}}$ fit very well with a pseudorotation phage angle (P) of 54°

	C1	C2	A3	T4	T5	A6	T7	A8	G9	C10
$J_{H1'-H2'}$ [Hz]	6.70	8.19	7.22	-	5.28	7.98	-	9.43	-	6.70
$J_{H1'-H2''}$ [Hz]	6.70	6.13	6.94	-	8.13	6.25	-	5.65	-	6.65

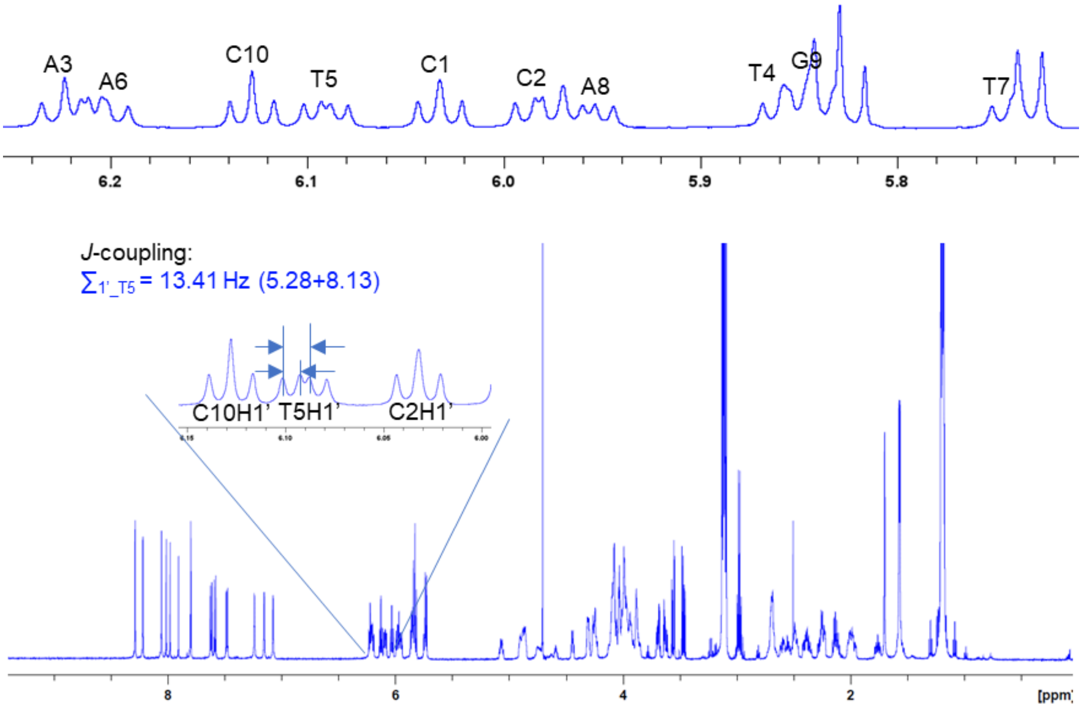

Figure S5. ^{1}H NMR analysis of ODN **17** (5'-CCATT$^{4'-SCF_3}$ATAGC). Conditions: 0.4 mM ODN, 10 mM Na phosphate, pH 7.0; 100%D_2O; 298 K.

A

	C1	C2	A3	T4	T5	A6	T7	A8	G9	C10
Intensity H3'-H6/H8	1.85	3.26	3.52	5.62	20	6.87	5.70	4.24	5.03	3.64

B

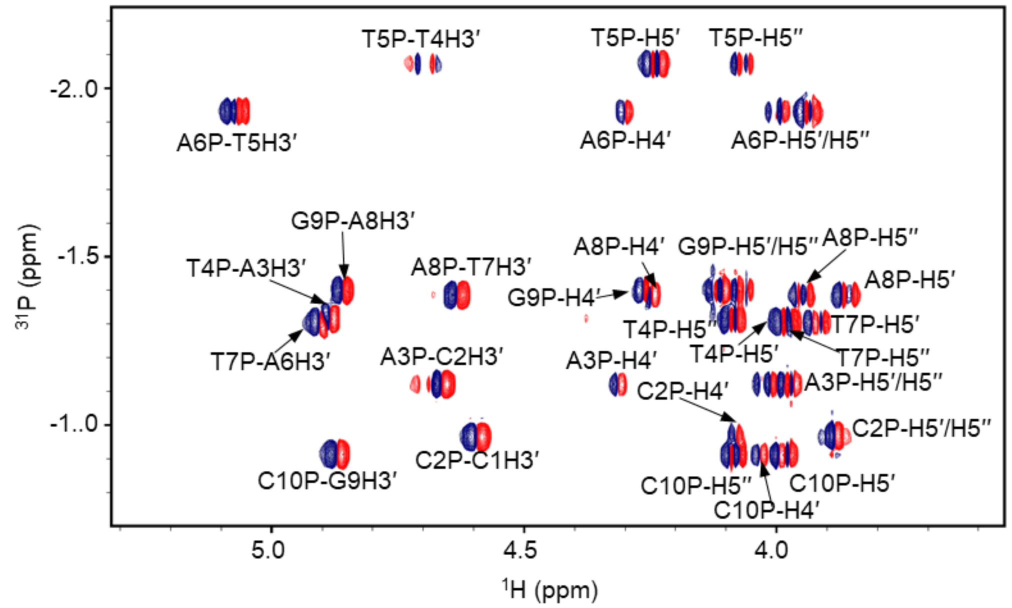

C

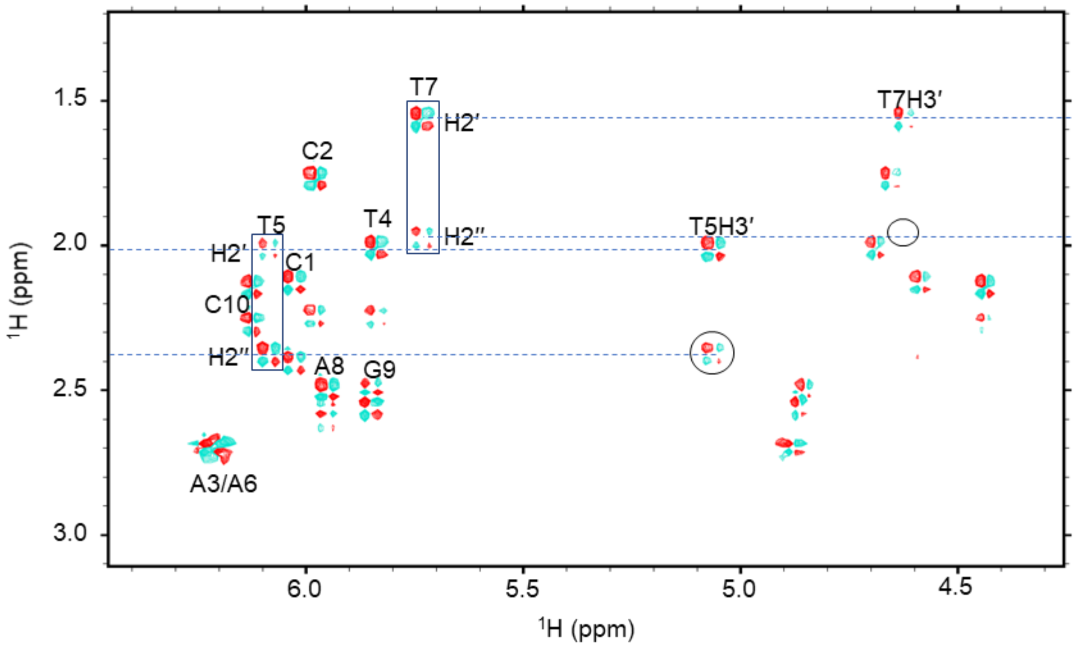

Figure S6. NMR spectrum of ODN **17** (5'-CCATT$^{4'-SCF_3}$ATAGC). A) Relative intensity of NOE signal H3'-H6/H8 from NOESY. B) 2D ^{1}H–^{31}P COSY spectrum. C) DQF-COSY NMR spectrum. Conditions: 0.4 mM ODN, 10 mM Na phosphate, pH 7.0; 100% D_2O; 298 K.

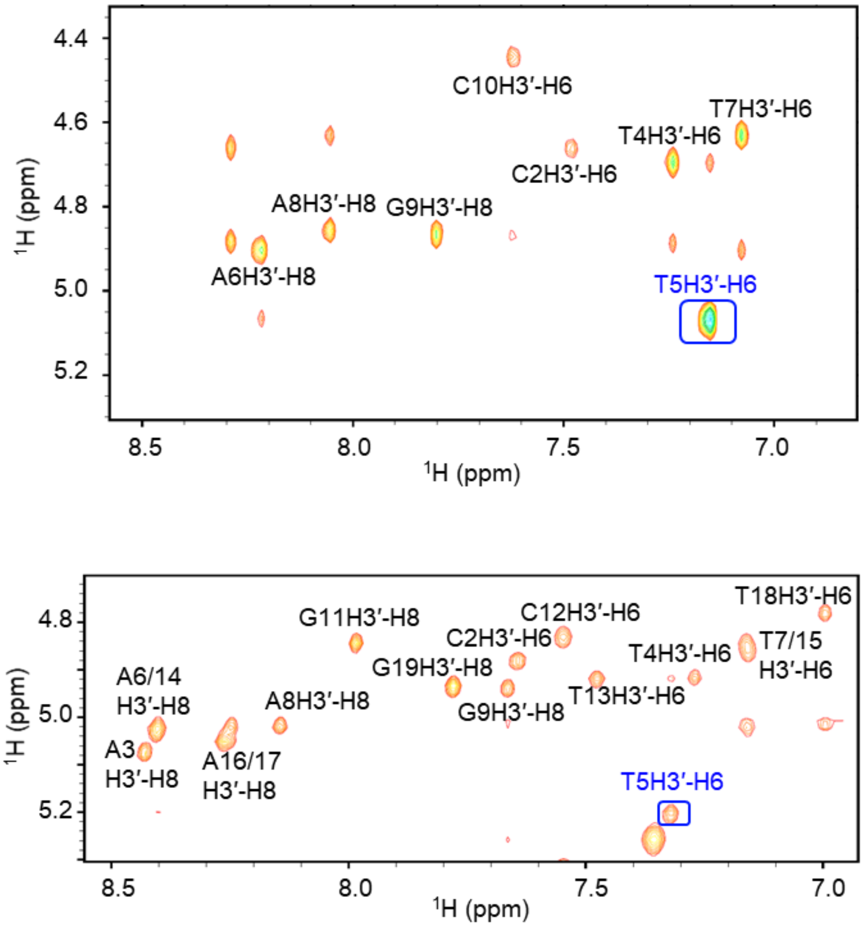

Figure S7. NMR analysis of ODN **17** and ODN **17**/cDNA duplex. H3'-aromatic regions of NOESY NMR spectrum of ODN **17** (mixing time 400 ms) and ODN **17**/cDNA duplex (mixing time 400 ms) are shown in the upper and bottom panel, respectively.

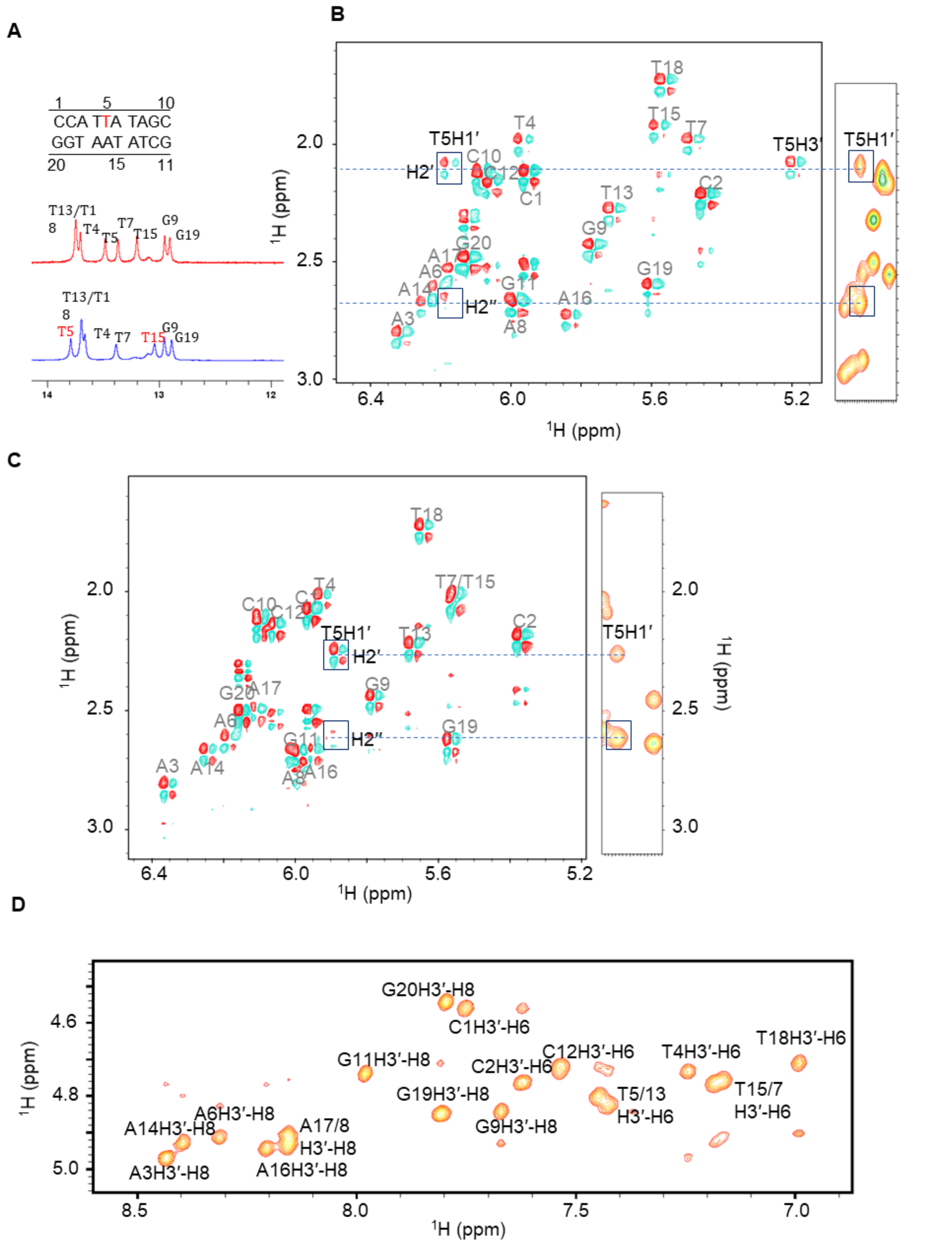

Figure S8. NMR analysis for 10 bp DNA duplexes. A) Imino region of ^{1}H NMR spectra of duplex in H_2O/D_2O (v/v = 9/1), 10 mM K phosphate (pH 7.0); Red, unmodified ODN **18**/cDNA; Blue, modified ODN **17**/cDNA duplex. B) Sections of DQF-COSY NMR spectrum (left) and NOESY spectrum (right) of modified ODN **17**/cDNA duplex; C) Sections DQF-COSY NMR spectra (left) and NOESY spectrum (right) of unmodified ODN **18**/cDNA duplex;. D) H3'-aromatic region of NOESY NMR spectra of unmodified ODN **18**/cDNA duplex (mixing time 200 ms). Conditions: 0.4 mM ODN, 10 mM Na phosphate, pH 7.0; 100% D_2O; 283 K.

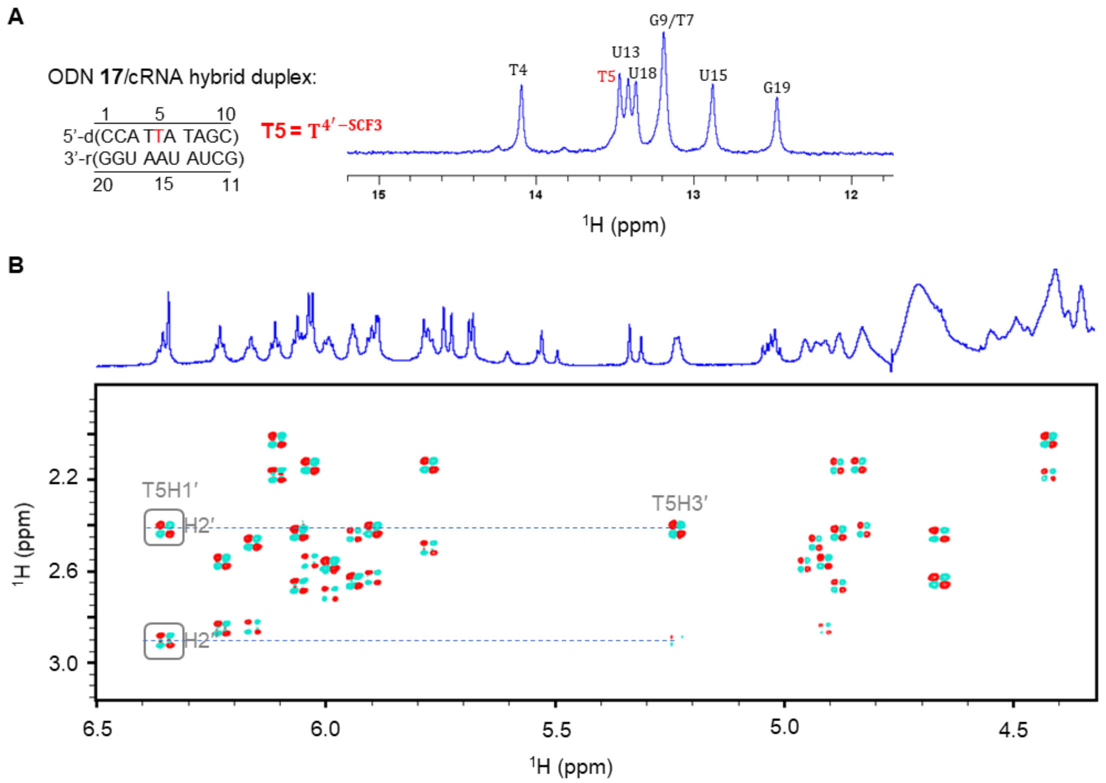

Figure S9. NMR analysis for 10 bp ODN **17**/cRNA hybrid duplex. A) Imino region of ^{1}H NMR spectra of the hybrid duplex in H_2O/D_2O (v/v = 9/1), 10 mM K phosphate (pH 7.0). B) Sections of DQF-COSY NMR spectrum of ODN **17**/cRNA hybrid duplex with corresponding ^{1}H NMR spectrum shown above. Conditions: 0.6 mM ODN, 10 mM Na phosphate, pH 7.0; 100% D_2O; 298 K.

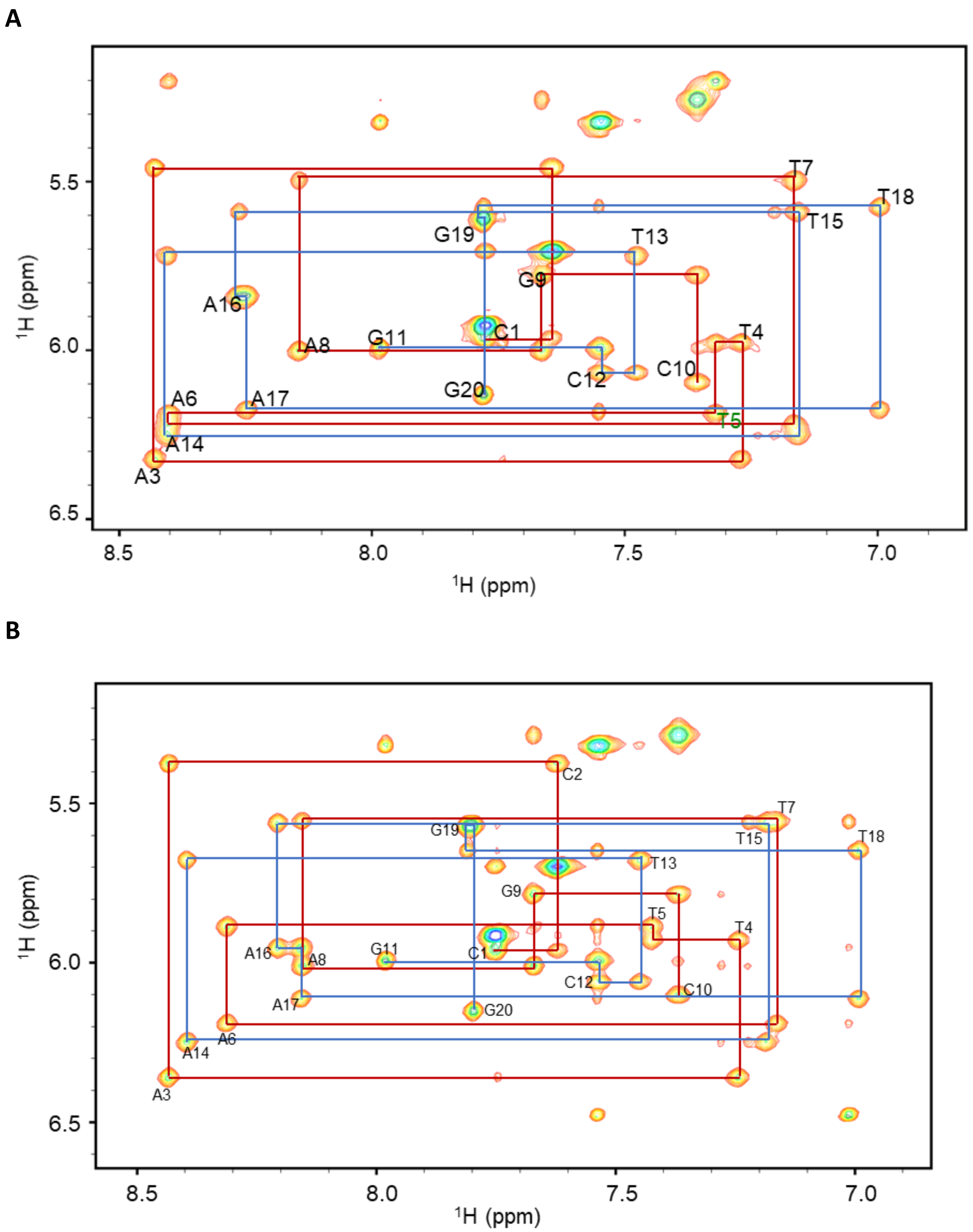

Figure S10. A) The aromatic-anomeric region of NOESY spectrum of modified duplex ODN **17**/cDNA. The sequential NOE cross-peaks are indicated with red lines for the ODN **17** and blue for the complementary DNA. Spectra were acquired in 100% D_2O, 10 mM Na-phosphate buffer, pH 7. B) The aromatic-anomeric region of NOESY spectrum of unmodified duplex ODN **18**/cDNA. The sequential NOE cross-peaks are indicated with red lines for the ODN **18** and blue for the complementary DNA. Spectra were acquired at 200 ms mixing time, in 100% D_2O, 10 mM Na-phosphate buffer, pH 7; 278K.

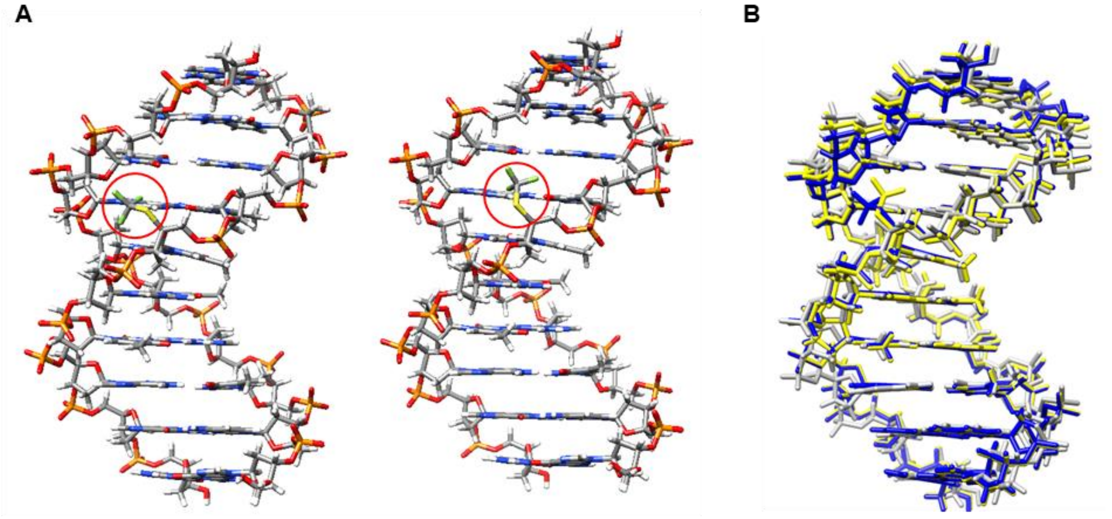

Figure S11. Solution structures of $T^{4'\text{-}SCF_3}$-modified ODN **17**/cDNA duplex and native ODN **18**/cDNA duplex. (A) Two type structures of ODN **17**/cDNA duplex (PDBID:7W0V). The 4'-SCF_3 group is highlighted by a red cycle. (B) Alignment of the structures of modified ODN **17**/cDNA duplex with that of native ODN **18**/cDNA duplex (PDBID:7QA9). The structures were set up through MD calculations with the input of distance restraints obtained from NOESY NMR spectra.

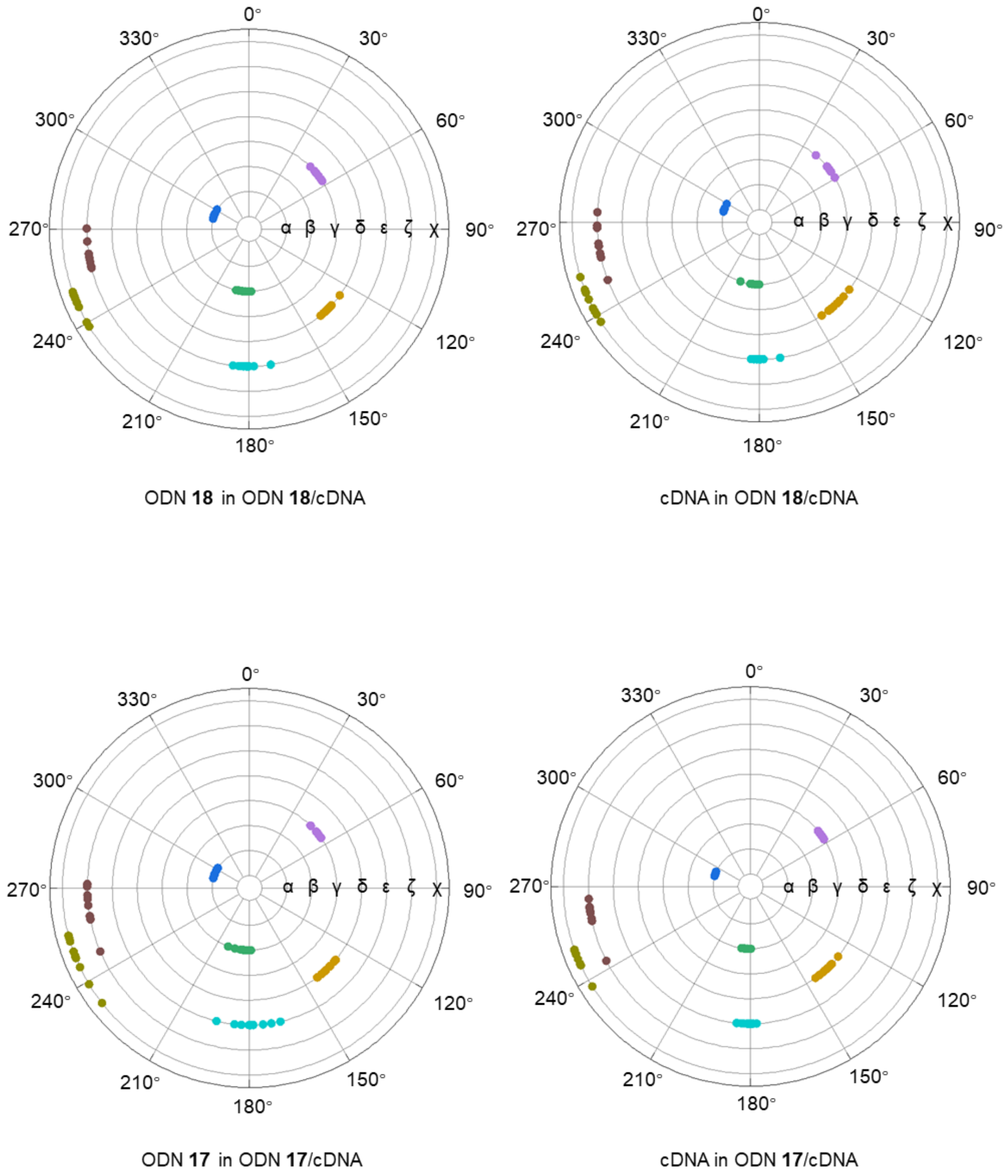

Figure S12. Comparison of torsion angles of duplex ODN **17**/cDNA with that of duplex ODN **18**/cDNA.

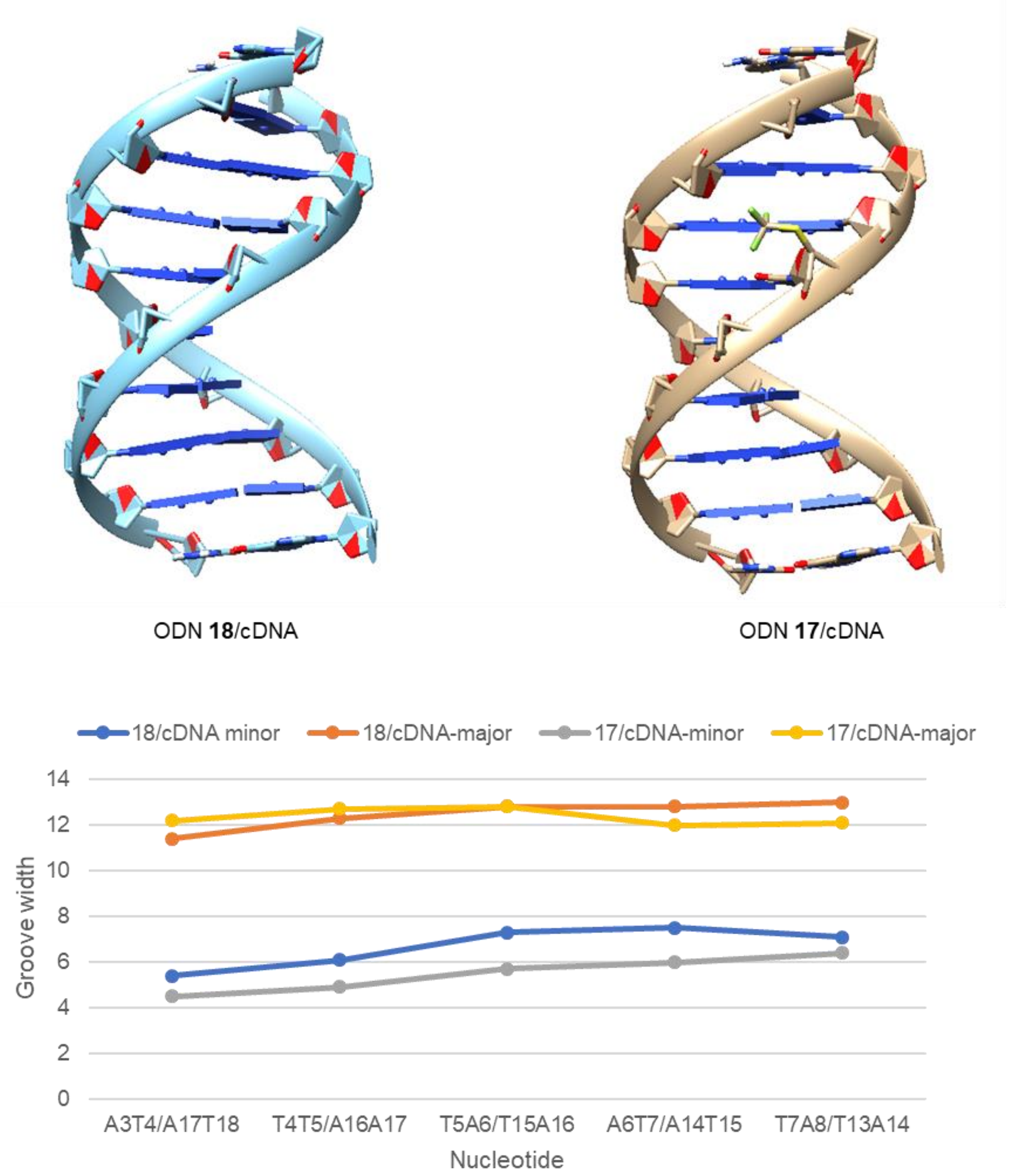

Figure S13. Comparison of the overall structure of ODN **17**/cDNA duplex with that of ODN **18**/cDNA duplex. Groove widths are listed on the bottom.

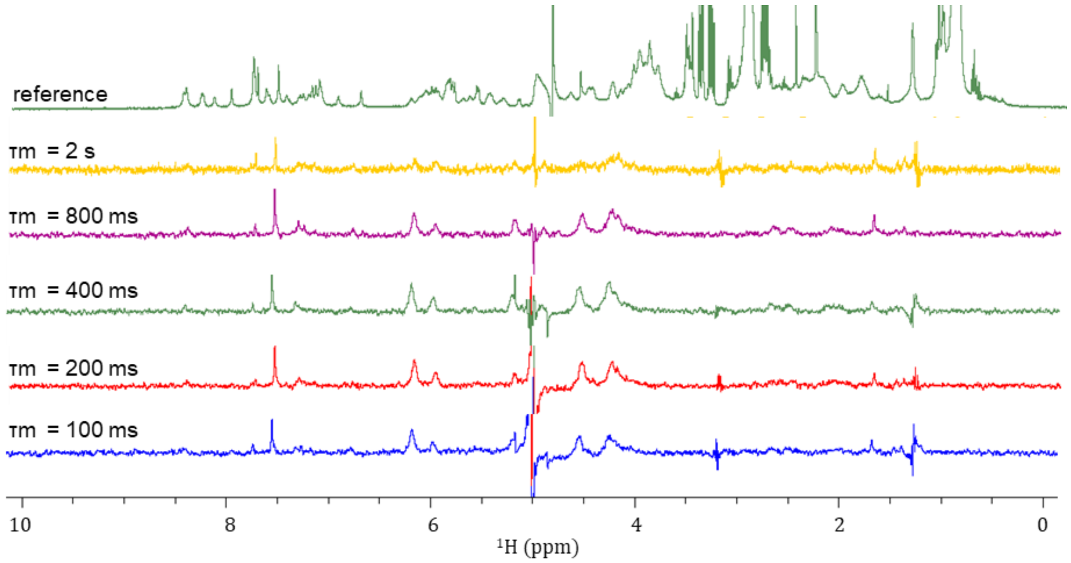

Figure S14. 1D HOESY spectra of duplex ODN **17**/cDNA in different mixing time. Spectra were acquired in 100% D_2O, 10 mM Na-phosphate buffer, pH 7; 278K, 400 MHz (For ^1H).

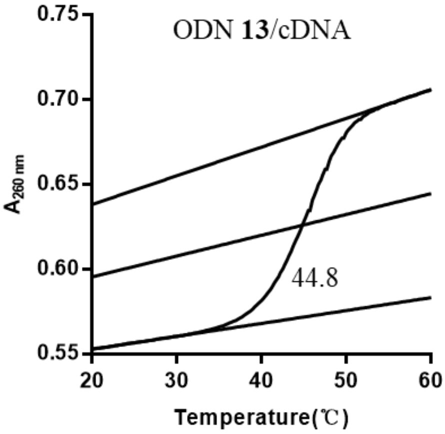
ODN 13/cDNA
44.8
0.75
0.70
0.65
0.60
0.55
20 30 40 50 60
A260 nm
Temperature(℃)

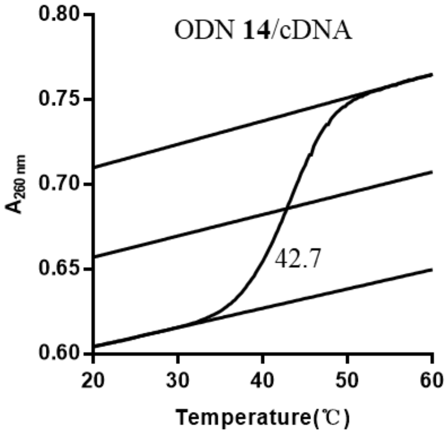
ODN 14/cDNA
42.7
0.80
0.75
0.70
0.65
0.60
20 30 40 50 60
A260 nm
Temperature(℃)

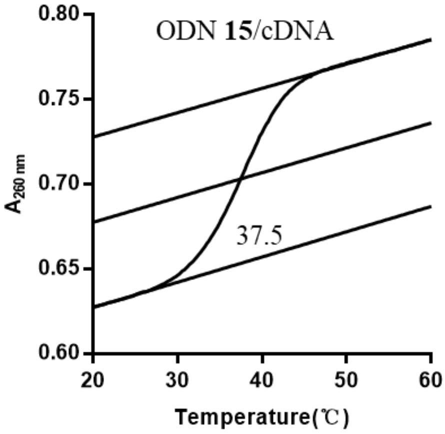
ODN 15/cDNA
37.5
0.80
0.75
0.70
0.65
0.60
20 30 40 50 60
A260 nm
Temperature(℃)

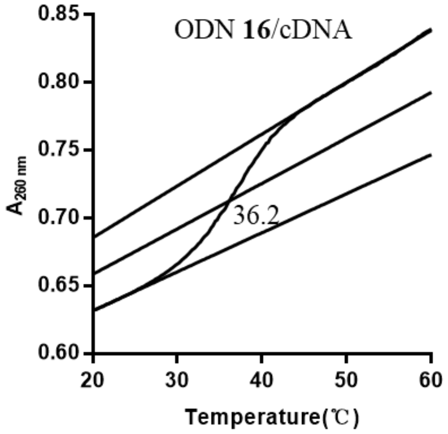
ODN 16/cDNA
36.2
0.85
0.80
0.75
0.70
0.65
0.60
20 30 40 50 60
A260 nm
Temperature(℃)

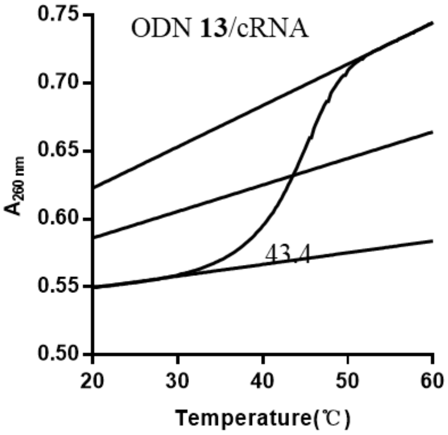
ODN 13/cRNA
43.4
0.75
0.70
0.65
0.60
0.55
0.50
20 30 40 50 60
A260 nm
Temperature(℃)

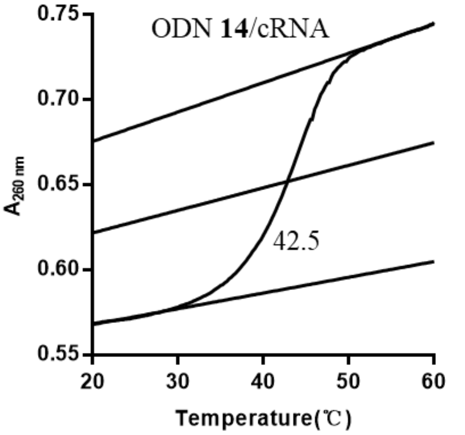
ODN 14/cRNA
42.5
0.75
0.70
0.65
0.60
0.55
20 30 40 50 60
A260 nm
Temperature(℃)

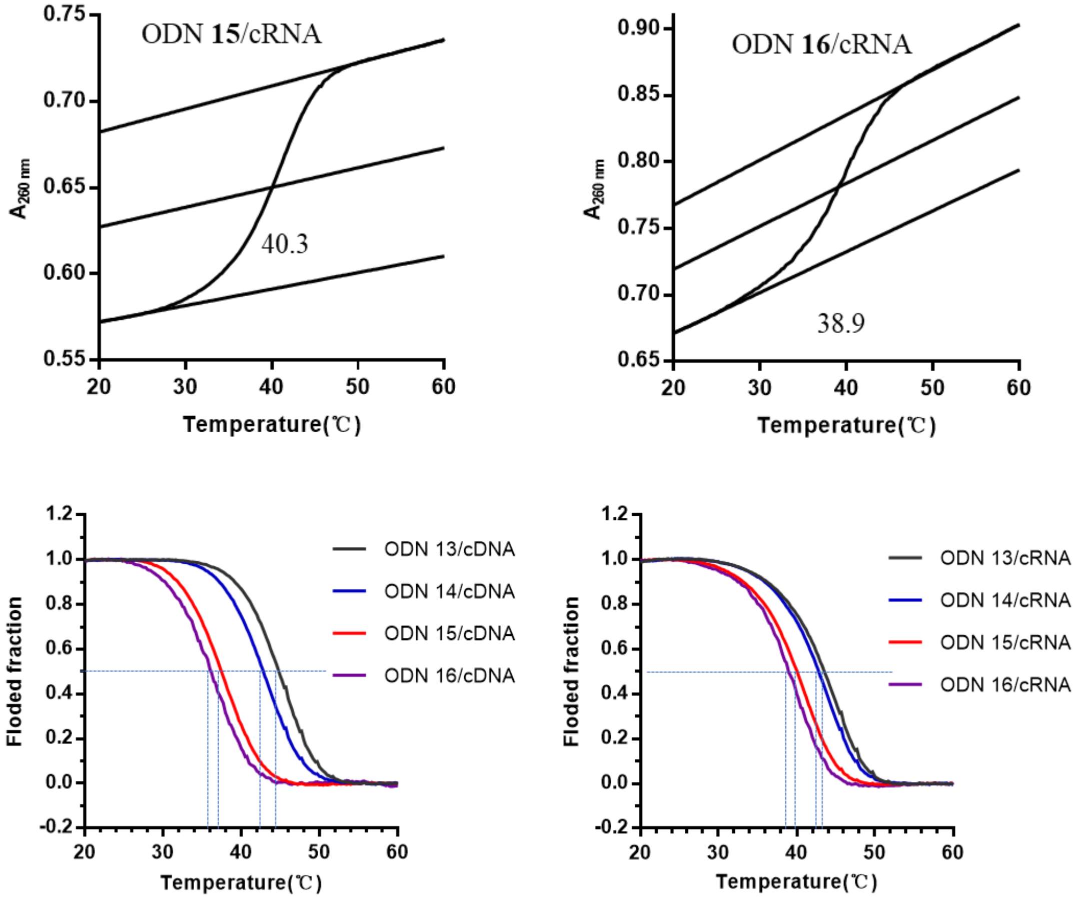

Figure S15. Melting curves of duplex.

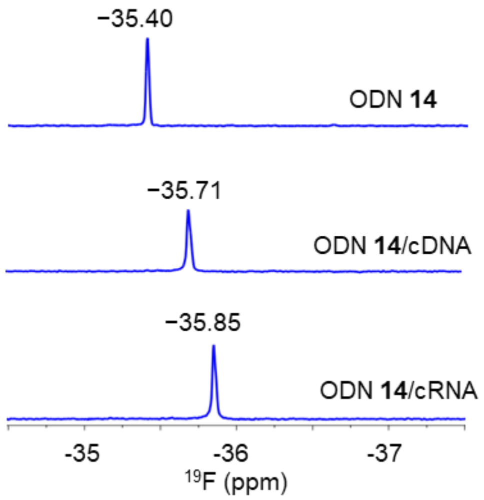

Figure S16. ^{19}F NMR of $T^{4'\text{-}SCF_3}$-modified DNA single strand and duplexes. Conditions: 0.2 mM ODN, 150 mM NaCl, 10% (v/v) D_2O, 376.5 MHz, 293K, ns = 2048.

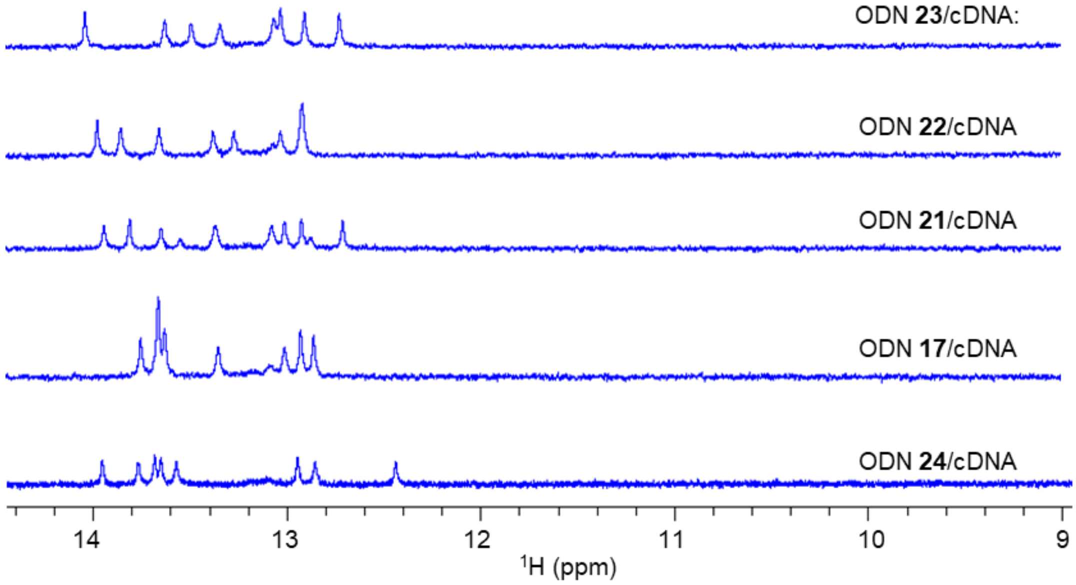

Figure S17. ^{1}H NMR imino region of $T^{4'\text{-}SCF_3}$-modified duplex. Condition: 0.1 mM dsDNA, 10 mM Na-phosphate buffer, pH 7, 10% (v/v) D_2O, 278K, 600 MHz.

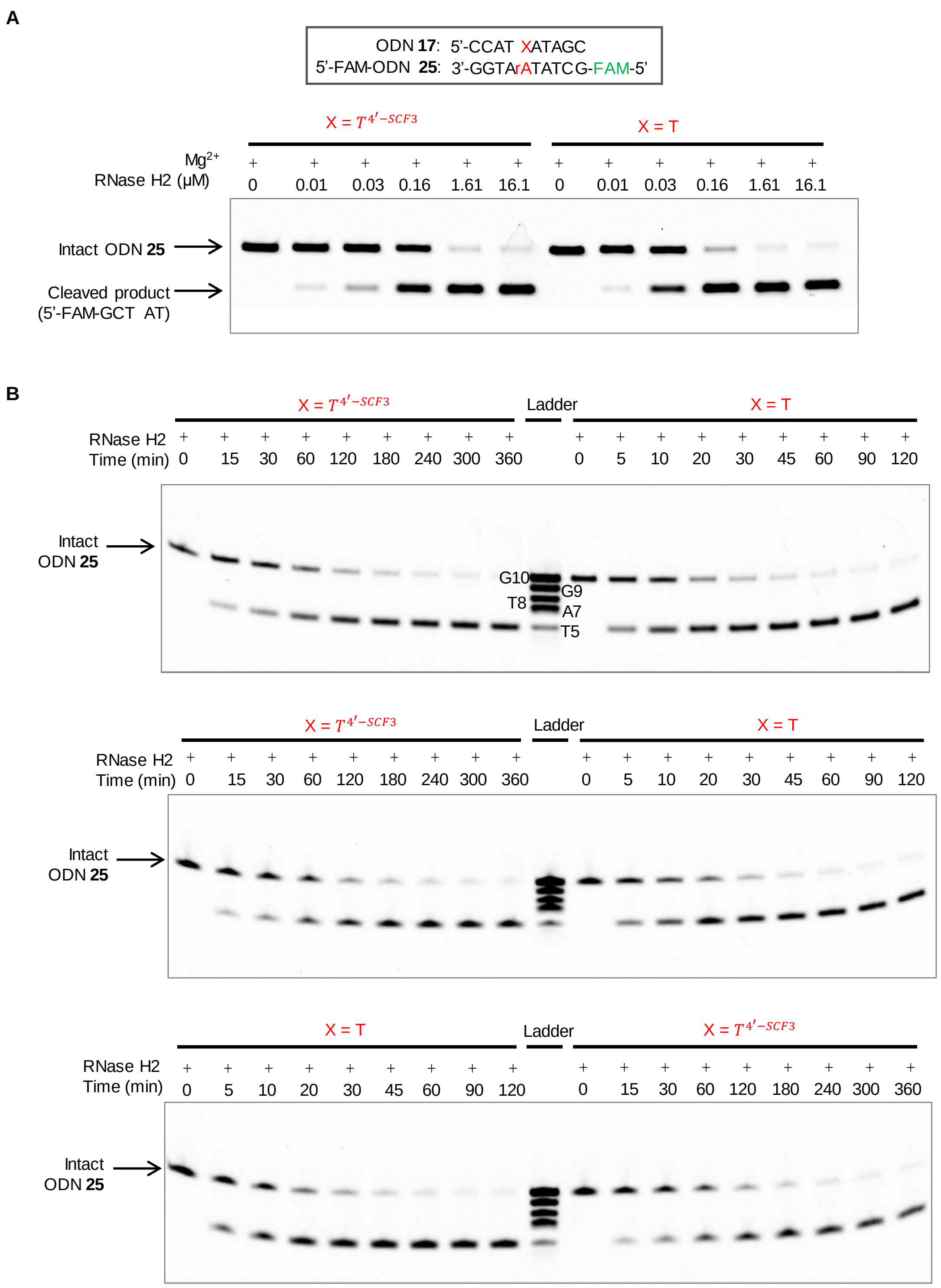

A
ODN 17: 5'-CCAT XATAGC
5'-FAM-ODN 25: 3'-GGTArATATCG-FAM-5'
X = $T^{4'-SCF3}$
X = T
Mg2+
RNase H2 (μM)
0 0.01 0.03 0.16 1.61 16.1 0 0.01 0.03 0.16 1.61 16.1
Intact ODN 25
Cleaved product (5'-FAM-GCT AT)
B
Ladder
RNase H2
Time (min)
0 15 30 60 120 180 240 300 360
0 5 10 20 30 45 60 90 120
Intact ODN 25
G10
G9
T8
A7
T5

C

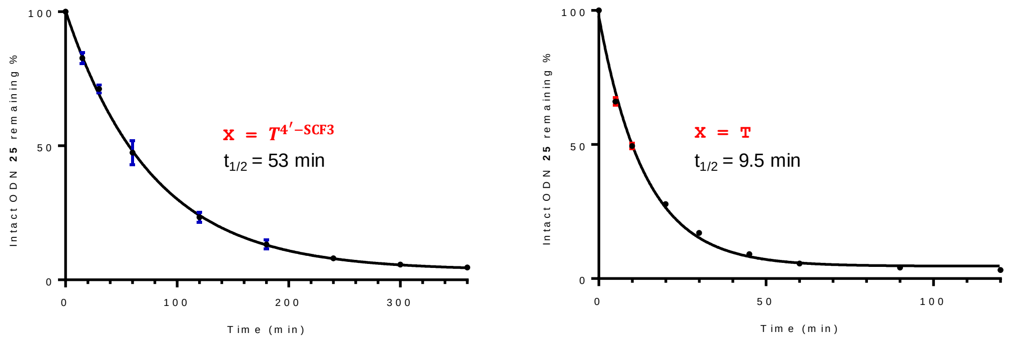

Figure S18. Gel analysis and kinetics of RNase H2 cleavage of DNA duplex (5'-FAM-ODN **25**/ODN **17**) containing an embedded ribonucleotide. A) 20% Denaturing PAGE analysis of strand cleavage under different concentrations of RNase H2. Conditions: 0.5 μM ODNs per strand, various amounts of RNase H2 in reaction buffer [20 mM Tris-HCl, pH 7.5, 10 mM $(NH_4)_2SO_4$, 10 mM KCl, 10 mM $MgCl_2$, 0.1% (w/w) Triton X-100], 21 ºC for 30 min. B) 20% Denaturing PAGE analysis of time-dependent strand cleavage. Conditions: 0.5 μM ODNs per strand, 0.05 μM RNase H2 in reaction buffer [20 mM Tris-HCl, pH 7.5, 10 mM $(NH_4)_2SO_4$, 10 mM KCl, 10 mM $MgCl_2$, 0.1% (w/w) Triton-X100], 21 ºC for various time. This experiment was performed three times. Time dependent percentage of intact ODN **25** remaining is shown in panal C. The plots were fitted using one phase decay exponential function ($Y = [Y_0 - Y_{plateau}] \cdot e^{-kt} + Y_{plateau}$) to give the first order kinetic constants k. Then the $t_{1/2}$ values were calculated according to the equation: $t_{1/2} = \ln 2/k$. Error bars indicate standard deviation from three independent experiments.

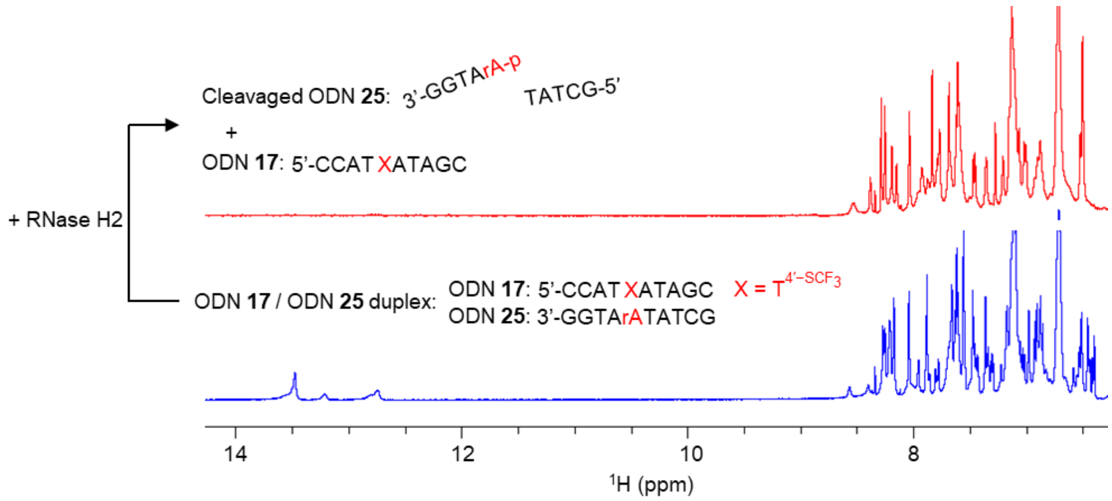

Figure S19. ^{1}H NMR spectra of ODN **17**/ODN **25** duplex before and after cleavage by RNase H2. Conditions: 0.2 mM ODNs per strand, 0 or 16 μM RNase H2 in reaction buffer [20 mM Tris-HCl, pH 7.5, 10 mM $(NH_4)_2SO_4$, 10 mM KCl, 10 mM $MgCl_2$, 0.1% (w/w) Triton-X100], 10% (v/v) D_2O, 600 MHz, 298K.

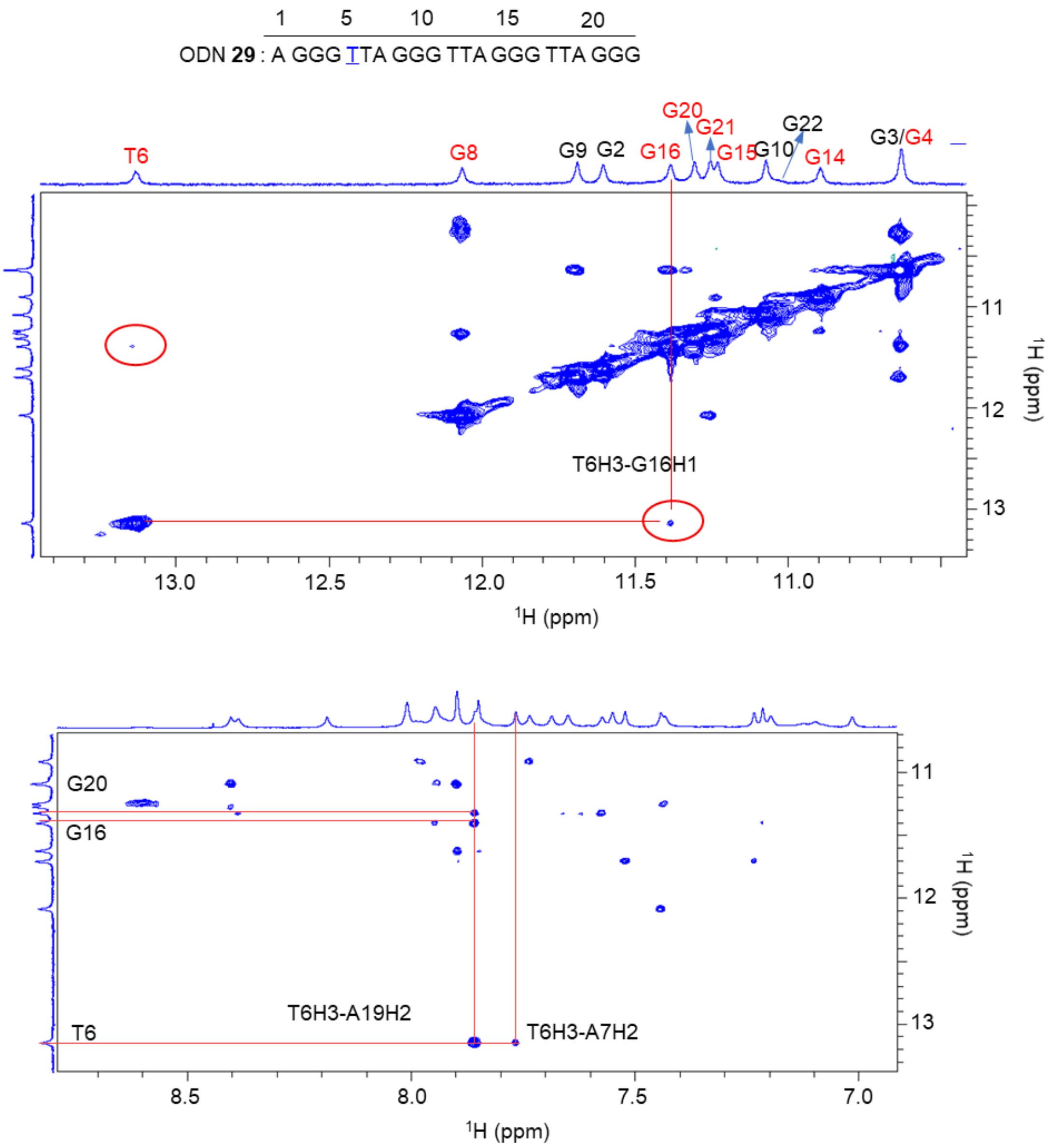

Figure S20. Imino-imino (upper panel) and imino-aromatic (lower panel) regions of NOESY spectra of modified hTEL sequence ODN **29**, with corresponding ^{1}H NMR spectral regions shown above the 2D plots. Spectra were acquired in 10% (v/v) D_2O, 20 mM Na-phosphate buffer, pH 7; 80 mM NaCl, 278K, mixing time 200 ms, 600 MHz. The red lines and ellipses highlight the NOE correlations used to assign imino ^{1}H NMR signal corresponding to T6 H3. The primary DNA sequence of ODN **29** is shown above, with the $T^{4'-SCF_3}$ residue at position 5 designated by blue color. Red labels designate the imino ^{1}H NMR signals of residues (T6, G4, G8, G14, G15, G16, G20, G21) exhibiting (slightly) different chemical shift with respect to native ODN **28**.

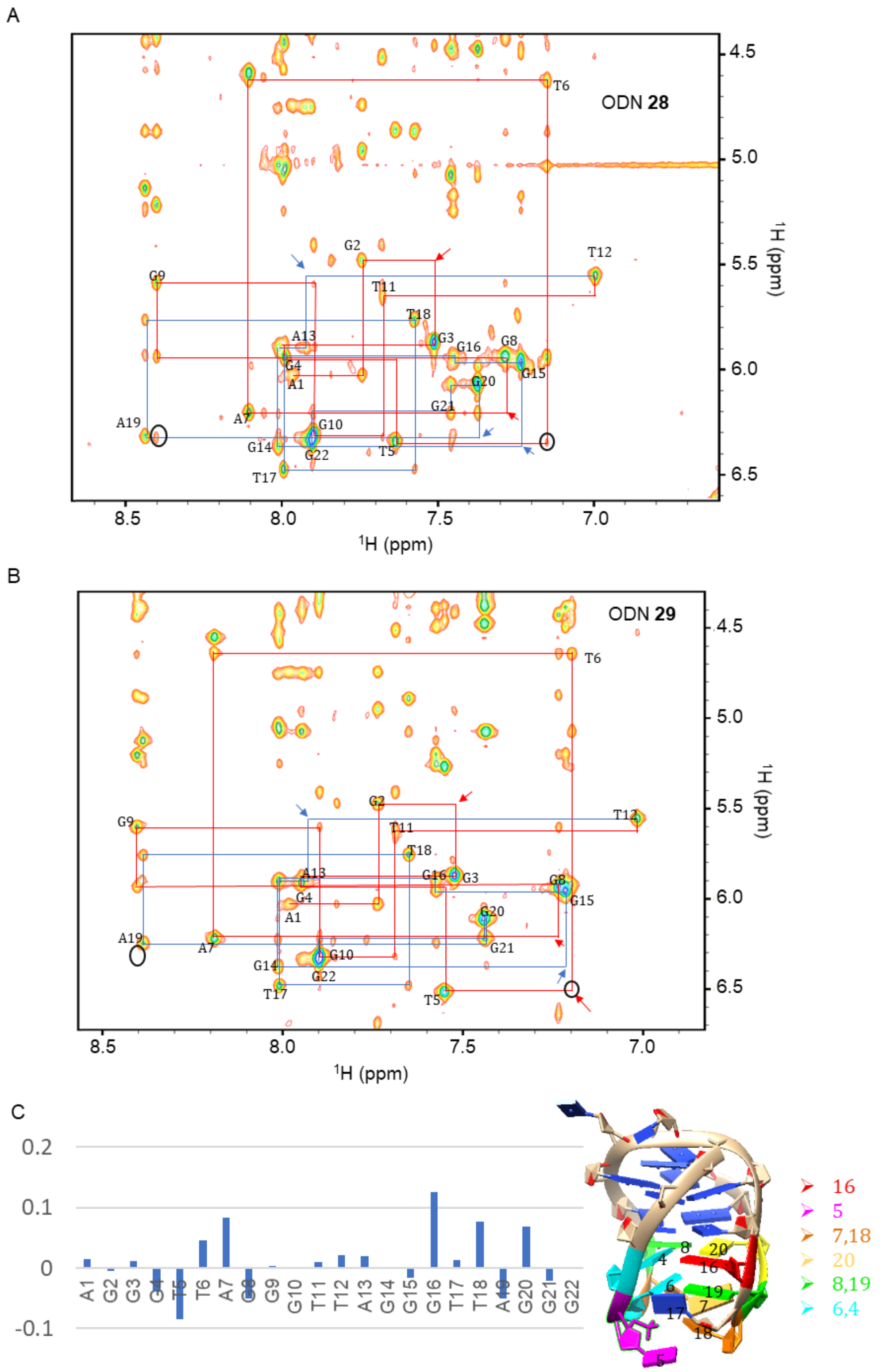

Figure S21. NMR analysis of hTEL-G4 structures with depicted sequential H1'-H6/H8 NOE correlations for ODNs (A) **28** and (B) **29**; C) ^{1}H NMR chemical shift differences between the hTEL-G4 adopted by ODNs **28** and **29** (left side) and color-coded distances between T5 H4' and nearby aromatic protons determined from the structural model PDB ID 143D[10] (right side). Condition: 0.2 mM ODNs, 20 mM Na phosphate, pH 7.0; 80 mM NaCl, 10% (v/v) D_2O; 278K, 600 MHz.

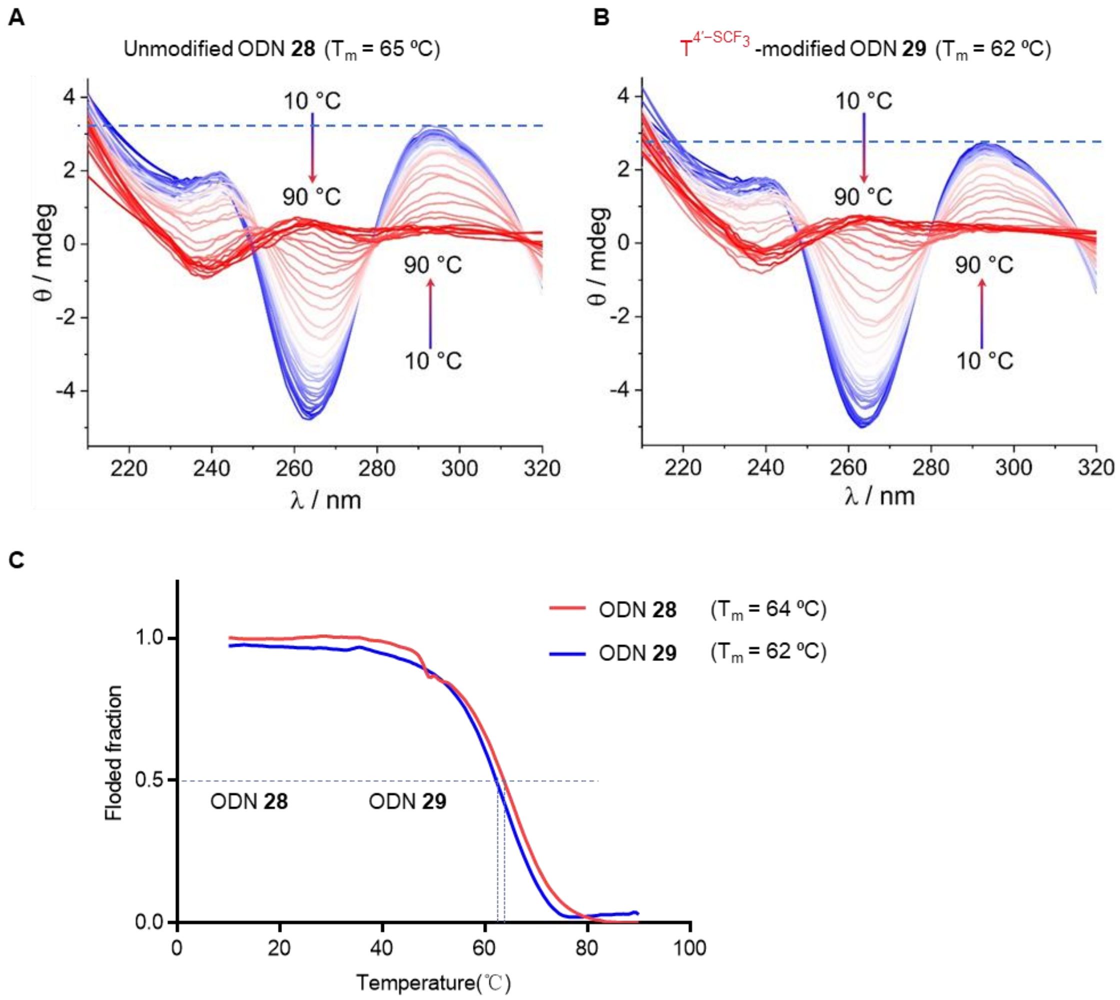

Figure S22. Melting analysis of hTEL-G4. (A) and (B) CD melting analysis of native ODN **28** and $T^{4'\text{-}SCF_3}$-modified ODN **29**. Conditions: 10 μM ODNs in 10 mM Na phosphate, pH 7.0; 210−320 nm wavelength range. (C) UV melting analysis of ODN **28** and ODN **29**. Conditions: 2 μM ODNs in 20 mM Na phosphate, pH 7.0, 70 mM NaCl.

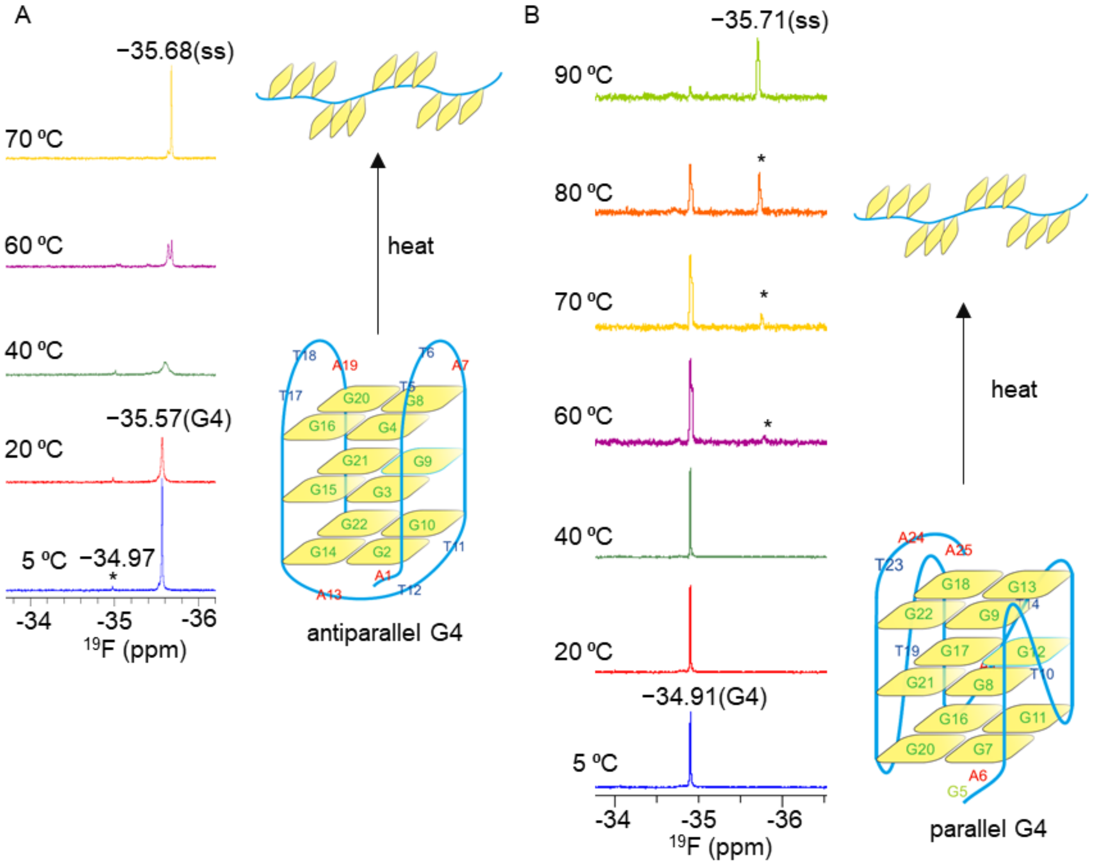

Figure S23. Temperature dependent ^{19}F NMR of ODN **29** in Na^+ ions containing solution (A) and ODN **27** in K^+ ions containing solution (B). * is used to denote fingerprint of the minor parallel G-quadruplex species (panel A) and single-stranded, i.e. unfolded species (panel B).

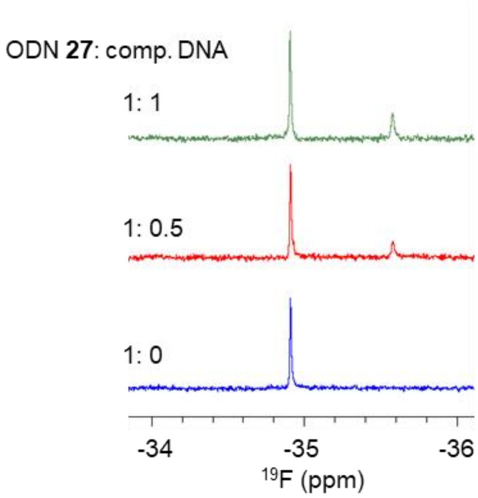

Figure S24. ^{19}F NMR of cMYC sequence ODN **27** upon addition of complementary DNA in K^+ solution: 25 mM K phosphate, pH 7.0, 70 mM KCl, 10% (v/v) D_2O, 564.7 MHz.

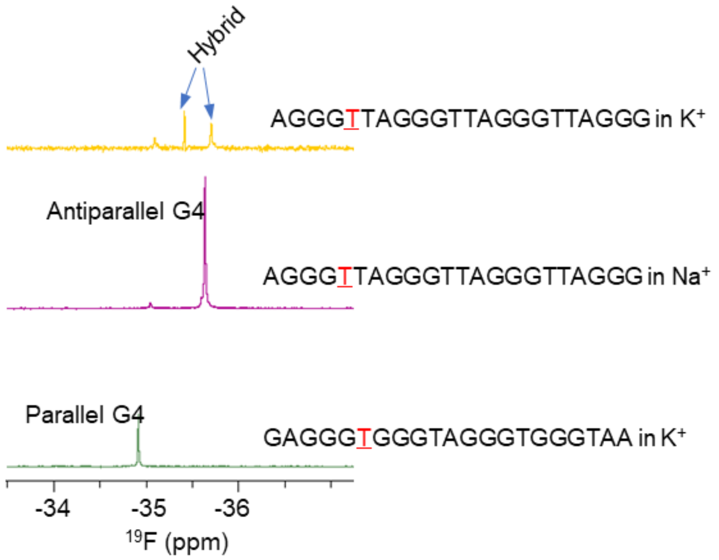

Figure S25. ^{19}F NMR spectra of G4-forming ODNs. Yellow: ODN **29** in 25 mM K phosphate, pH 7.0, 70 mM KCl, 10% (v/v) D_2O. Purple: ODN **29** in 20 mM Na phosphate, pH 7.0; 80 mM NaCl, 10% D_2O. Green: ODN **27** in 25 mM K phosphate, pH 7.0, 70 mM KCl, 10% (v/v) D_2O. Spectra were recorded at 278K. In the primary DNA sequence of ODNs the $T^{4'\text{-}SCF_3}$ residue is highlighted in red.

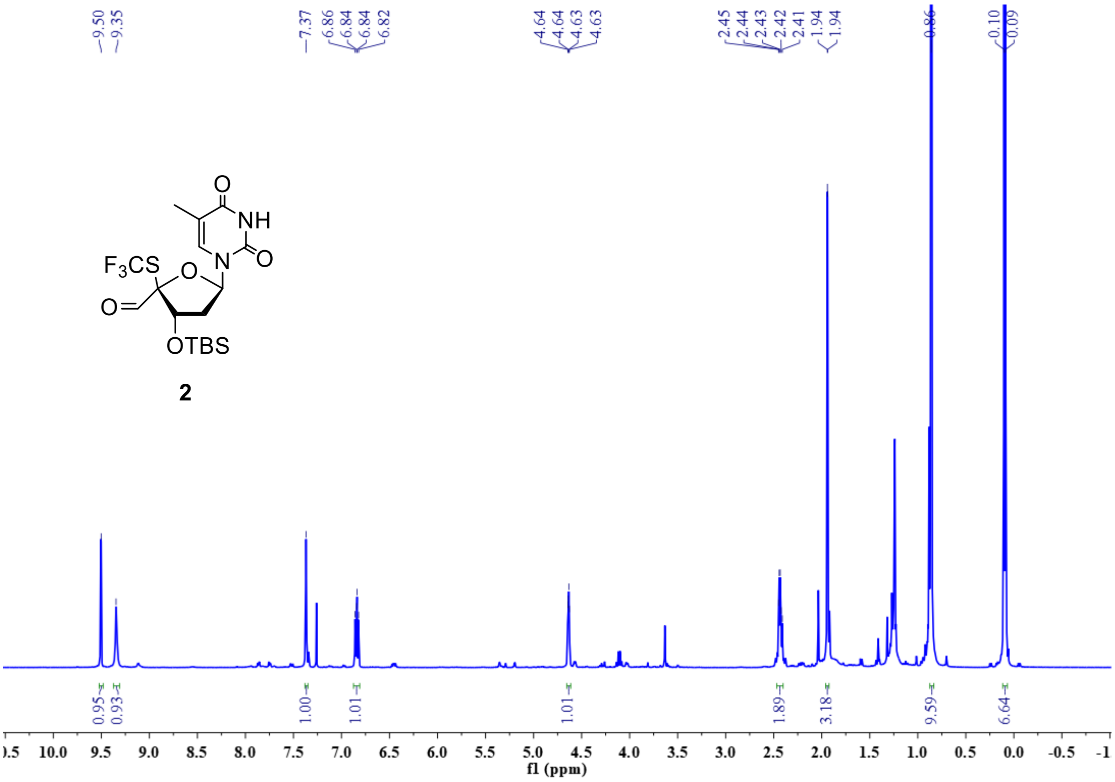

Figure S26. ^{1}H NMR (400 MHz, $CDCl_3$) of compound **2**.

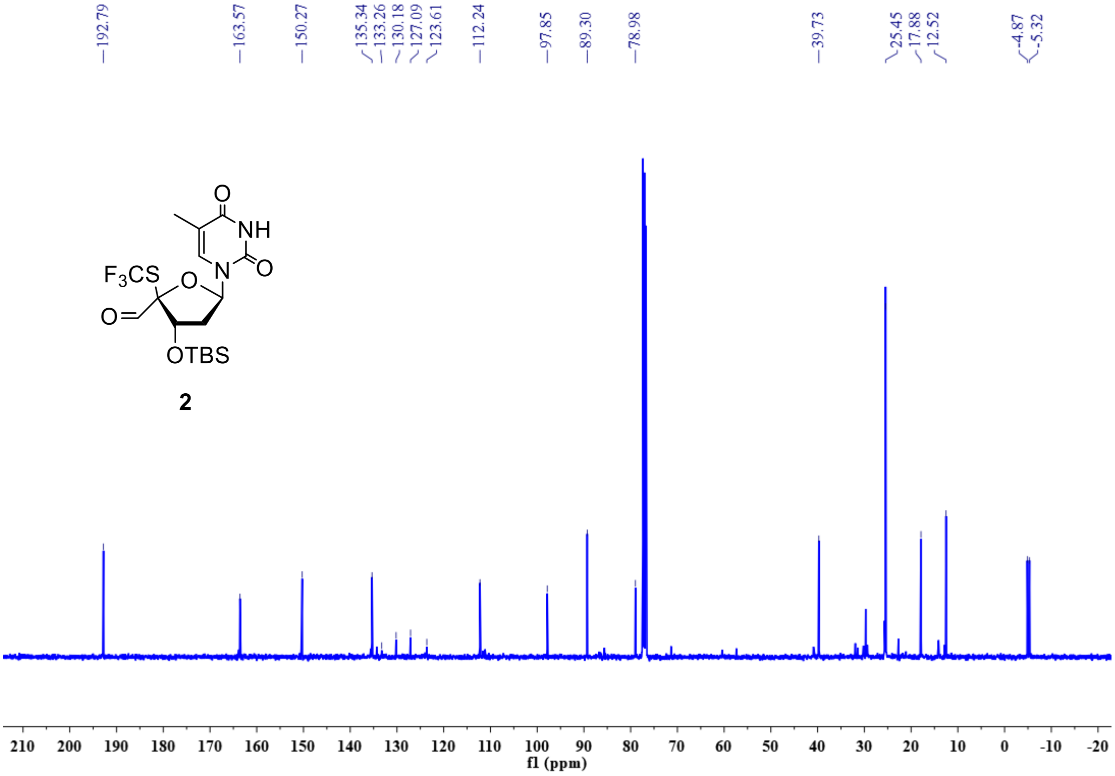

Figure S27. ^{13}C NMR (100.6 MHz, $CDCl_3$) of compound **2**.

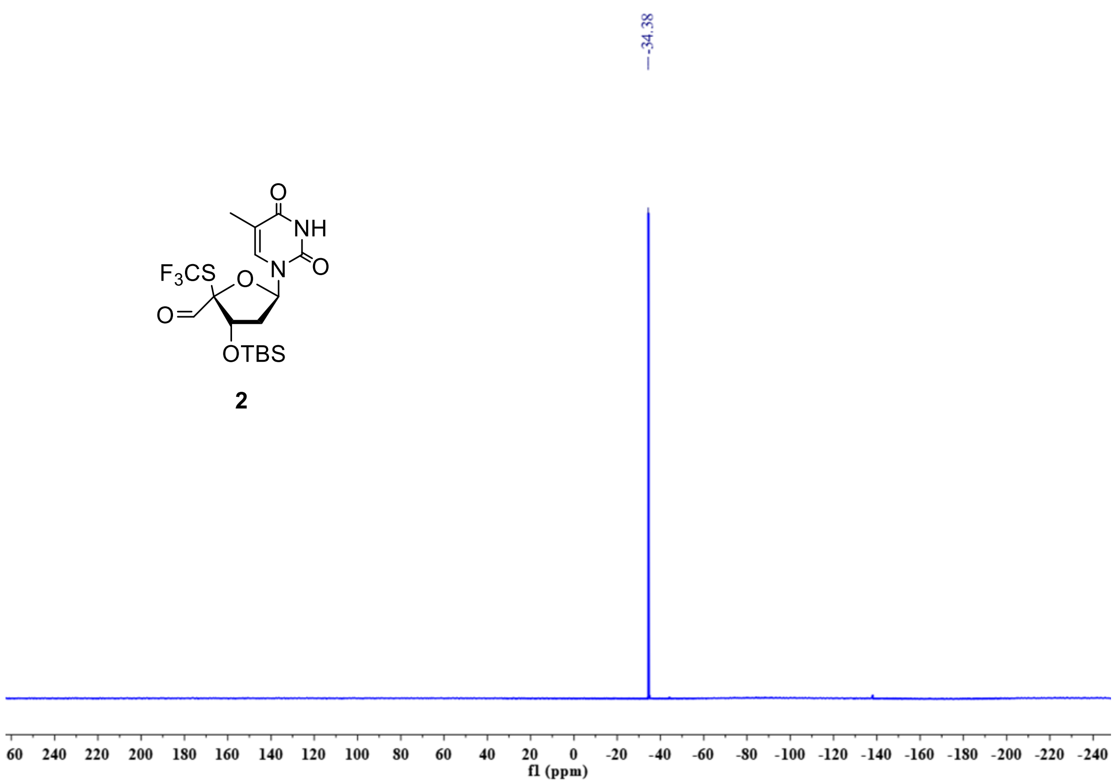

Figure S28. ^{19}F NMR (376.5 MHz, $CDCl_3$) of compound **2**

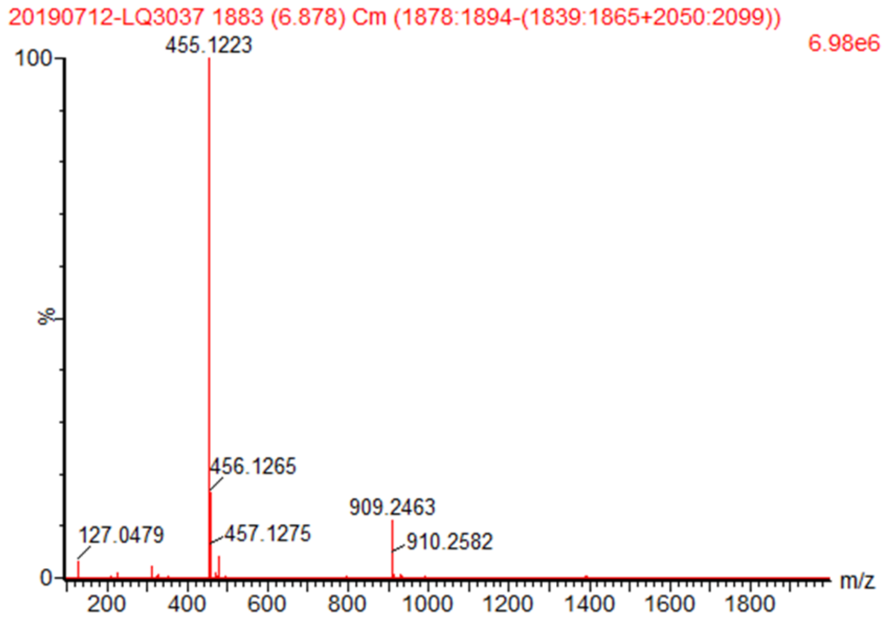

Figure S29. HRMS of compound **2**.

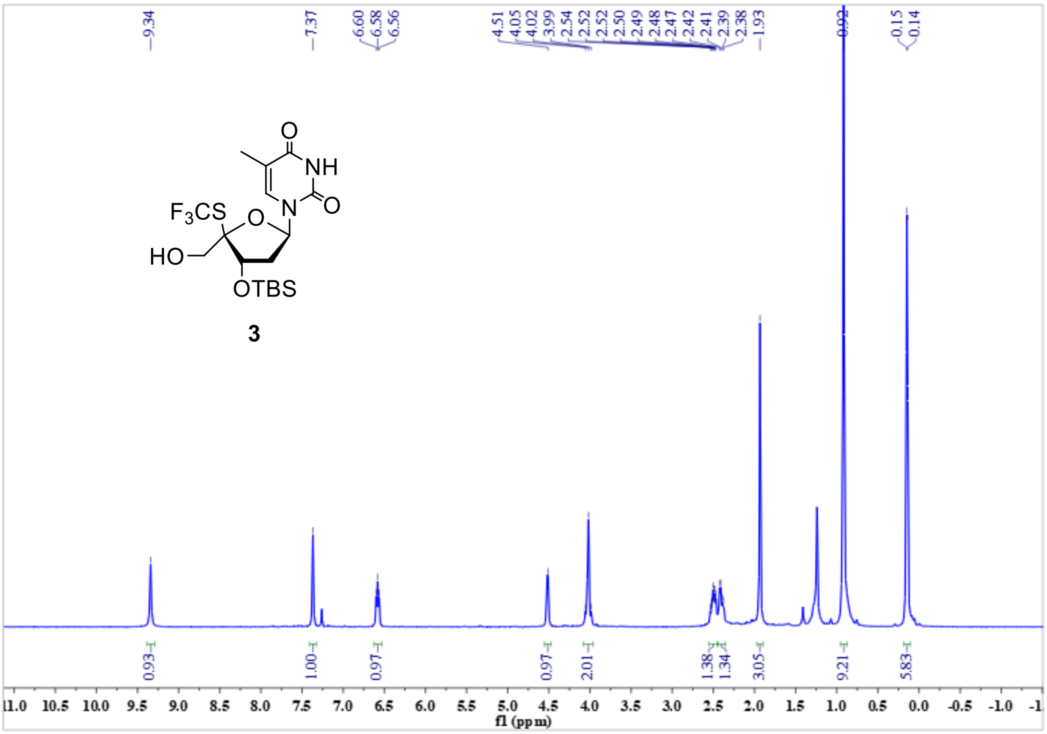

Figure S30. ^{1}H NMR (400 MHz, $CDCl_3$) of compound **3**.

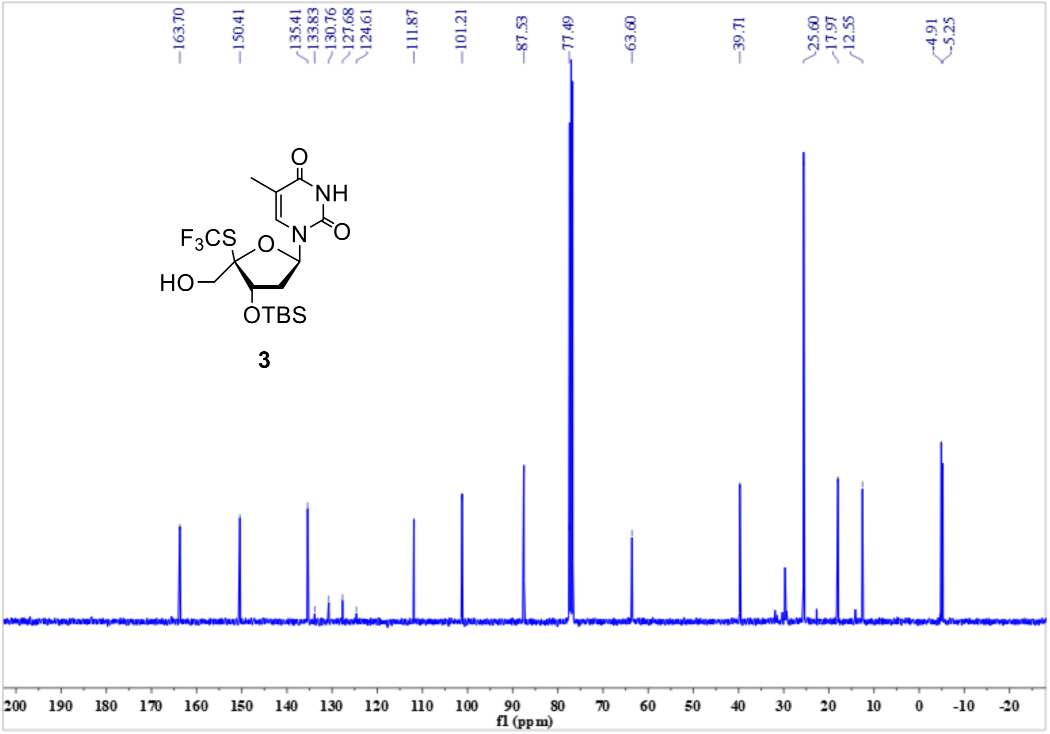

Figure S31. ^{13}C NMR (100.6 MHz, $CDCl_3$) of compound **3**.

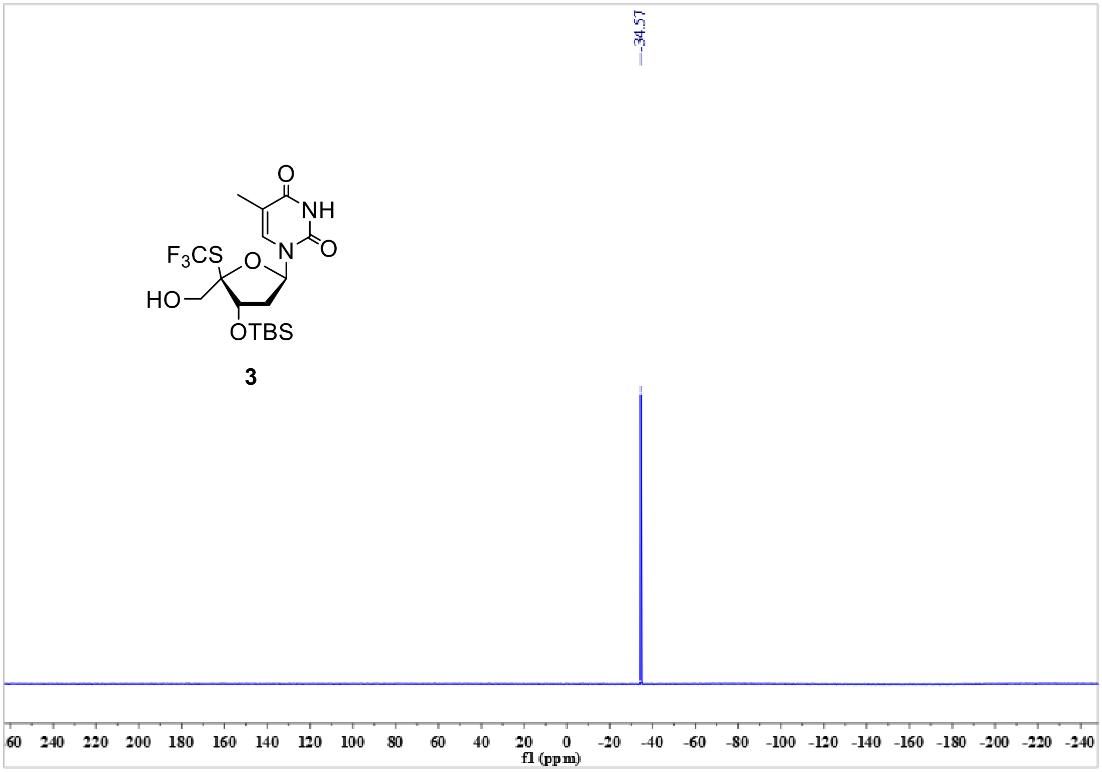

Figure S32. ^{19}F NMR (376.5 MHz, $CDCl_3$) of compound **3**

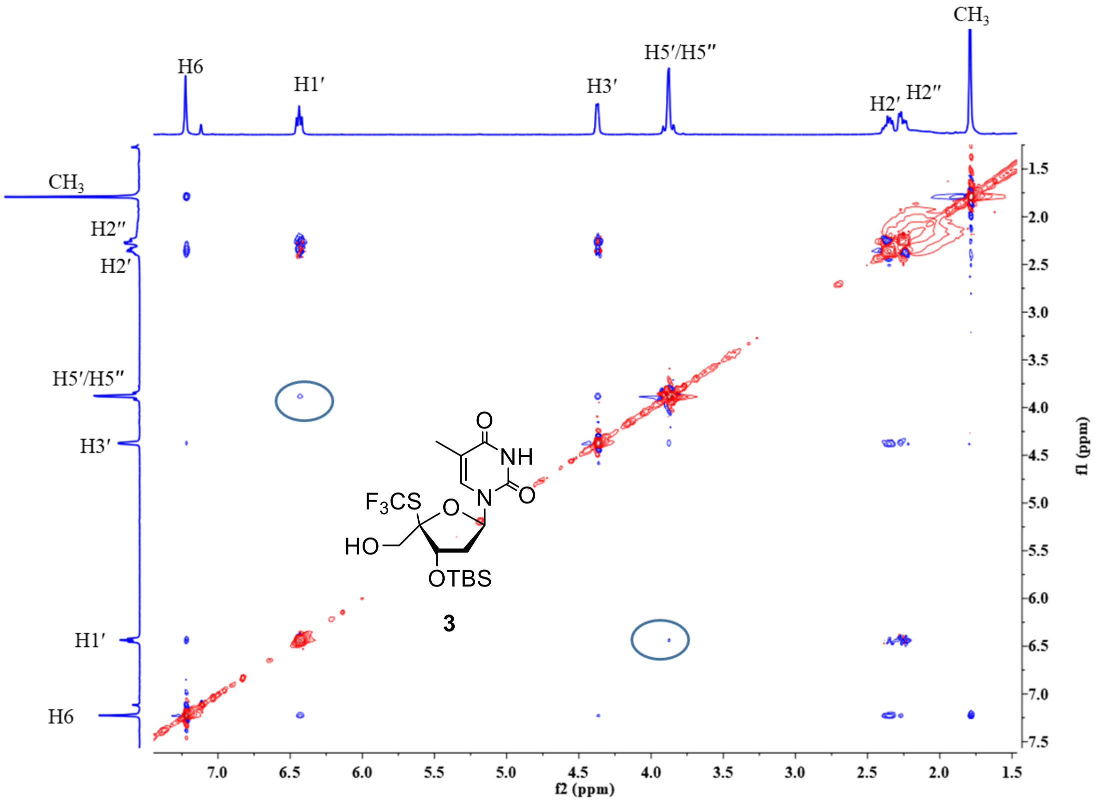

Figure S33. NOESY (400 MHz, $CDCl_3$, mixing time 400 ms) of compound **3**.

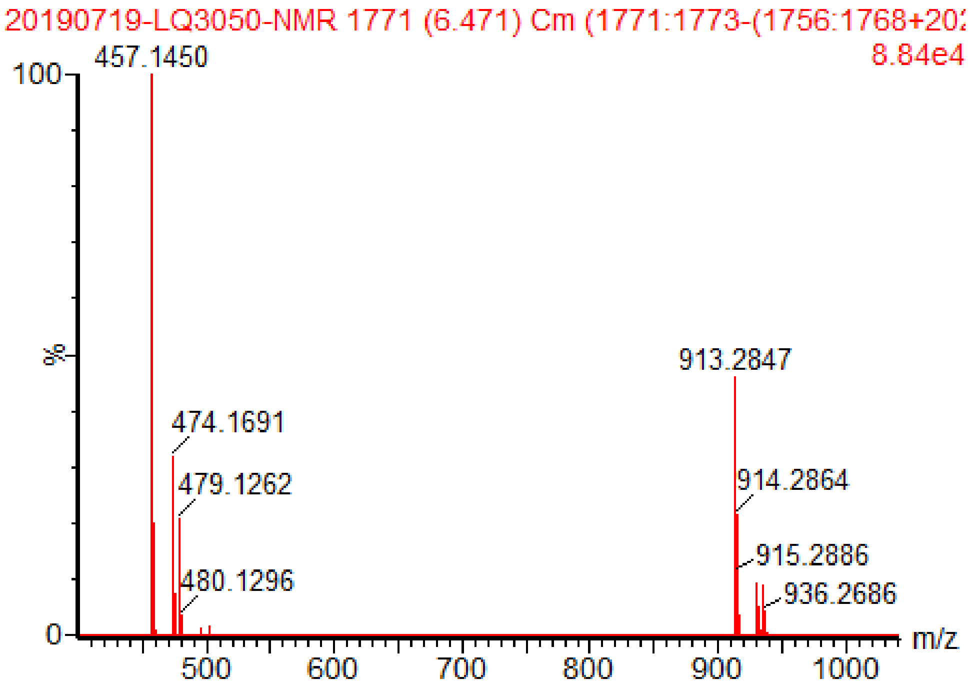

Figure S34. HRMS of compound **3**.

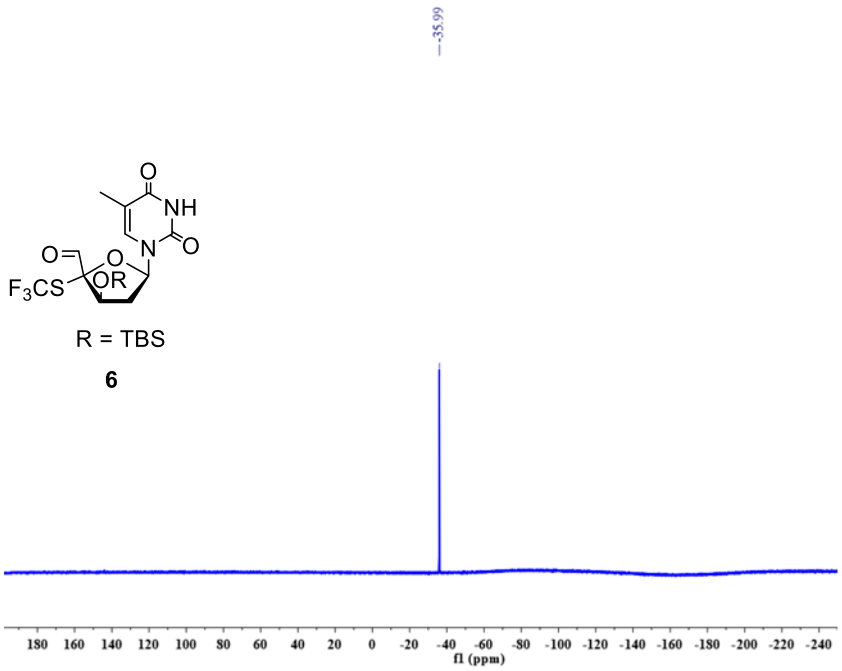

Figure S35. ^{19}F NMR (376.5 MHz, $CDCl_3$) of compound **6**.

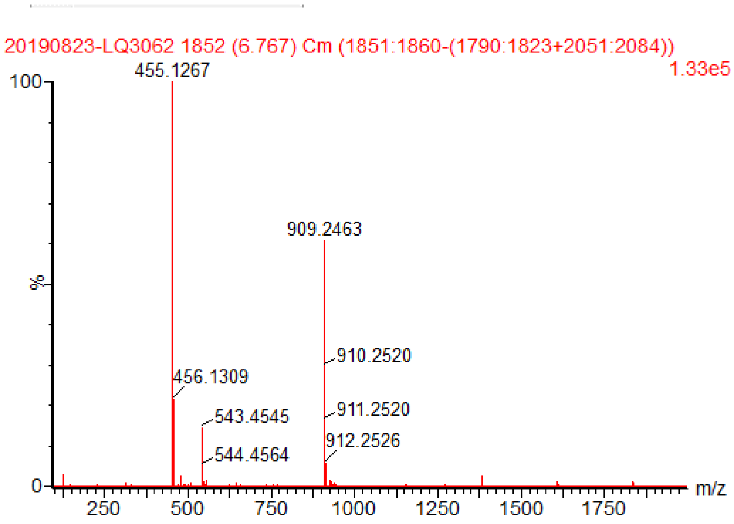

Figure S36. HRMS of compound **6**.

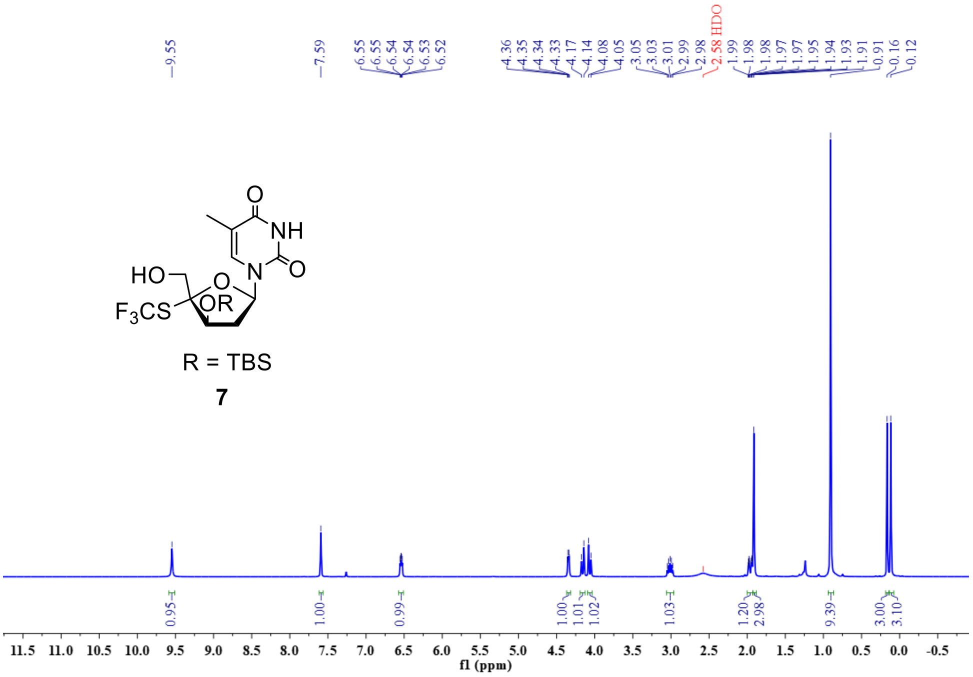

Figure S37. ^{1}H NMR (400 MHz, CDCl3) of compound **7**.

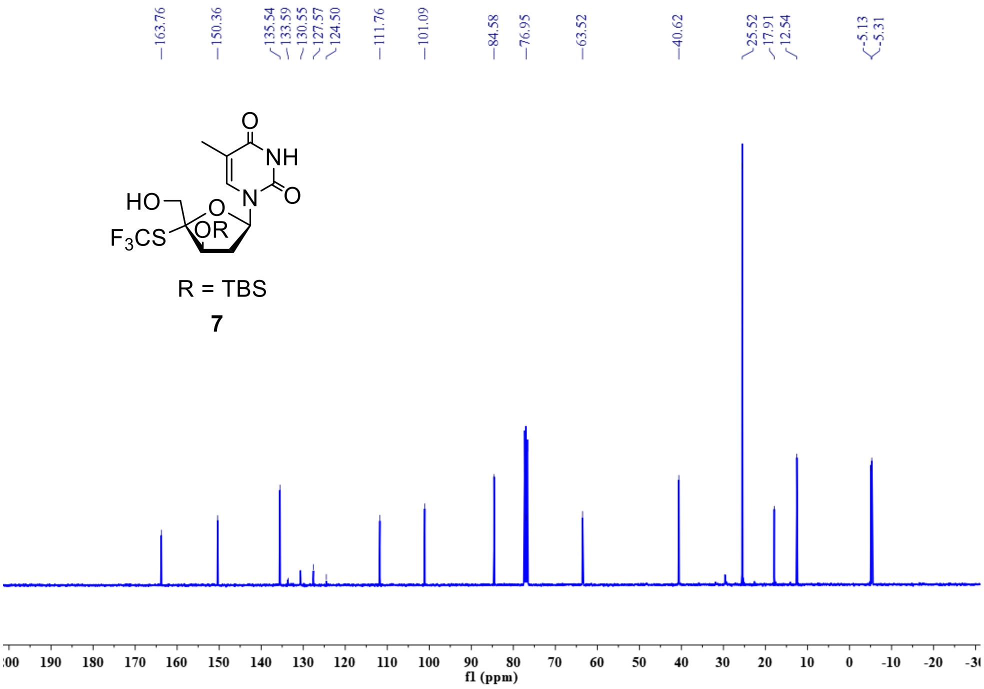

Figure S38. ^{13}C NMR (100.6 MHz, CDCl3) of compound **7**.

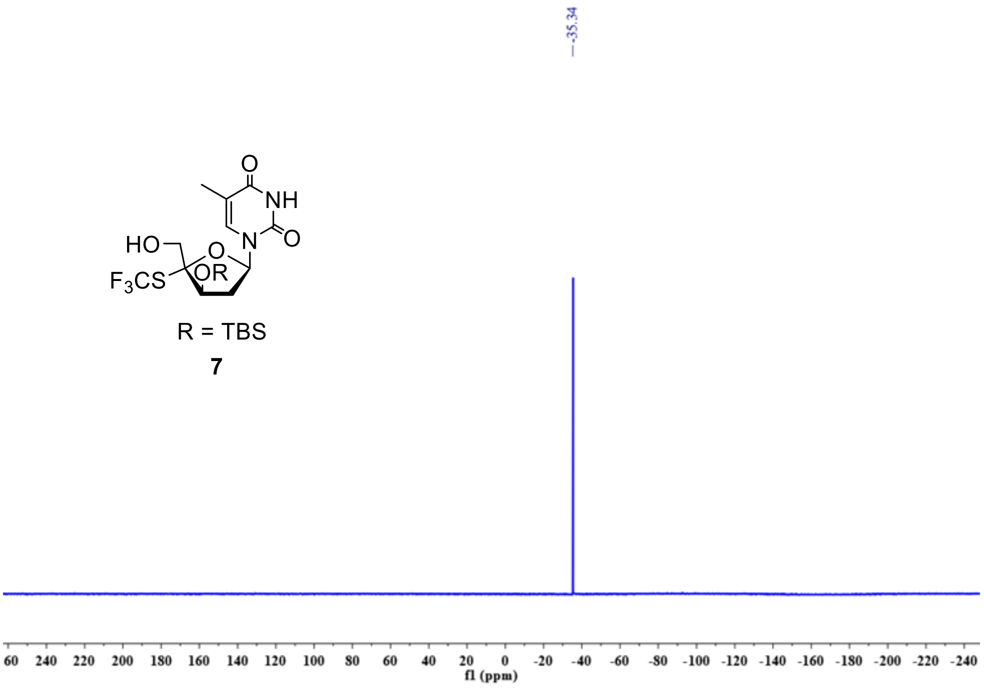

Figure S39. ^{19}F NMR (376.5 MHz, $CDCl_3$) of compound **7**.

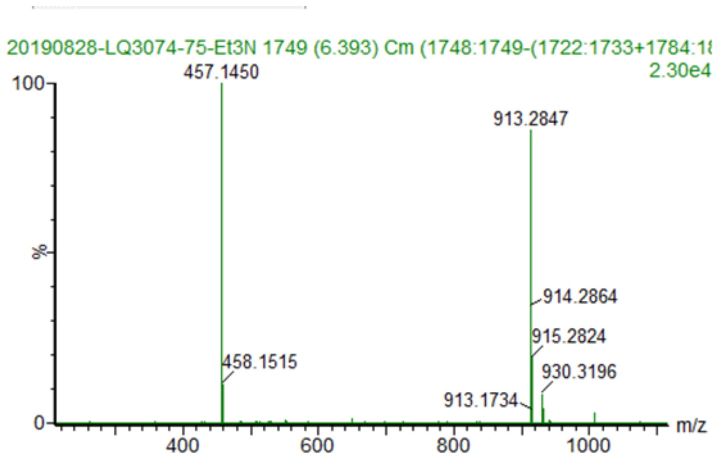

Figure S40. HRMS of compound **7**.

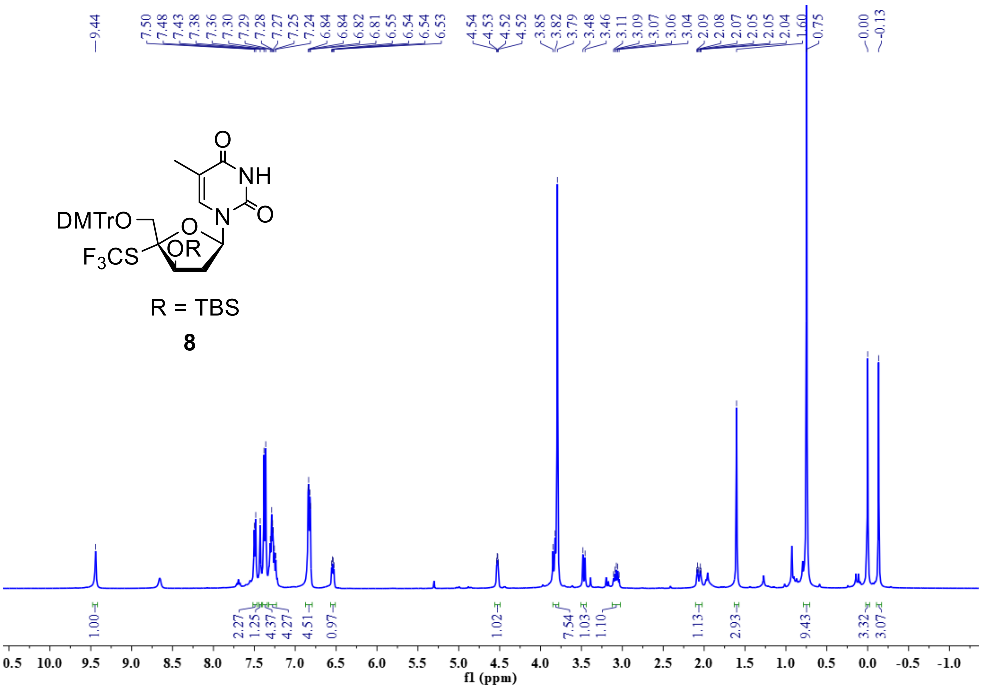

Figure S41. ^{1}H NMR (400 MHz, $CDCl_3$) of compound **8**.

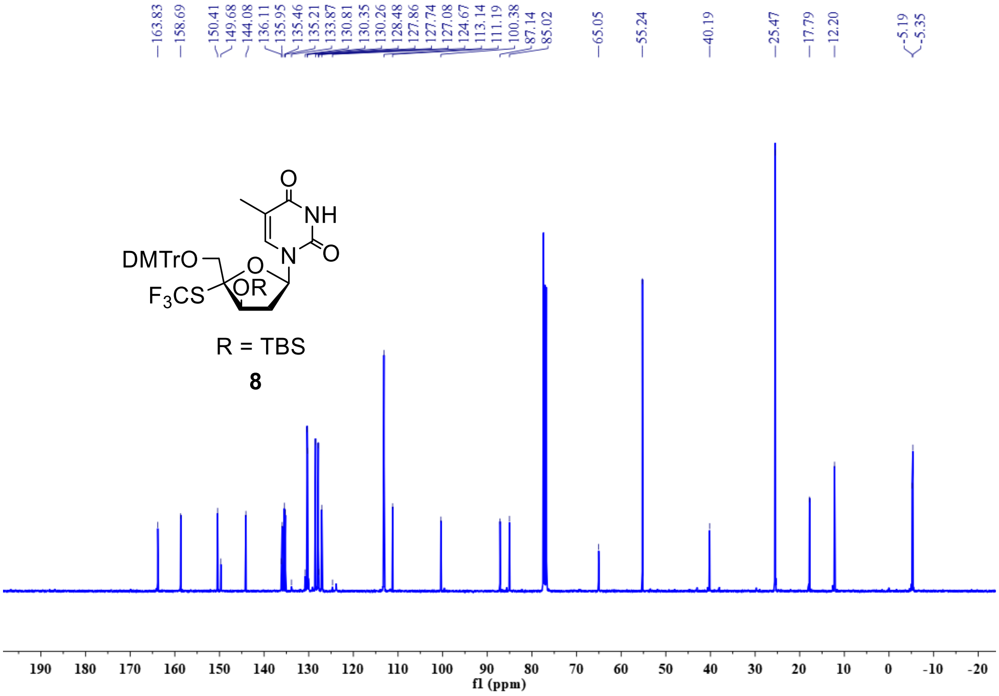

Figure S42. ^{13}C NMR (100.6 MHz, $CDCl_3$) of compound **8**.

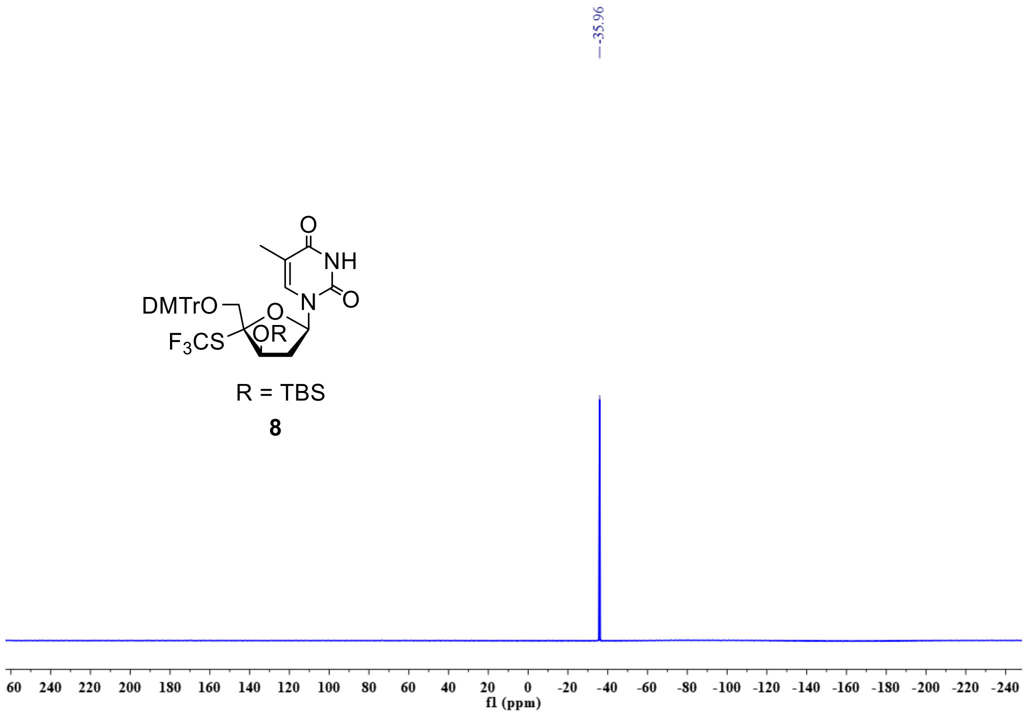

Figure S43. ^{19}F NMR (376.5 MHz, $CDCl_3$) of compound **8**.

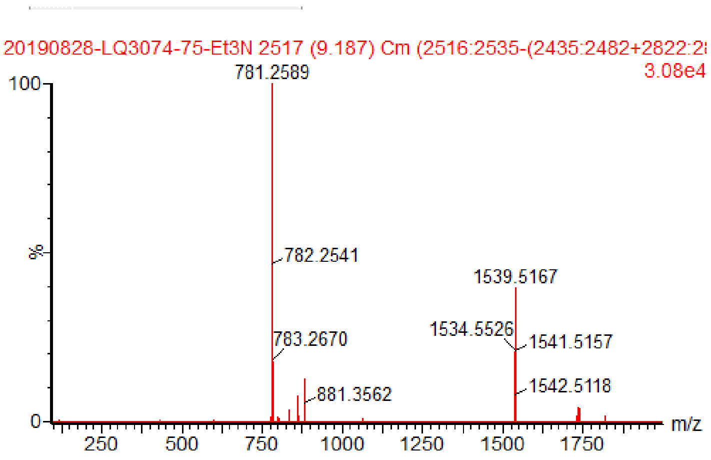

Figure S44. HRMS of compound **8**.

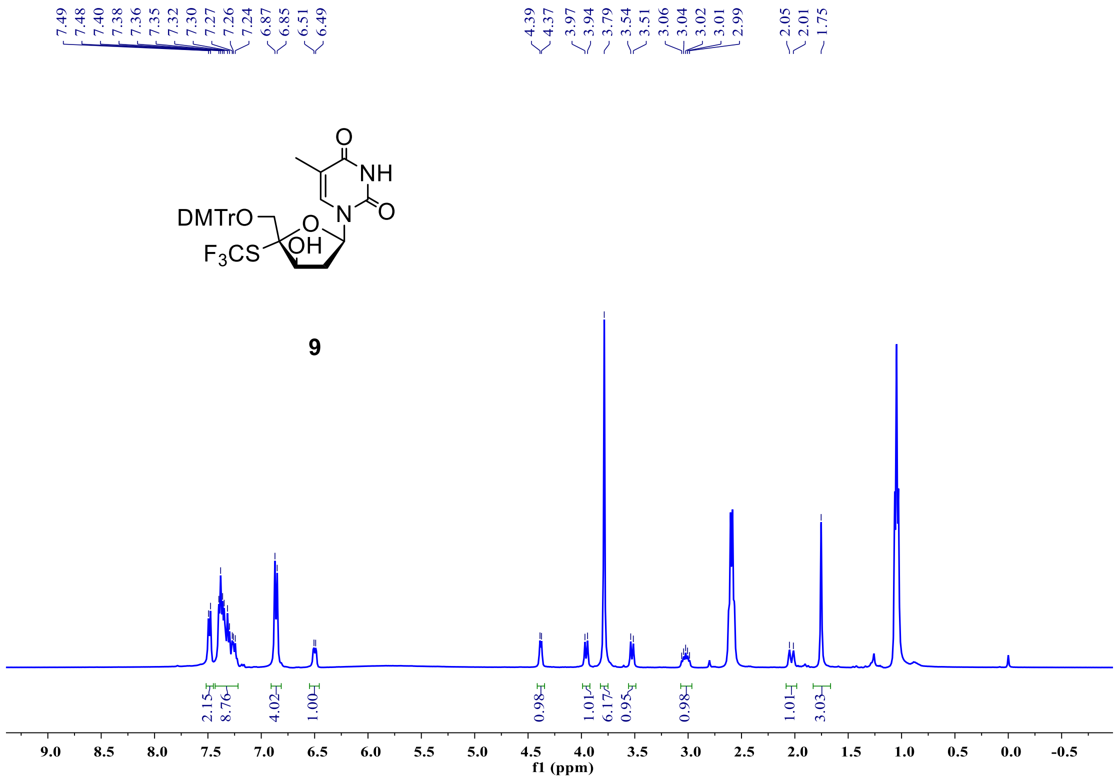

Figure S45. ^{1}H NMR (400 MHz, $CDCl_3$) of compound **9**.

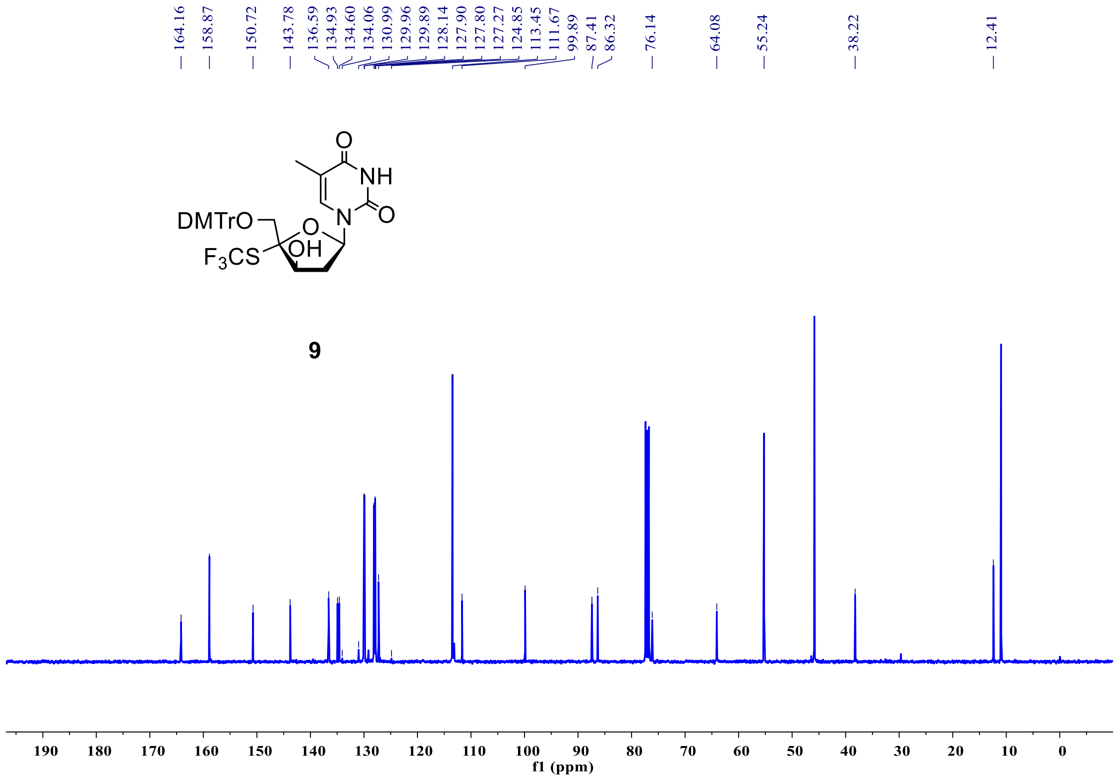

Figure S46. ^{13}C NMR (100.6 MHz, $CDCl_3$) of compound **9**.

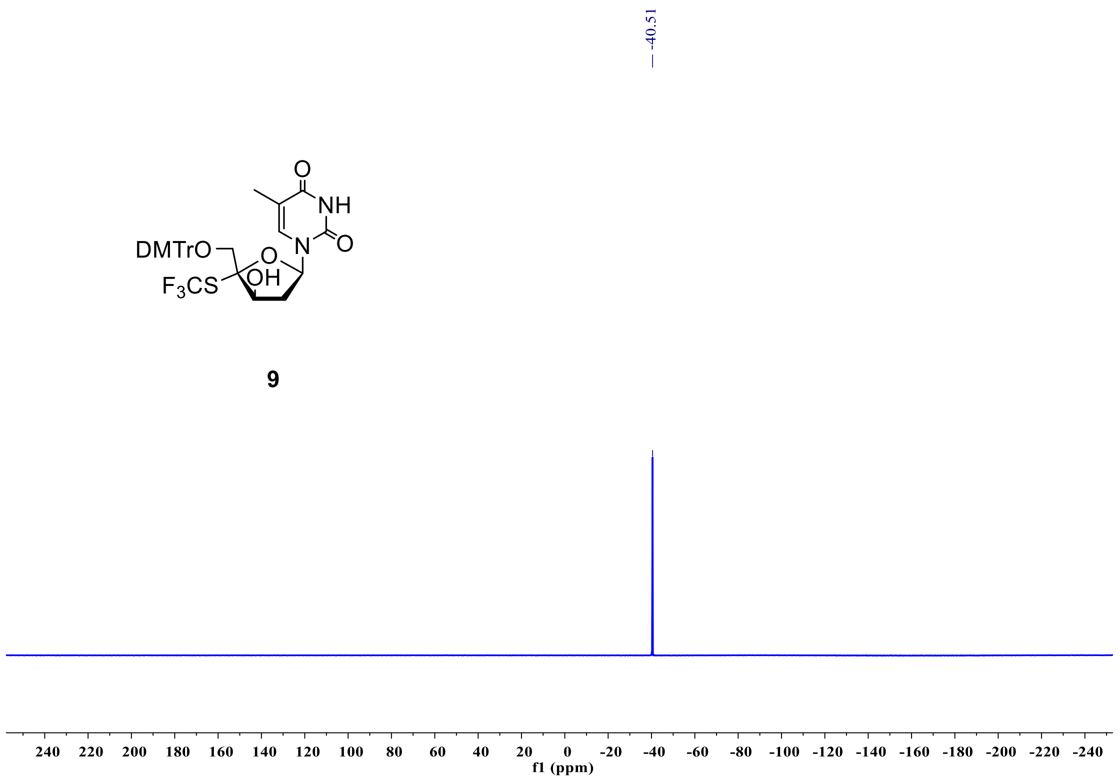

Figure S47. ^{19}F NMR (376.5 MHz, $CDCl_3$) of compound **9**.

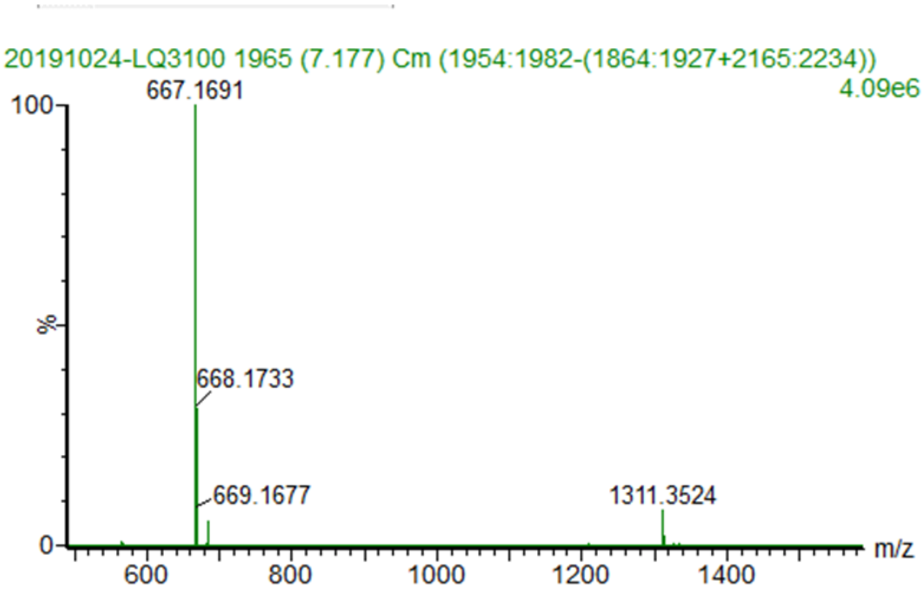

Figure S48. HRMS of compound **9**.

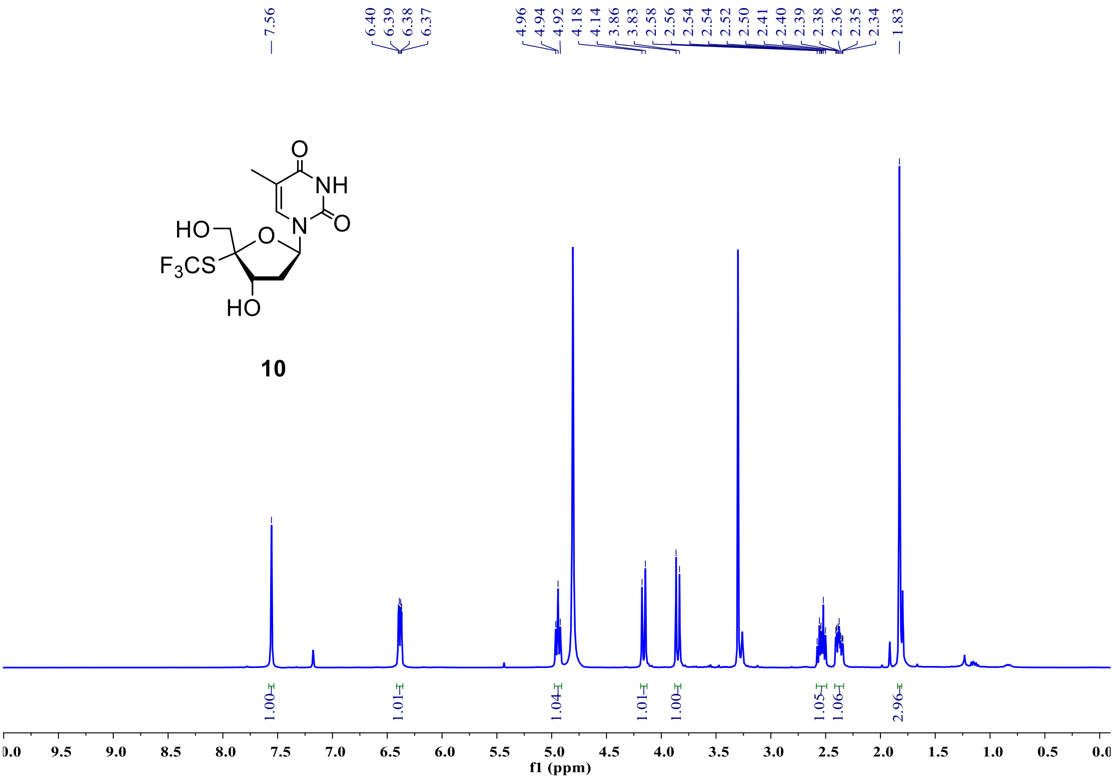

Figure S49. ^{1}H NMR (400 MHz, CD_3OD) of compound **10**.

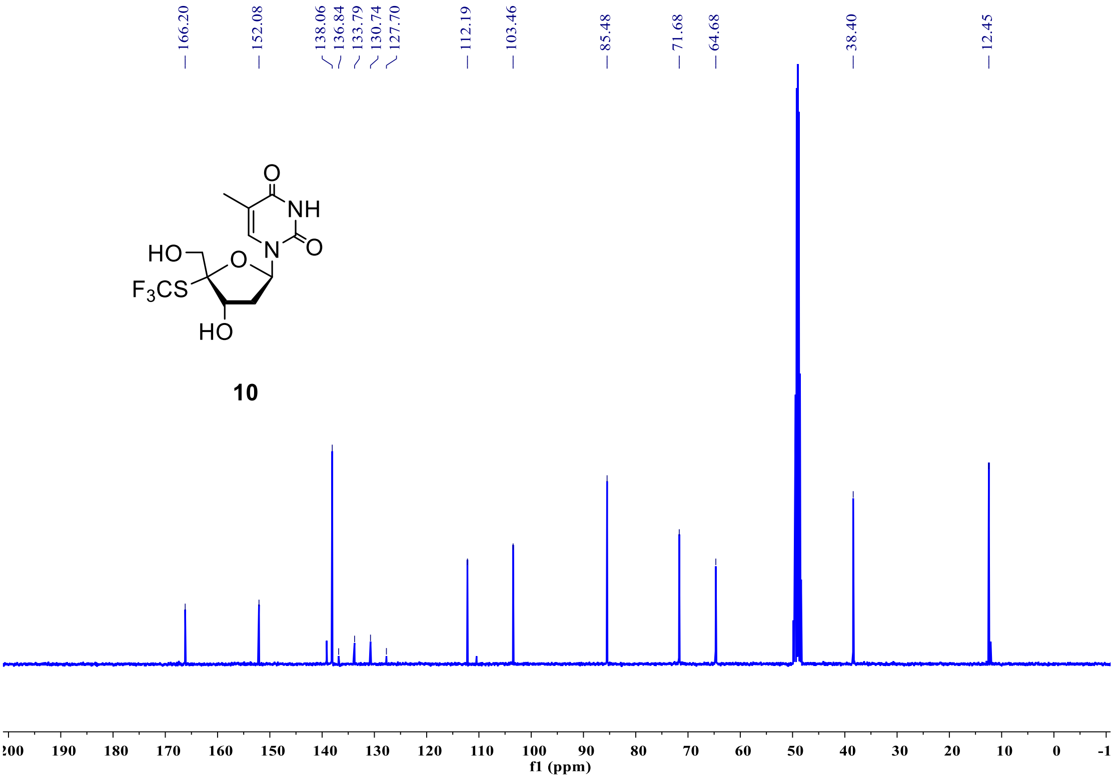

Figure S50. ^{13}C NMR (100.6 MHz, CD_3OD) of compound **10**.

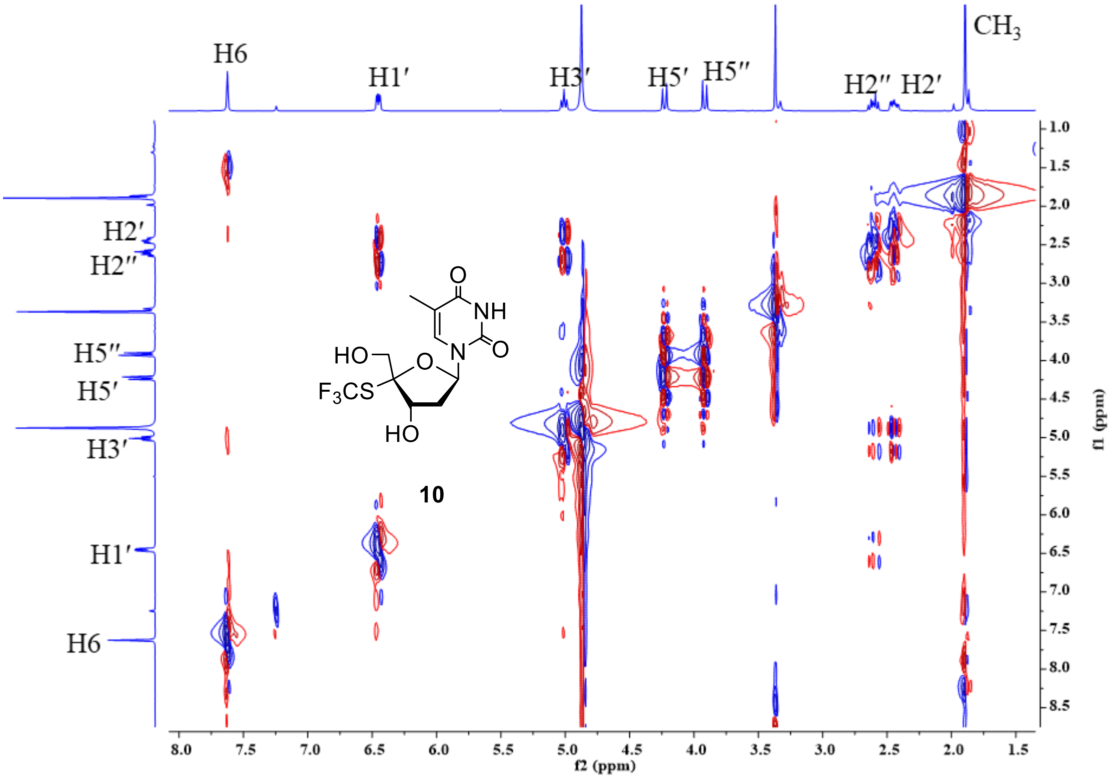

Figure S51. COSY (400 MHz, CD3OD) of compound **10**.

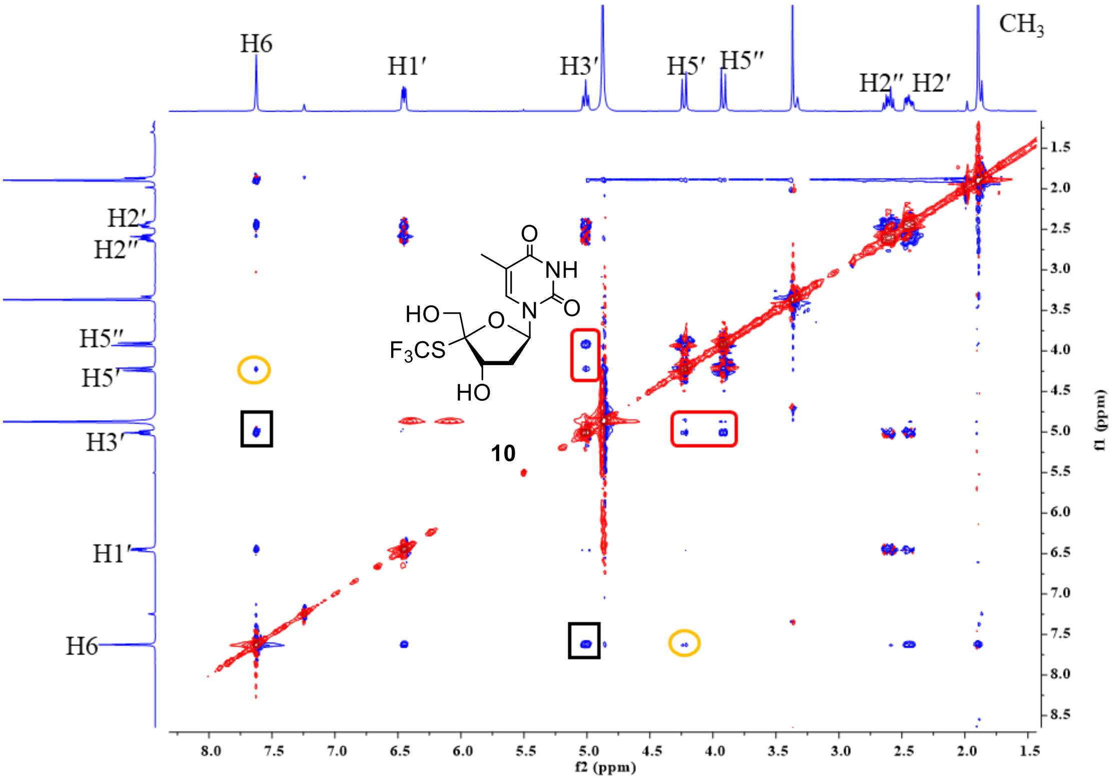

Figure S52. NOESY (400 MHz, CD3OD, mixing time 400 ms) of compound **10**.

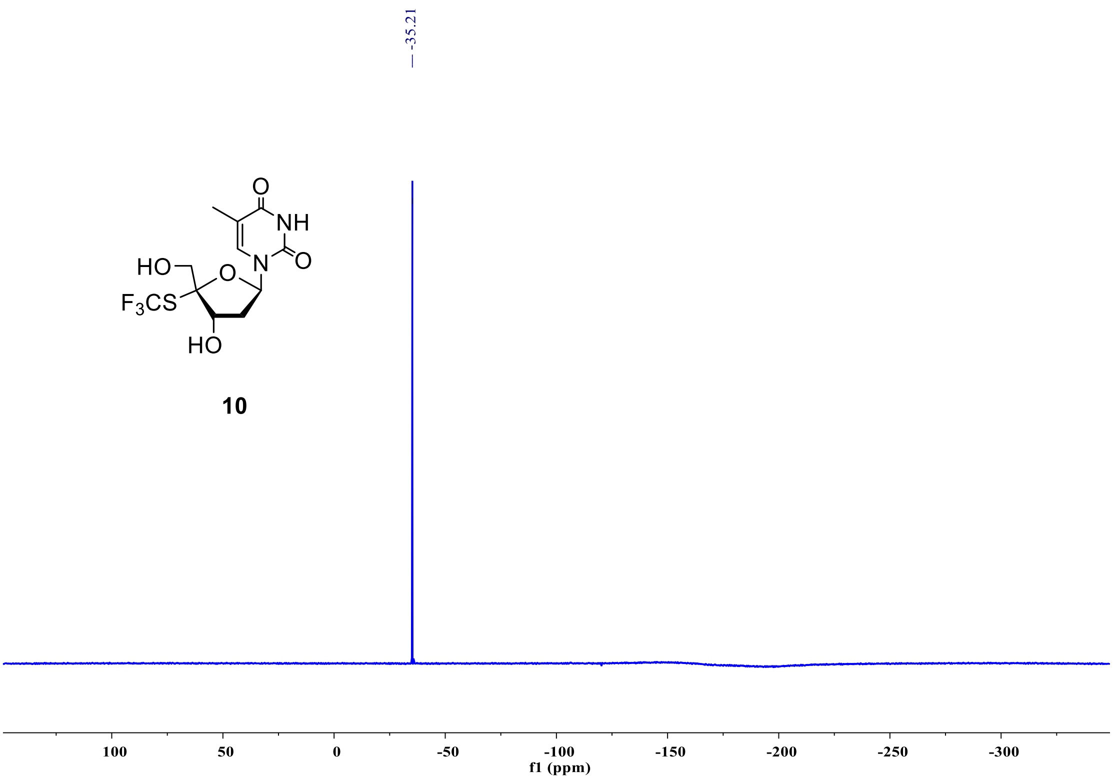

Figure S53. ^{19}F NMR (376.5 MHz, D_2O) of compound **10.**

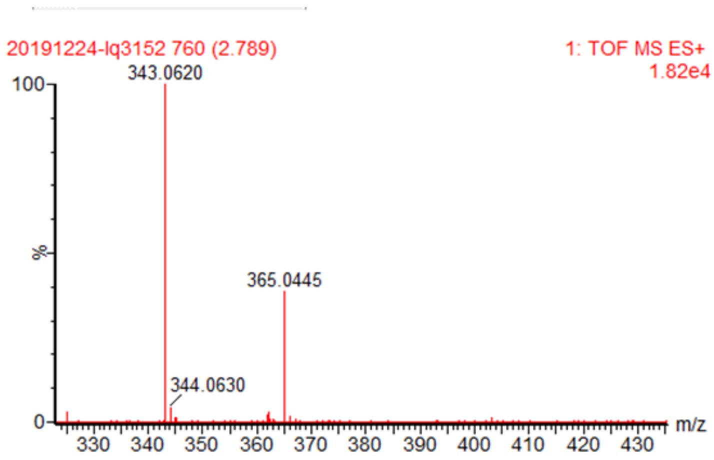

Figure S54. HRMS of compound **10**.

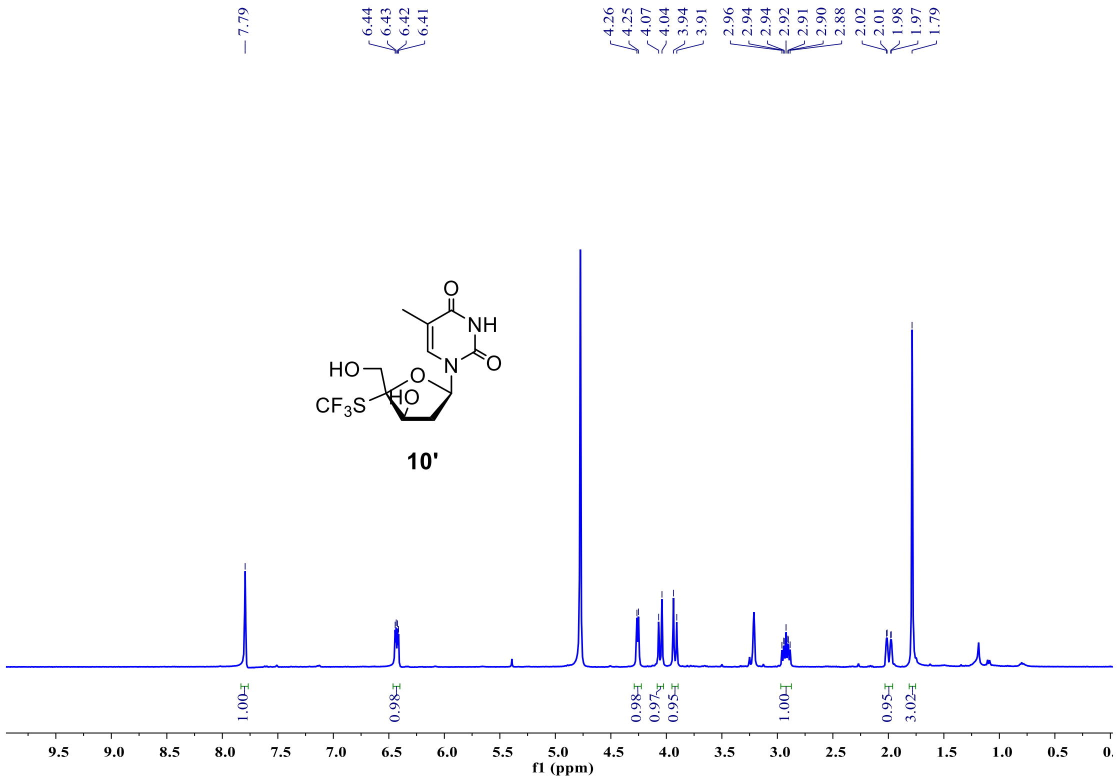

Figure S55. ^{1}H NMR (400 MHz, CD_3OD) of compound **10′**.

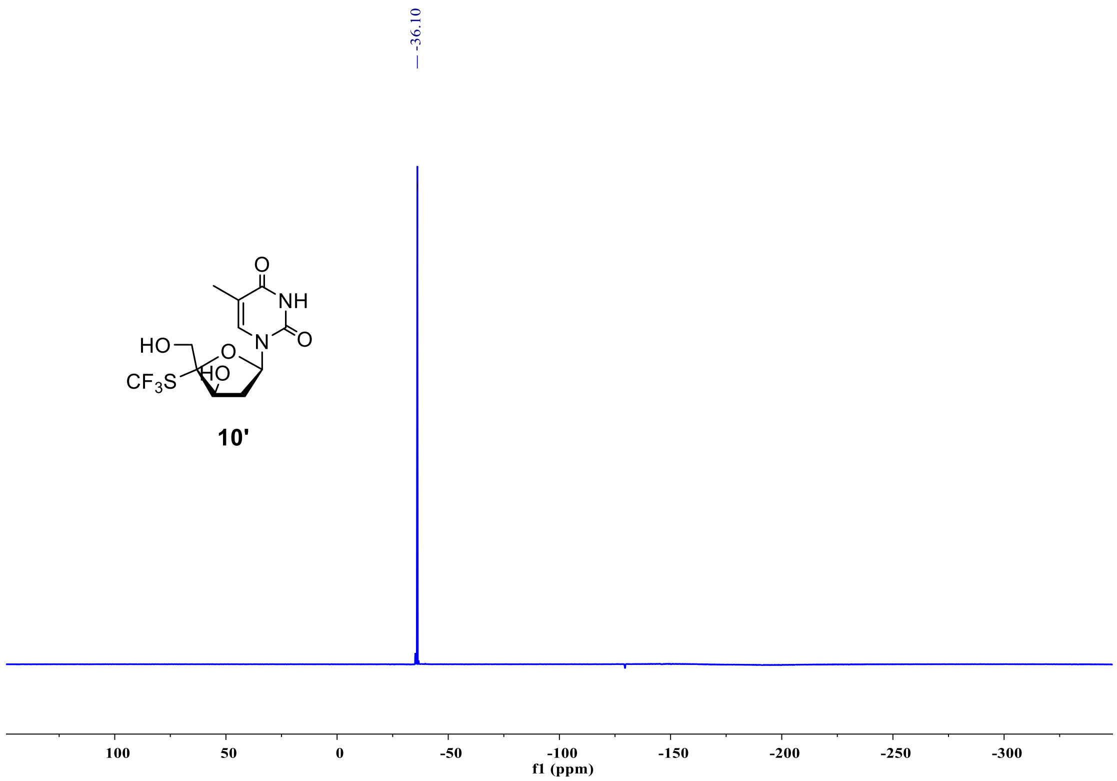

Figure S56. ^{19}F NMR (376.5 MHz, D_2O) of compound **10′**.

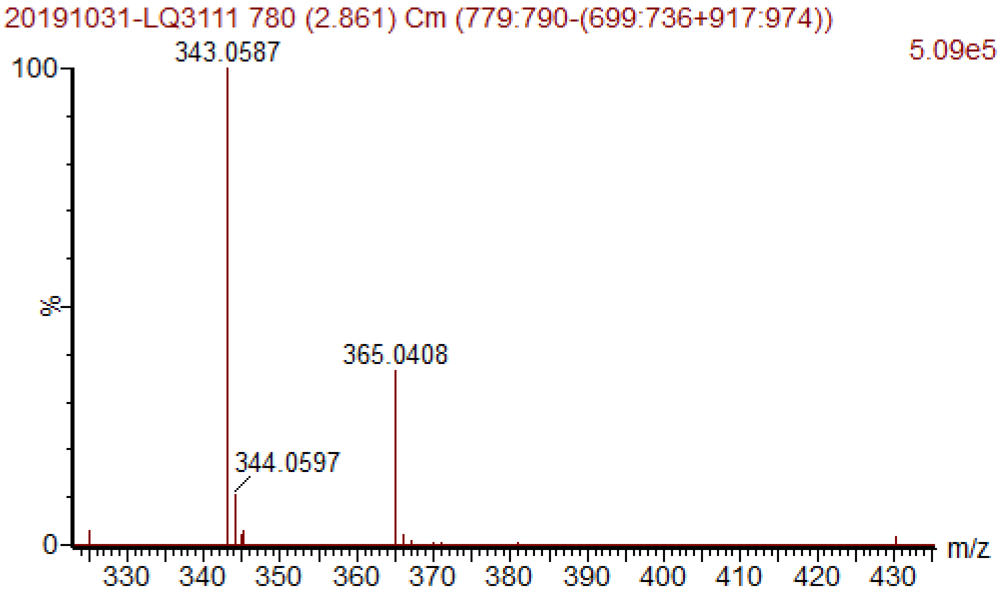

Figure S57. HRMS of compound **10′**.

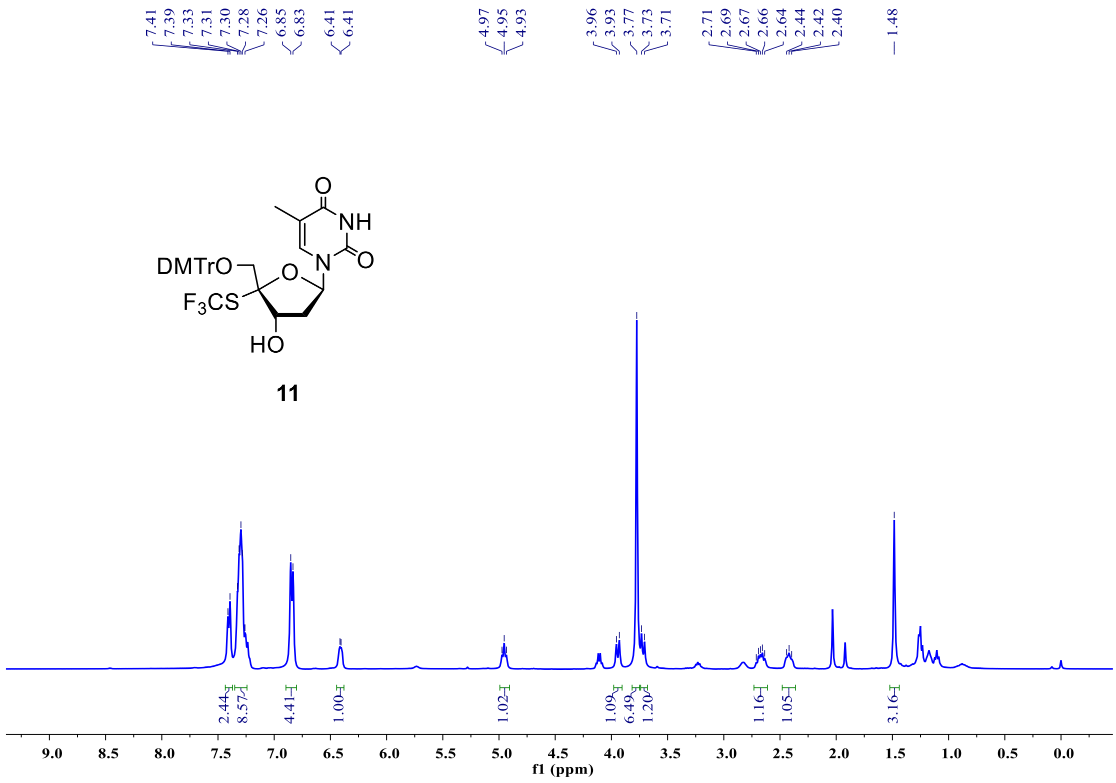

Figure S58. ^{1}H NMR (400 MHz, $CDCl_3$) of compound **11**.

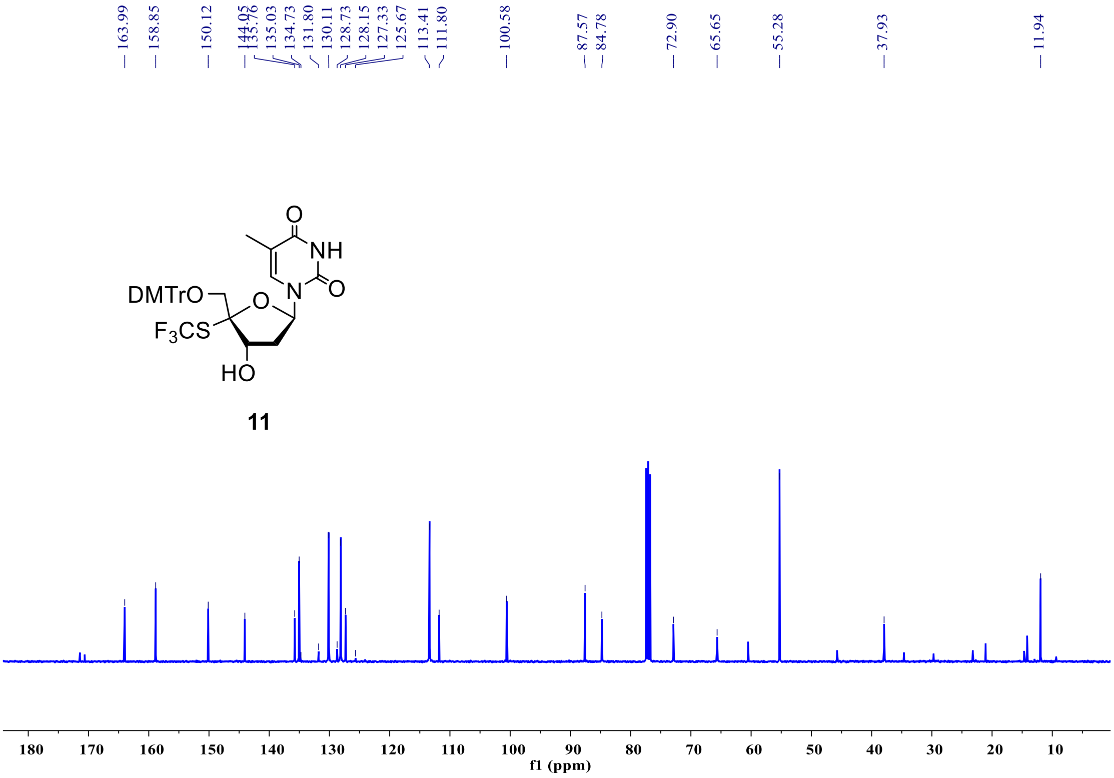

Figure S59. ^{13}C NMR (100.6 MHz, $CDCl_3$) of compound **11**.

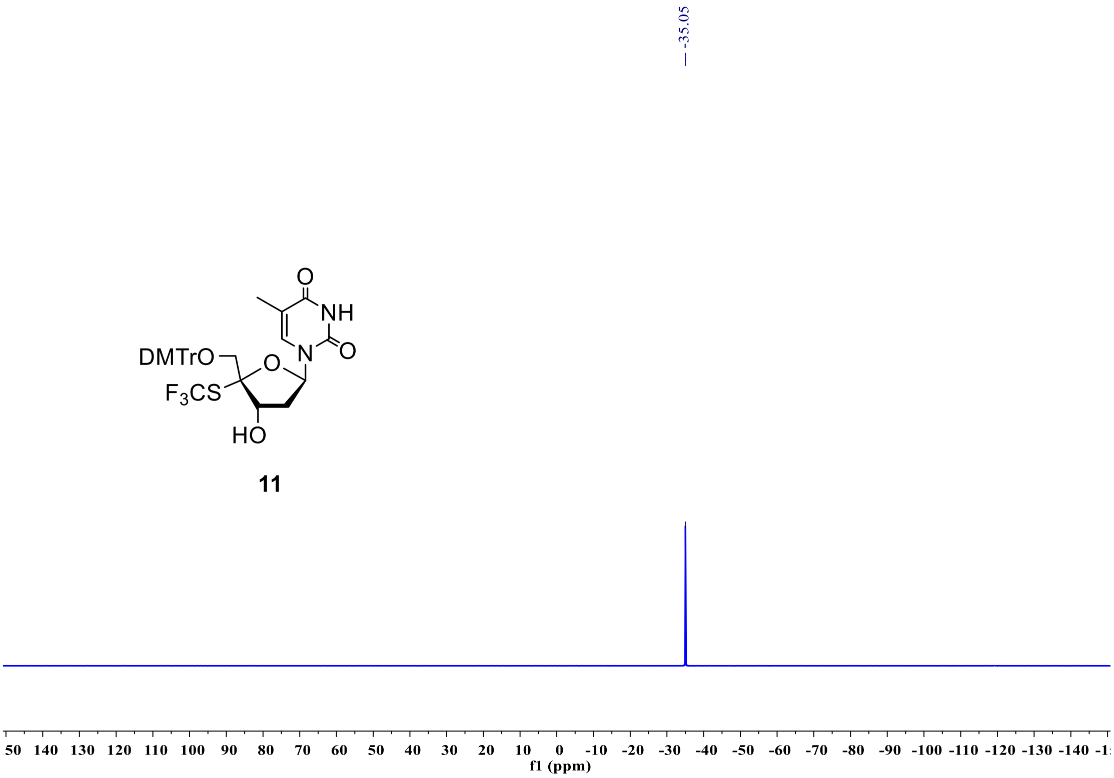

Figure S60. ^{19}F NMR (376.5 MHz, $CDCl_3$) of compound **11**.

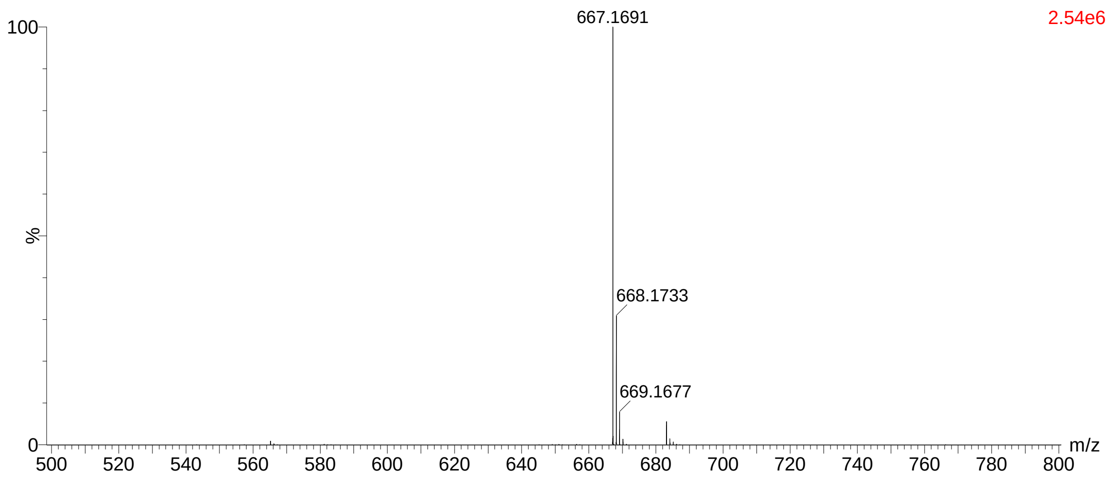

Figure S61. HRMS of compound **11**

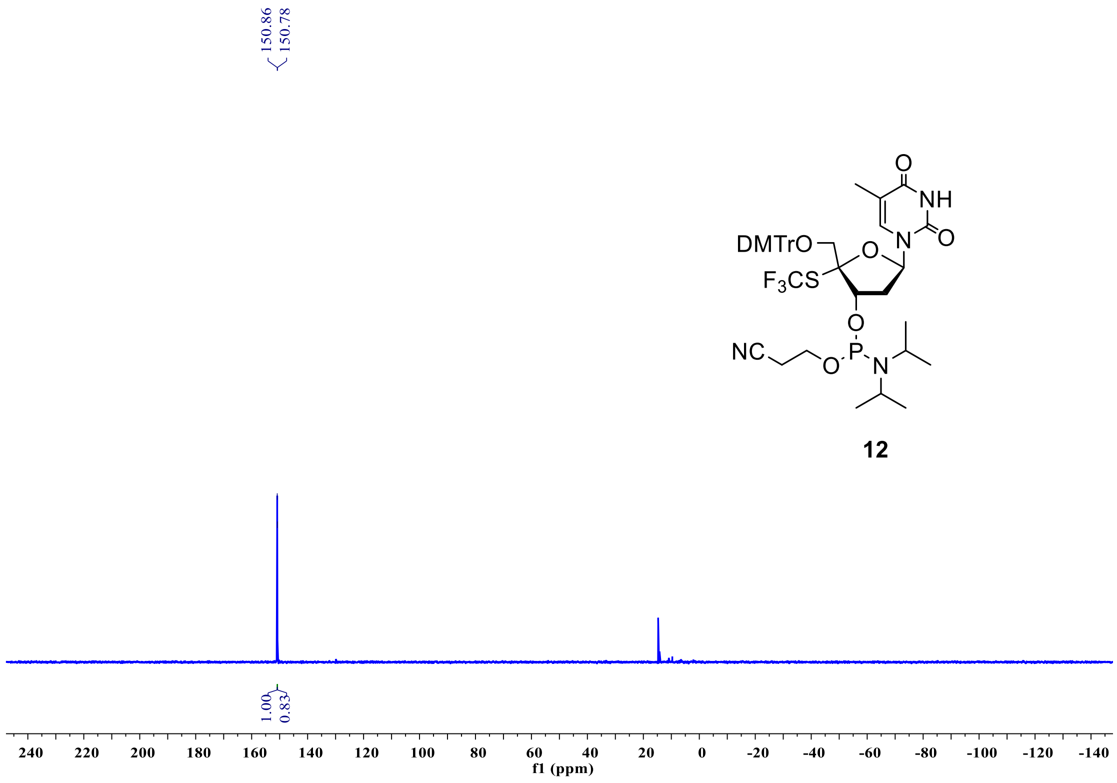

Figure S62. ^{31}P NMR (162 MHz, $CDCl_3$) of compound **12**.

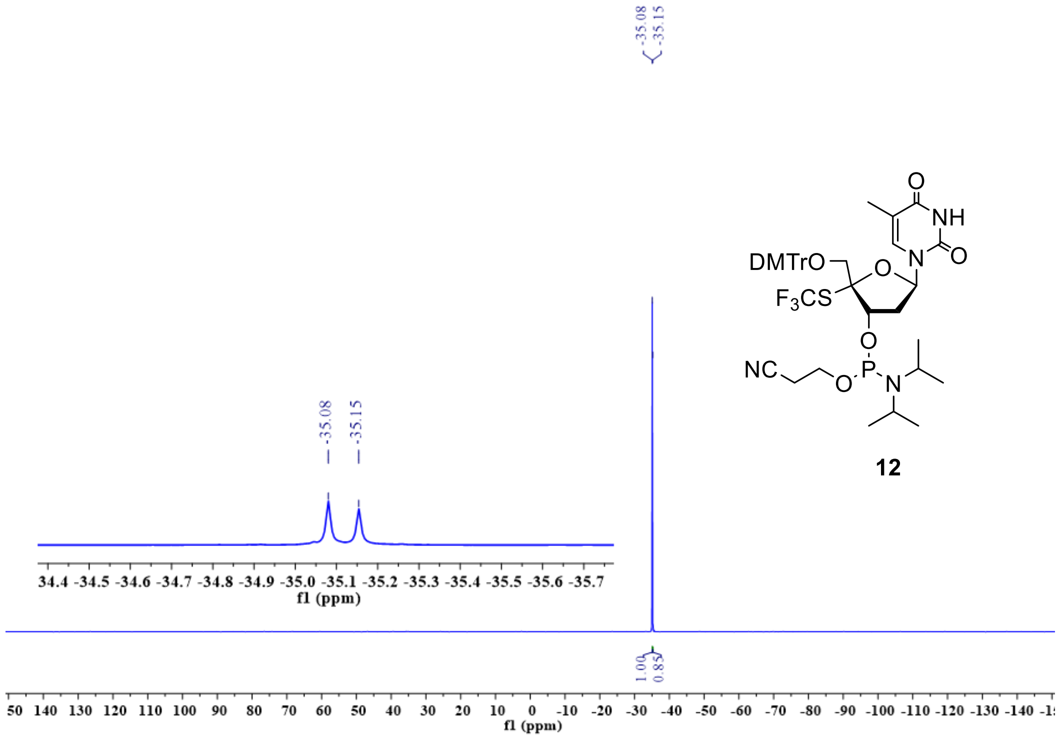

Figure S63. ^{19}F NMR (376.5 MHz, $CDCl_3$) of compound **12**

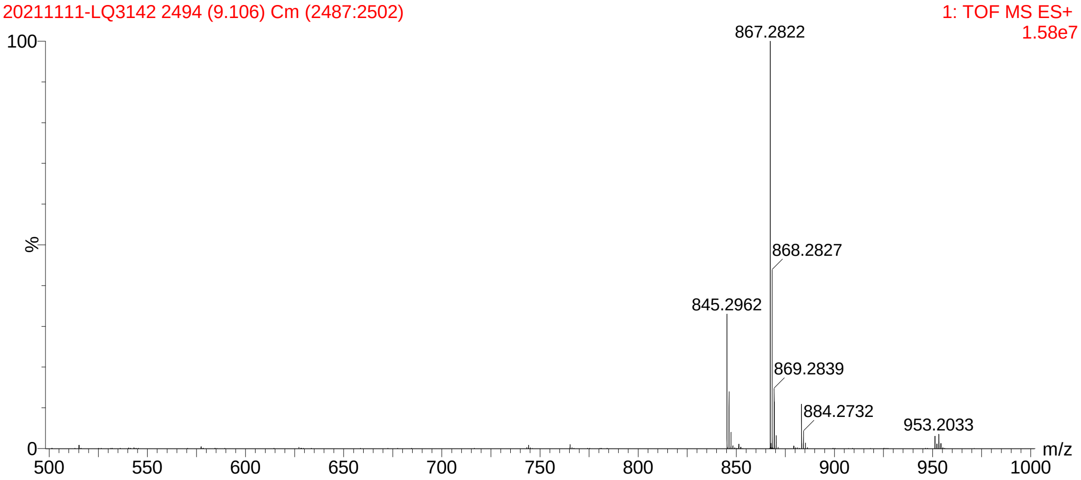

Figure S64. HRMS of compound **12.**

Supplementary References:

[1] S. S. Abubakar, M. Benaglia, S. Rossi, R. Annunziata, *Catal. Today* **2018**, *308*, 94-101.

[2] J.-L. Mergny, L. Lacroix, *Current Protocols in Nucleic Acid Chemistry* **2009**, *37*, 17.11.11-17.11.15.

[3] K. J. Breslauer, in *Methods Enzymol., Vol. 259*, Academic Press, **1995**, pp. 221-242.

[4] W. Lee, M. Tonelli, J. L. Markley, *Bioinformatics* **2015**, *31*, 1325-1327.

[5] M. Trajkovski, M. Webba da Silva, J. Plavec, *J. Am. Chem. Soc.* **2012**, *134*, 4132-4141.

[6] E. Vanquelef, S. Simon, G. Marquant, E. Garcia, G. Klimerak, J. C. Delepine, P. Cieplak, F.-Y. Dupradeau, *Nucleic Acids Res.* **2011**, *39*, W511-W517.

[7] X. J. Lu, W. K. Olson, *Nucleic Acids Res.* **2003**, *31*, 5108-5121.

[8] H. M. A. D.A. Case, K. Belfon, I.Y. Ben-Shalom, S.R. Brozell, D.S. Cerutti, T.E. Cheatham, III, V.W.D. Cruzeiro, T.A. Darden, R.E. Duke, G. Giambasu, M.K. Gilson, H. Gohlke, A.W. Goetz, R. Harris, S. Izadi, S.A. Izmailov, C. Jin, K. Kasavajhala, M.C. Kaymak, E. King, A. Kovalenko, T. Kurtzman, T.S. Lee, S. LeGrand, P. Li, C. Lin, J. Liu, T. Luchko, R. Luo, M. Machado, V. Man, M. Manathunga, K.M. Merz, Y. Miao, O. Mikhailovskii, G. Monard, H. Nguyen, K.A. O'Hearn, A. Onufriev, F. Pan, S. Pantano, R. Qi, A. Rahnamoun, D.R. Roe, A. Roitberg, C. Sagui, S. Schott-Verdugo, J. Shen, C.L. Simmerling, N.R. Skrynnikov, J. Smith, J. Swails, R.C. Walker, J. Wang, H. Wei, R.M. Wolf, X. Wu, Y. Xue, D.M. York, S. Zhao, and P.A. Kollman, *Amber* **2021**.

[9] L. J. Rinkel, C. Altona, *J. Biomol. Struct. Dyn.* **1987**, *4*, 621-649.

[10] Y. Wang, D. J. Patel, *Structure* **1993**, *1*, 263-282.
